# Structural basis of metallo-β-lactamase, serine-β-lactamase and penicillin-binding protein inhibition by cyclic boronates

**DOI:** 10.1038/ncomms12406

**Published:** 2016-08-08

**Authors:** Jürgen Brem, Ricky Cain, Samuel Cahill, Michael A. McDonough, Ian J. Clifton, Juan-Carlos Jiménez-Castellanos, Matthew B. Avison, James Spencer, Colin W. G. Fishwick, Christopher J. Schofield

**Affiliations:** 1 Department of Chemistry, University of Oxford, 12 Mansfield Road, Oxford OX1 3TA, UK; 2 School of Chemistry, University of Leeds, Leeds LS2 9JT, UK; 3 School of Cellular and Molecular Medicine, University of Bristol, Biomedical Sciences Building, Bristol BS8 1TD, UK

## Abstract

β-Lactamases enable resistance to almost all β-lactam antibiotics. Pioneering work revealed that acyclic boronic acids can act as ‘transition state analogue' inhibitors of nucleophilic serine enzymes, including serine-β-lactamases. Here we report biochemical and biophysical analyses revealing that cyclic boronates potently inhibit both nucleophilic serine and zinc-dependent β-lactamases by a mechanism involving mimicking of the common tetrahedral intermediate. Cyclic boronates also potently inhibit the non-essential penicillin-binding protein PBP 5 by the same mechanism of action. The results open the way for development of dual action inhibitors effective against both serine- and metallo-β-lactamases, and which could also have antimicrobial activity through inhibition of PBPs.

## Abstract (web summary)

Bacterial beta-lactamases are responsible for resistance to beta-lactams, the most important family of antibiotics. Here, the authors reveal cyclic boronate inhibitors that are active against both serine- and metallo-beta-lactamases and represent a promising strategy for combined antimicrobial treatments.

## Graphical abstract

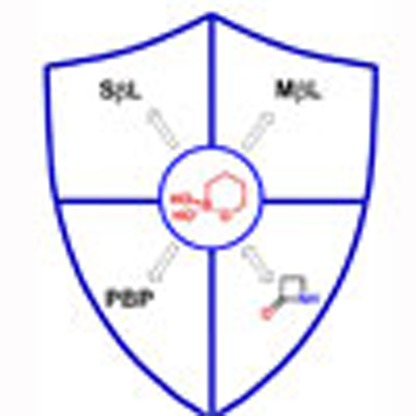

The β-lactamase-catalysed hydrolysis of β-lactam antibiotics (BLAs) is of central importance in antibiotic resistance^1^. β-Lactam-based inhibitors (for example clavulanic acid) of the Class A serine-β-lactamases (SBLs) are widely used in combination with penicillins^2^. Recently, avibactam, an inhibitor of Class A, C and some Class D SBLs, has been introduced for clinical use in combination with a cephalosporin^1^. Though not a β-lactam, avibactam is susceptible to β-lactamase-catalysed hydrolysis^1^. In contrast to SBLs, there are no clinically useful inhibitors of the Class B zinc-dependent metallo-β-lactamases (MBLs), which are of growing concern as a cause of antibiotic failure. With the exception of the monobactams, MBLs catalyse the hydrolysis of all β-lactam families including penicillins, cephalosporins, carbapenems and SBL inhibitors^3^.

SBLs and the penicillin-binding protein (PBP) targets of the β-lactams are evolutionarily and mechanistically related; as a consequence, several β-lactam classes, for example, carbapenems, can inhibit both SBLs and PBPs^4^. MBLs, however, are mechanistically and structurally distinct, and constitute a heterogeneous group^2^. The requirement for clinically useful inhibition of a broad spectrum of clinically relevant MBL subfamilies (NDM, IMP, VIM, SPM), which differ in the loops surrounding their active site, makes them challenging medicinal chemistry targets^5^.

Since many bacteria have acquired both SBL- and MBL-mediated resistance^1^, we are interested in identifying dual action MBL/SBL inhibitors. Very few potent inhibitors (IC_50_<1 μM) targeting SBLs, MBLs and/or PBPs have been developed. Since transient oxyanionic species (for example the ‘tetrahedral intermediate' of SBLs) produced by nucleophilic attack onto the β-lactam carbonyl are likely common to SBL- and MBL-catalysed β-lactam hydrolysis^3^,^6^, we reasoned analogues of this intermediate may provide the desired dual action-BL activity. While such ‘tetrahedral intermediate' analogues are well-characterized for nucleophilic enzymes, including PBPs and SBLs^2^, they have not been widely described for metallo-hydrolases. The observation of MBL inhibition by trifluoromethyl ketones^7^ is evidence that mimicking a tetrahedral intermediate may also be useful for the inhibition of MBLs. Since acyclic boronic acids, are established as SBL/PBP inhibitors^1^ (the SBL inhibitor, RPX7009 (ref. 1), is in clinical trials), we screened various boronic acids, including some reported to be SBL/PBP inhibitors, for inhibition of the NDM-1 MBL. Interestingly, cyclic boronates, but not the acyclic boronic acids, manifested potent MBL inhibition. We therefore synthesized and tested additional boronic acids, including compounds (**2, 4** and **5**) described in the patent literature as β-lactamase inhibitors^8^ and novel derivatives **1** and **3** (designed using modeling).

We demonstrate through biochemical, biophysical and cellular evidence that cyclic boronates are potent inhibitors of both SBLs and MBLs. Interestingly, we also found that the cyclic boronates inhibit the PBP targets of the BLAs. High-resolution crystallographic analyses reveal the proposed mechanism of action. The cyclic boronates act as ‘transition state analogues' for both ‘serine' and ‘metallo' enzymes and therefore represent a promising strategy for combating antibiotic resistance.

## Results

### MBL inhibition by cyclic boronates

**Figure 1:**
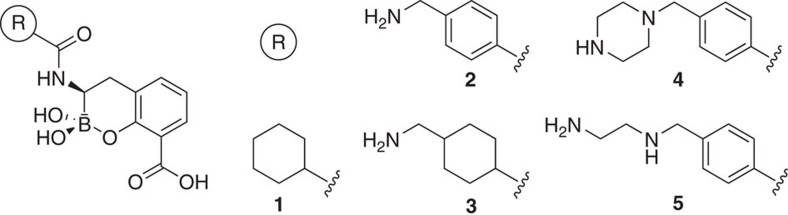


Using a fluorogenic assay for MBLs^9^, we screened the cyclic boronates (Fig. 1) against a representative panel of clinically relevant B1 subfamily MBLs, including IMP-1 (Imipenemase-1), VIM-2 (Verona-Integron-Encoded MBL-2), NDM-1 (New Delhi MBL-1), SPM-1 (São Paulo MBL-1) and the model MBL, BcII from *Bacillus cereus*^2^. The results imply that cyclic boronates with an aromatic side chain, positioned analogously to the 6β/7β side chains of the penicillins/cephalosporins, are potent inhibitors of B1 MBLs (for example, pIC_50_ values for **5** range from 8 to 9 against VIM-2 and NDM-1; Table 1). In support of these findings, ^19^F-protein NMR studies, employing site-selective labelling of M67C NDM-1 with 1,1,1-trifluoro-3-bromo acetone^10^, reveal tight binding of **2** to the active site of the NDM-1 MBL (Supplementary Fig. 1). *In vitro* inhibition of MBLs by the tested cyclic boronates yielded the following rank order of potency: VIM-2>NDM-1>BcII>IMP-1>SPM-1 (Table 1). As SPM-1 (a ‘hybrid' enzyme with properties of both the B1/B2 MBL subfamilies^11^) was inhibited least strongly (IC_50_ 13–36 μM), we investigated inhibition of *Aeromonas hydrophilia* CphA^12^ as a representative of the mono-Zn(II) B2 MBL subfamily and observed similar inhibition potency (high μM range, Table 1), suggesting that the tested cyclic boronates may be less potent against B2 MBLs. Overall, these data identify **2** and **5** as highly potent inhibitors of VIM-2 and NDM-1, respectively, the most widely distributed members of the clinically important B1 subfamily (Table 1).

### SBL and PBP inhibition by cyclic boronates

We then used fluorogenic assays^9^ to measure the potency of the cyclic boronates against clinically relevant Class A and Class D SBLs, including TEM-1 (Class A) and OXA-10 (Class D)^13^. All of the compounds tested were potent TEM-1 inhibitors (IC_50_ 6→0.3 nM, Table 1) and compounds with saturated side chains (**1** and **3**) manifested IC_50_ values<1 μM against OXA-10 (Table 1). Although our initial objective was to identify and characterize compounds potent against both MBLs and SBLs, the observed potency of the cyclic boronates versus SBLs motivated us to also investigate their potential for inhibition of a mechanistically-related PBP.^14^ PBP 5 (dacA) from *Escherichia coli*, which is a non-essential PBP^15^, was potently inhibited by all tested cyclic boronates (residual activity<1% at 10 μM; IC_50_ for **2**, 1.7 nM). We also tested the essential PBP 3 from *Pseudomonas aeruginosa* at 100 μM against the cyclic boronates, but no inhibition was detected (Table 1). These results reveal the potential for cyclic boronates to act as broad-spectrum inhibitors of SBLs and MBLs with activity against, at least some, PBPs.

### Pathogen susceptibility to cyclic boronate

Since **2** was a potent inhibitor of all three enzyme classes *in vitro*, we next tested its activity against bacterial cells (the Gram-negative pathogens *E. coli* and *Klebsiella pneumoniae*) in antimicrobial susceptibility assays (Table 2). **2** was evaluated against multiple strains of both organisms and, alone and in combination with the carbapenem meropenem, against recombinant *K. pneumoniae*^16^ and previously described *K. pneumoniae* and *E. coli* strains of clinical origin.^17^ These strains all carry the NDM-1 MBL, together with multiple SBLs of different classes^17^. **2** alone did not display antibacterial activity against any of the strains tested at concentrations up to 128 μg ml^−1^ (for *E. coli* ATCC 25922 and *K. pneumoniae* NCTC 5055, data not shown). The minimal inhibitory concentrations (MICs) determined for NDM-1-expressing *K. pneumoniae* Ecl8 in the presence of compound **2** at concentrations ranging from 0.5 to 64 μg ml^−1^ demonstrated a dose-dependent increase in susceptibility as the concentration of **2** increased (Supplementary Table 1). For all the strains producing NDM-1, co-administration with **2** reduced the MIC of meropenem. Clear reductions in meropenem MIC were observed at 10 μg ml^−1^ **2**. At a concentration of 25 μg ml^−1^, **2** brought the meropenem MIC into the susceptible range (MIC<8 μg ml^−1^) for recombinant *K. pneumoniae* strain Ecl8 and its Δ*ramR* mutant derivative (which has reduced envelope permeability through reduced porin and increased efflux pump production) when both are producing NDM-1. Neither **1** nor **2** showed cytotoxicity in human HEK293 cells when administered at concentrations up to 100 μM (Supplementary Fig. 2).

### Structural analysis of cyclic boronate binding to BcII and VIM-2

We then investigated the mechanism of MBL inhibition by **2** using X-ray crystallography. We obtained high-resolution structures for VIM-2 and BcII in complex with **2** to 1.5 Å and 1.9 Å resolution, respectively (Supplementary Figs 3–6). In both these structures, the electron density for the inhibitor clearly defines the geometry of the boron as tetrahedral (sp^3^).

**Figure 2:**
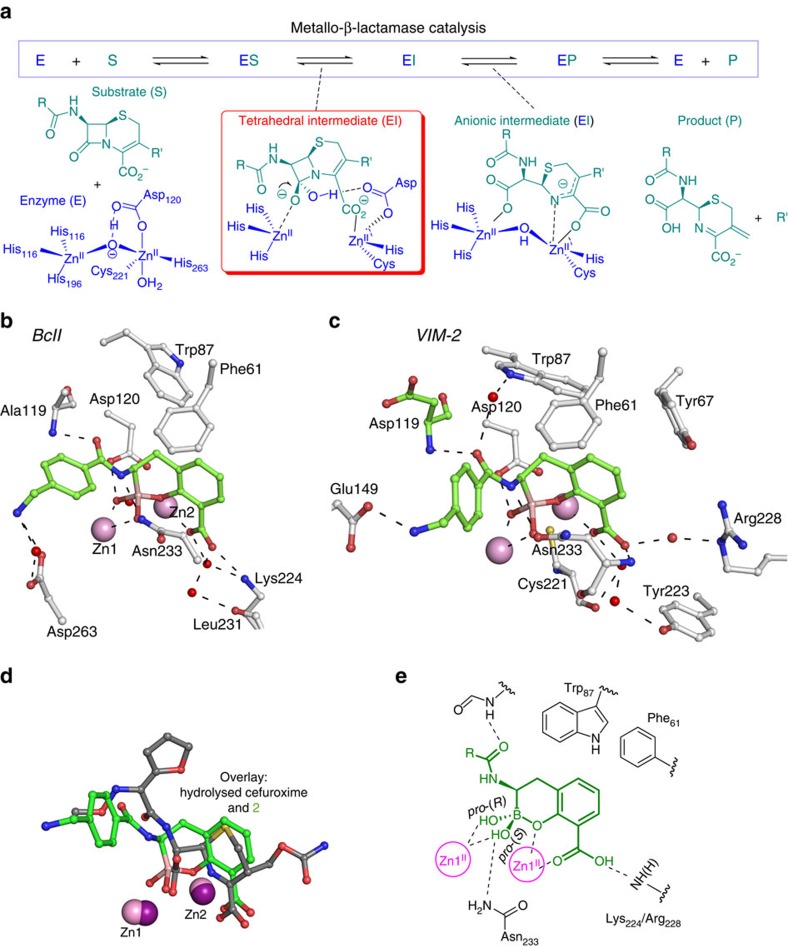
Mode of action of metallo-β-lactamases and binding mode of cyclic boronates. (**a**) Outline mode of action of MBLs showing proposed intermediates. (**b**,**c**) Views from structures obtained by co-crystallization of **2** with BcII (**b**) and VIM-2 (Chain A) (**c**). Note the relative position of Trp_87_ is rotated by ∼180° compared with its position in structures of BcII and VIM-2 without inhibitor; in the case of VIM-2, Trp_87_ interacts with the cyclic boronate via a water molecule. (**d**) The overlay compares the binding modes of **2** and hydrolysed cefuroxime in complex with NDM-1 (PDB ID: 4RL2)). (**e**) Key active site interactions made by the cyclic boronates.

For the B1 subfamily MBLs, β-lactam hydrolysis is proposed to proceed via nucleophilic attack of a hydroxide ion onto the β-lactam carbonyl; in ground state structures, this hydroxide bridges the two active site zinc ions (Zn1 and Zn2)^17^. A transient tetrahedral oxyanionic species (Fig. 2a) is formed that reacts to give a Zn1-bound carboxylate and a, sometimes detectable, intermediate^18^ in which the negatively charged β-lactam-derived nitrogen coordinates to Zn2; protonation of this intermediate is required for product release. The mode of binding of **2** observed here, in which both boron-bound oxygen atoms participate in bidentate coordination of the Zn1 ion, most closely resembles that predicted for the tetrahedral oxyanion formed during β-lactam hydrolysis, with the implication that these inhibitors act as mimics of this state (Fig. 2b,c).

The structures of both BcII and VIM-2 in complex with **2** reveal binding modes similar in some key aspects to those observed for complexes of B1 MBLs with hydrolysed β-lactams, in particular for a ring-opened cephalosporin intermediate^19^ (Fig. 2d). First, one oxygen atom of the inhibitor C-3 carboxylate coordinates to Zn2; the other carboxylate oxygen interacts with Lys_224_ (NDM-1 and BcII) or Arg_228_ (VIM-2) by hydrogen-bonding/electrostatic interactions. These interactions are analogous to those with hydrolysed β-lactams where the C-3/C-4 carboxylate (of penicillins/cephalosporins, respectively) binds Zn2 and Lys_224_ (BcII and NDM-1) or Arg_228_ (in the VIM family) (Fig. 2b)^3^,^19^. Second, both the BcII and VIM-2 structures reveal that in the MBL-active sites the bicyclic phenyl-boronate ring of **2** is very similarly positioned to the cephalosporin dihydrothiazine ring (or analogous penicillin/carbapenem derived rings)^19^, with both being positioned to make hydrophobic interactions with the conserved Trp_87_ and Phe_61_ residues. Notably, the ‘endocyclic' boronate ester oxygen of **2** coordinates to Zn2, mimicking the coordination of the cephalosporin-derived dihydrothiazine ring nitrogen in the anionic intermediate. Third, the binding mode of the side chain of **2** is analogous to that of the 7β side chain of cephalosporins (Fig. 2b,d), in that the carbonyl oxygen of the 7β-acetamido group of the side chain of **2** and the cephalosporin intermediate are both positioned to hydrogen bond with the main chain NH group of residue-119 (Ala (BcII), Asp (VIM-2) or Glu (NDM-1)). Fourth, as observed in a complex of NDM-1 with a cephalosporin-derived intermediate, the C-6 carboxylate arising from β-lactam hydrolysis is positioned to coordinate Zn1 and hydrogen bond via one of its oxygen atoms to the Asn_233_ side chain. Fifth, binding of the two ‘exocyclic' boron-bound oxygens/hydroxides mimics the binding modes proposed for the two oxygens in the oxyanion intermediate. The *pro*-(*S*) boron-bound exocyclic oxygen coordinates with Zn1 and is positioned to hydrogen bond with Asn_233_ and the NH of the acetamido side chain. In MBL catalysis, the highly conserved Asn_233_ side chain is proposed to stabilize the oxyanionic intermediate via hydrogen bonding with the lactam carbonyl-derived oxygen. 20 (The structures suggest that hydrogen bonding to the substrate side chain is also involved in stabilizing the oxyanion). The *pro*-(*R*) boron-bound exocyclic oxygen bridges Zn1 and Asp_120_, mimicking the proposed position of the hydroxide-derived oxygen in the oxyanion intermediate. Overall, the complexes of **2** with VIM-2 and BcII reveal that binding of the cyclic boronates closely mimics that proposed for the high-energy tetrahedral intermediate in MBL catalysis^21^ (Fig. 2a,e), both in terms of structure and bonding.

### Structural analysis of cyclic boronate binding to OXA-10

**Figure 3:**
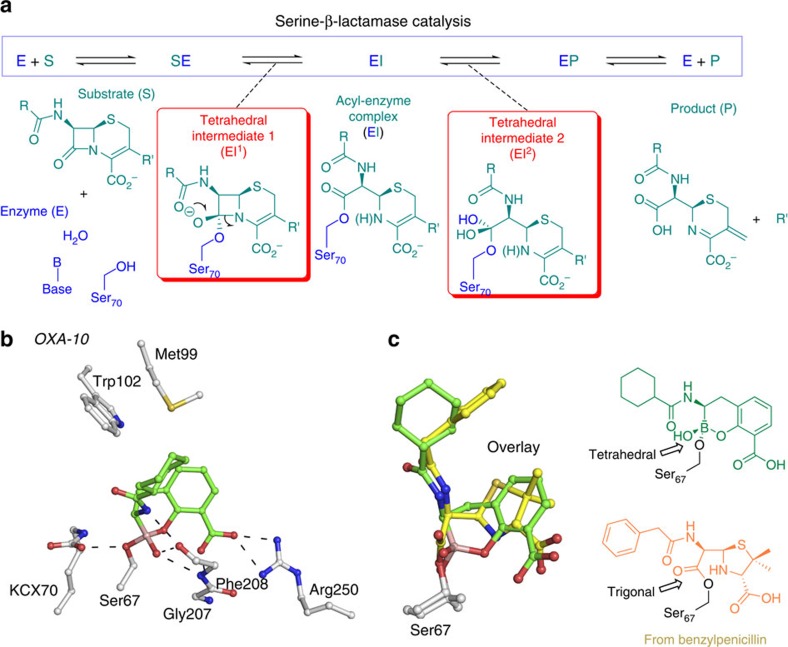
Mode of action of serine-β-lactamases and binding mode of cyclic boronates. (**a**) Outline mode of action of SBLs. (**b**) View from a structure obtained by co-crystallization of **1** with OXA-10 (Chain A) revealing the binding mode of cyclic boronates to SBLs. (**c**) The overlay compares the binding modes of **1** and hydrolysed benzylpenicillin in complex with OXA-10 (PDB ID: 2WGI).

We then investigated the binding mode of the cyclic boronates to SBLs, by determining a high-resolution crystal structure of the Class D β-lactamase OXA-10 in complex with **1** (1.5 Å resolution; Supplementary Fig. 3,7). OXA-10 requires a carbamylated active site lysine (KCX70) for catalysis^22^ (Fig. 3a), which was observed in the OXA-10:**1** complex structure (Fig. 3b, Supplementary Fig. 3). Inspection of maps (Supplementary Fig. 3) reveals continuous electron density connecting the inhibitor boron atom with the terminal hydroxyl group of the nucleophilic serine (Ser_67_) and provides clear evidence for a tetrahedral boron atom, contrasting with the planar geometry of the carbonyl carbon atoms of β-lactam acyl-enzyme complexes (Supplementary Fig.8). The structure of the complex with **1** thus represents a covalent species that better resembles the ‘first' tetrahedral intermediate, which is involved in acyl-enzyme formation, rather than the acyl-enzyme itself (Fig. 3a). Hence, analogous to our structural observations with the VIM-2 and BcII MBLs (above), the OXA-10:**1** complex structure reveals that **1** mimics the mechanistically important tetrahedral oxyanion that is transiently present on the hydrolytic pathway. Furthermore, comparison of the OXA-10:**1** complex with that of a penicillin (benzylpenicillin) bound to a ‘deacylation-deficient' OXA-10 (K70C variant)^23^ reveals strikingly related overall binding modes. In particular, the carboxylate of **1** and the C-3 carboxylate of penicillin (Fig. 3b,c) are both positioned to interact with Arg_250_, while the acetamido group of **1** and the benzylpenicillin C6 amide group are analogously positioned to hydrogen bond with the main chain carbonyl of Phe_208_. Finally, the endocyclic boronate ester oxygen of **1** and the β-lactam-derived nitrogen (from the penicillin thiazolidine ring) are both positioned to hydrogen bond with Ser_115_.

### Structural analysis of cyclic boronate binding to PBP 5

**Figure 4:**
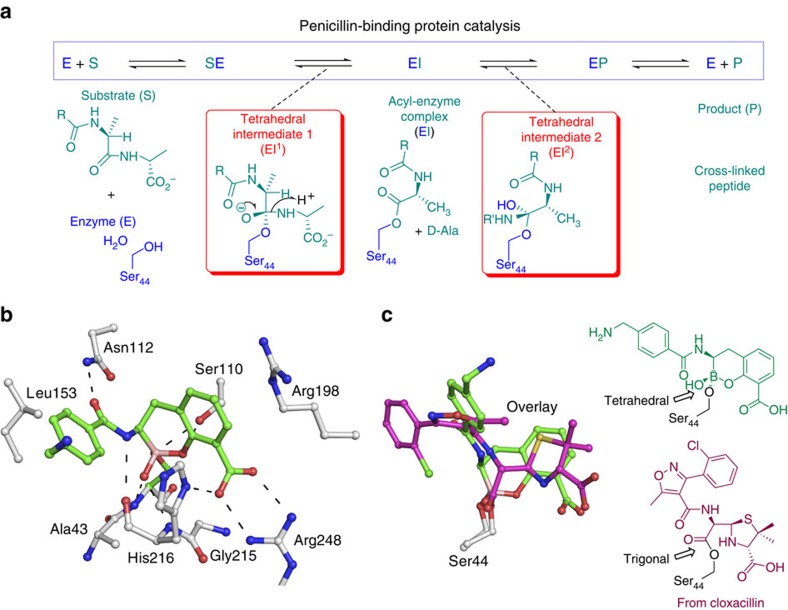
Mode of action of penicillin-binding proteins and binding mode of cyclic boronates. (**a**) Outline mode of action of PBPs. (**b**) View from a structure obtained by co-crystallization of **2** with PBP 5 reveals the binding of cyclic boronates to PBPs. (**c**) The overlay compares the binding modes of **2** and hydrolysed cloxacillin in complex with PBP 5 (PDB ID: 3MZD).

We then carried out crystallographic studies to investigate the binding mode of the cyclic boronates to PBPs (Fig. 4a), by solving a crystal structure of PBP 5 in complex with **2** (2.5 Å resolution; Supplementary Fig. 3,9). Comparison of the PBP 5:**2** complex structure with reported PBP5 structures reveals that the fold of Domain 1 (aa 3–262) of PBP 5, which contains the transpeptidase/penicillin-binding active site, is very similar in both structures (Supplementary Fig. 10); However, clear differences are observed in the position of Domain 2 (aa 263–356).

To date, all reported PBP 5 structures with ligands (substrate-like peptide boronic acids or acyl-enzyme complexes with penicillins, cephalosporins or carbapenems) manifest weak or absent density for the C6 or C7 side chains of the penicillins or cephalosporins, respectively, or the C3′ cephalosporin side chain^24^,^25^,^26^. Importantly, for the PBP 5:**2** complex, inspection of the experimental map revealed clear electron density for the whole inhibitor molecule (Supplementary Fig. 3).

Comparison of the PBP 5:**2** complex with a PBP 5:cloxacillin acyl-enzyme structure^25^ reveals similar binding modes for the two compounds in the active site (Fig. 4b,c), including with respect to (i) interactions with the conserved active site motifs (SxxK and SxN), the conformations of which overlap almost exactly in the two structures; (ii) conservation of the predicted active site hydrogen-bonding network; and (iii) the same bidendate interaction of Arg248 with the carboxylate of **2** or the C-3 penicillin carboxylates^25^.

Three other features of the PBP 5:**2** complex structure are of interest. First, the conserved Ω-like loop that is conserved in PBPs and Class A SBLs adopts a ‘closed' position (that is, closer to the active site) when complexed with **2**, compared with previously published apo- or acyl-enzyme structures (Supplementary Fig. 11). Interestingly, in the structure of PBP 5 complexed with substrate-like peptide boronic acid, the Ω-like loop was refined in dual conformations, one similar to that observed in the PBP5:**2** structure and one similar to the apo- and the acyl-enzyme structures (Supplementary Fig. 11). Second, compared with the previous acyl-enzyme structures, in the structure with **2**, His216 moves >3.5 Å to interact with the carboxylate of **2**, which is analogous to binding of the penicillin C-3 carboxylate, implying an important role for this residue in substrate binding. Third, the ‘bridging' water molecule that is proposed to polarize and orient the hydrolytic water molecule for hydrolysis in the acyl-enzyme complex was absent in the PBP 5:2 structure (Supplementary Fig. 12).

## Discussion

The combined results reveal that cyclic boronates act as dual action inhibitors of SBLs and MBLs by mimicking a common high-energy tetrahedral intermediate; they also reveal that cyclic boronates can potently inhibit some PBPs via the same mechanism. Cyclic boronates thus represent a promising line of investigation not only for the protection of BLAs from both MBLs and/or SBLs, but also for direct inhibition of PBPs. The structural results presented here will aid in the development of optimized cyclic boronates, possibly with activity against the essential PBPs.

The demonstrated utility of a single compound (class) to simultaneously inhibit both nucleophilic serine- and metallo-hydrolases is also of interest in other drug discovery fields, including cancer, where it may be desirable to inhibit different classes of functionally related proteases^27^. Notably, the cyclic boronates, but not acyclic boronic acids, were potent MBL inhibitors, suggesting that cyclic, or otherwise conformationally constrained transition state analogues, either employing boronates or related functional groups that mimic intermediates (for example, fluoromethyl ketones) may represent a generally productive route towards simultaneous inhibition of mechanistically different types of hydrolases by the same compound.

## Methods

### Synthesis

the procedures for the synthesis of cyclic boronates are described in Supplementary Methods. For NMR and MS analysis of the compounds in this article, see Supplementary Figs 13–59.

### Protein production and purification

Recombinant forms of NDM-1, NDM-1 M67C, VIM-2, SPM-1, IMP-1, BcII, CphA, Tem-1, OXA-10, PBP 3 and PBP 5 were produced in *E. coli* as described^9^,^10^,^23^,^28^,^29^,^30^. Purified BcII, VIM-2, OXA-10 and PBP 5 proteins were separately dialysed into fresh crystallization buffer (50 mM HEPES pH 7.5, 150 mM NaCl and 1 μg ZnCl_2_ for BcII and VIM-2 or 50 mM MES pH 6.0, 100 mM NaCl for OXA-10 or 50 mM TRIS pH 7.5 and 500 mM NaCl for PBP 5).

### Crystallography

Crystals of BcII:**2**, VIM-2:**2**, OXA-10:**1** and PBP5:**2** were grown using the conditions stated in Supplementary Table 2. Crystals were cryoprotected using well solution diluted to 25% glycerol and harvested using nylon loops then flash-cooled and stored in liquid nitrogen. Diffraction data for VIM-2:**2** and OXA-10:**1** crystals were collected at the Diamond Light Source synchrotron beamline I04, data for BcII:**2** were collected using a Rigaku FRE+ Superbright diffractometer and data for PBP5:**2** were collected at the Diamond Light Source synchrotron beamline I02. All data sets were collected at 100 K. Data for BcII, VIM-2 and PBP 5 were indexed, integrated and scaled using HKL-2000 and for OXA-10 with Mosflm and Scala, respectively^31^. All structures were solved by molecular replacement using Phaser^32^ using the previosly published VIM-2 (PDB ID: 4BZ3)^17^, OXA-10 (PDB ID: 1K6R)^22^, BcII (PDB ID: 4C09)^17^ or PBP 5 (PDB ID: 1NZO) as search models for their respective data set. The structures were then iteratively fit and refined using COOT^33^ and PHENIX^31^ until *R*_work_ and *R*_free_ no longer converged. Data collection and refinement statistics are given in Supplementary Table 3.

### Kinetic analyses

Kinetic and inhibition analyses, and ^19^F NMR studies^10^ against bacterial β-lactamases were performed as described^9^. The FC-5 cephalosporin^9^ was used as substrate for BcII, VIM-2, IMP-1, NDM-1, SPM-1, TEM-1 and OXA-10, meropenem^28^ for CphA and nitrocefin^34^ for PBP 3 and PBP 5.

In brief IC_50_ values were determined by preincubation of the appropriate amount of enzyme with the cyclic boronates in the assay buffers for 10 min at room temperature prior to the initiation of the assay by the addition of the appropriate substrate. The enzyme concentrations used are the following: 1 nM BcII, 100 nM VIM-2, 10 nM IMP-1, 10 nM NDM-1, 1 nM SPM-1, 1 nM TEM-1, 1 nM OXA-10, 2.5 nM CphA, 4 μM PBP 3 and 5 μM PBP 5; note that the final concentrations are stated. The assay buffers used are the following: 50 mM HEPES, pH 7.2 supplemented with 1 μg BSA, 1 μg ZnSO_4_ and 0.01% Triton for BcII, VIM-2, IMP-1, NDM-1 and SPM-1, 15 mM MES, pH 6.5 for CphA, 50 mM HEPES, pH 7.2 supplemented with 200 mM NaCl and 0.01% Triton 100 for TEM-1, 100 mM sodium phosphate, pH 7.2 supplemented with 50 mM NaHCO_3_ and 0.01% Triton 100 for OXA-10 and 50 mM HEPES, pH 7.2 supplemented with 200 mM NaCl and 0.01% Triton 100 for PBP 3 and PBP 5. The amount of substrates used is the following: 5 μM for BcII, VIM-2, IMP-1, NDM-1 and SPM-1, 10 μM for TEM-1 and OXA-10, 125 μM for CphA and 25 μM for PBP 3 and PBP 5; note that the final concentrations are stated.

The experiments were performed using a PHERAstar FS microplate reader (BMG Labtech) at room temperature (24–25 °C). All enzymes and inhibitors were prepared in the specific assay buffer. The kinetic values in this study are the means from at least three independent measurements; at least nine different concentrations of the inhibitor were used to determine the kinetic parameters (IC_50_). The IC_50_ values were determined using the software package Graph Prism 5.

### Cytotoxicity assay

HEK293 cells were seeded (2,000 per well) in normal DMEM in 96-well plates.^34^ After 24 h, wells were dosed with titrated concentration of compounds in 1% dimethyl sulphoxide (DMSO)—final volume of media about 100 μl. Following a further 24 h incubation period, 20 μl tetrazolium (MTS, CellTiter96Aqueous One Solution (Promega)) was added to the media in each well. The plate was incubated at 37 °C, 5% CO_2_ for 4 h in the dark before absorbance was read at 490 nm to determine cell proliferation values.

### Microbiological susceptibility testing

Bacterial strains: *E. coli* ATCC 25922 (ref. 35) and *K. pneumoniae* NCTC 5055 (ref. 36) were used as reference strains. *K. pneumoniae* Ecl8 (ref. 16) and its *ramR* knock-out^37^ were used as previously described^38^,^39^. *K. pneumoniae* strain IR8 (ref. 40), and *E. coli* strains IR10 and IR60 (ref. 17) were generous gifts of Prof. T.R. Walsh. Plasmid pSU18:NDM-1 contains the complete *bla*_NDM-1_ open reading frame, together with its promoter, cloned into plasmid pSU18 (ref. 41). The recombinant plasmid was made by PCR amplifying the *bla*_NDM-1_ gene and surrounding sequences from *E. coli* IR10 with primers NDM1F 5′- ACACCATTAGAGAAATTTGC -3′ and NDM1R 5′- GGCGATGACAGCATCATC -3′, TA cloning the amplicon into pCR2.1 TOPO (Invitrogen) and subcloning into pSU18 using BamHI and XbaI.

MIC values were determined by broth microdilution, in triplicate, in cation-adjusted Mueller Hinton broth (Sigma) according to the Clinical Laboratory Standards Institute (CLSI) guidelines^42^. Experiments were carried out in microtitre plates (Corning) containing the medium plus meropenem (Sequoia Research Products, Pangbourne, UK) and/or inhibitor **2** as appropriate. Chloramphenicol (20 μg ml^−1^) was included in assays of strains (*K. pneumoniae* Ecl8 and derivative) that carried pSU18 plasmids or derivatives (pSU18:NDM-1) to ensure maintenance of plasmids throughout. Inhibitor **2** was dissolved in DMSO to 100 mg ml^−1^ and diluted in broth. The final concentration of DMSO in the assays was kept constant at 0.01% (experiments using 10 μg ml^−1^ inhibitor) or 0.03% (experiments using 25 μg ml^−1^ inhibitor). Plates were incubated overnight at 37 °C for 18–24 h and absorbance at 600 nm read using a Polarstar Omega (BMG LabTech) plate reader.

### Data availability

Data supporting the findings of this study are available within the article (and its Supplementary Information files) and from the corresponding author upon reasonable request. Coordinates and structure factors have been deposited in the Protein Data Bank under accession codes 5FQB (BcII-**2**), 5FQC (VIM-2:**2**), 5FQ9 (OXA-10:**1**) and 5J8X (PBP 5:**2**).

## Additional information

**Accession codes:** Coordinates and structure factors have been deposited in the Protein Data Bank under accession codes 5FQB (BcII:**2**), 5FQC (VIM-2:**2**), 5FQ9 (OXA-10:**1**) and 5J8X (PBP 5:**2)**.

**How to cite this article:** Brem, J. *et al*. Structural basis of metallo-β-lactamase, serine-β-lactamase and penicillin-binding protein inhibition by cyclic boronates. *Nat. Commun.* 7:12406 doi: 10.1038/ncomms12406 (2016).

## Supplementary Material

Supplementary Information Supplementary Figures 1-59, Supplementary Tables 1-3, Supplementary Methods and Supplementary References.

## Figures and Tables

**Table 1:** *In vitro* screening of cyclic boronates.

	**IC**_**50**_ **(μM)/pIC**_**50**_**/s.e. logIC**_50_*				
	**1**	**2**	**3**	**4**	**5**
BcII	7.27/5.1/0.052	0.30/6.5/0.032	0.96/6.0/0.041	0.52/6.2/0.194	1.14/5.9/0.029
VIM-2	0.051/7.3/0.034	0.003/8.5/0.058	0.011/7.9/0.028	0.014/7.8/0.044	0.002/8.7/0.1144
IMP-1	1.44/4.6/0.116	1.00/5.0/0.089	1.50/4.5/0.112	1.21/4.7/0.127	1.41/4.5/0.127
NDM-1	2.04/5.7/0.028	0.029/7.5/0.020	0.687/6.1/0.068	0.04/7.4/0.021	0.004/7.4/0.051
SPM-1	24.1/4.6/0.162	16.7/4.7/0.103	16.0/4.8/0.093	13.9/4.8/0.172	36.3/4.4/0.207
CphA	4.42/5.3/0.159	>100/>4/-	20.85/4.68/0.23	>100/>4/-	>100/>4/-
TEM-1	0.001/8.9/0.447	0.003/8.4/0.033	0.002/8.5/0.030	0.0003/9.4/0.46	0.006/8.1/0.007
OXA-10	0.33/6.4/0.019	5.1/5.2/0.020	0.83/6.0/0.012	2.26/5.6/0.028	12.7/4.9/0.0258
PBP 5	NT	0.0016/8.7/0.06	NT	NT	NT
PBP 3	NI	NI	NI	NI	NI

MBL, metallo-β-lactamase; NI, no inhibition at 100 μM; NT, not tested; PBP, penicillin-binding protein; SBL, serine-β-lactamase.

*Errors were calculated as s.d. of at least three independent measurements. Note compounds **2, 4** and **5** were identified in the patent literature^8^.

IC_50_/pIC_50_ values of cyclic boronates against a panel of MBLs, SBLs and PBPs (see Supplementary Methods for experimental details).

**Table 2:** *In vitro* cell-based screening of cyclic boronate 2.

**Bacterial strain**	**Genotype (β-lactamases)**	**2 MIC (μg ml**^−**1**^)	**Meropenem MIC (μg ml**^−**1**^)	**Meropenem MIC (μg ml**^−**1**^**) (10 μg ml^−**1**^ 2)**	**Meropenem MIC (μg ml**^−**1**^**) (25 μg ml^−**1**^ 2)**
*K. pneumoniae* Ecl8 pSU18	*bla*_*SHV-1*_	>128	≤0.25	≤0.25	≤0.25
*K pneumoniae* Ecl8 ΔramR pSU18	*bla*_*SHV-1*_	>128	≤0.25	≤0.25	≤0.25
*K. pneumoniae* Ecl8 pSU18:NDM-1	*bla*_*SHV-1*_; *bla*_*NDM- 1*_	>128	>128	8	4
*K pneumoniae* Ecl8 ΔramR pSU18:NDM-1	*bla*_*SHV-1*_; *bla*_*NDM- 1*_	>128	>128	16	2
*E. coli* IR10	*bla*_*DHA-1*_; *bla*_*CTX-M-15*_; *bla*_*TEM-1*_; *bla*_*OXA-1*_; *bla*_*NDM- 1*_	>128	128	8	2
*E. coli* IR60	*bla*_*DHA-1*_; *bla*_*CTX-M-15*_; *bla*_*TEM-1*_; *bla*_*OXA-1*_; *bla*_*NDM- 1*_	>128	64	16	16
*K. pneumoniae* IR8	*bla*_*SHV-1*_; *bla*_*DHA-1*_; *bla*_*CTX-M-15*_; *bla*_*TEM-1*_; *bla*_*NDM-1*_	>128	128	32	16

MIC, minimal inhibitory concentration.

Testing the potency of **2** towards resenzitization of meropenem activity using well-characterized and clinically derived strains.
