# Supplementary material for: Structural basis of metallo-β-lactamase, serine-β-lactamase and penicillin-binding protein inhibition by cyclic boronates
Source: Nat Commun. 2016 Aug 8;7:12406. doi: 10.1038/ncomms12406 (PMC4979060; doi:10.1038/ncomms12406)
Supplement: Supplementary Information — Supplementary Figures 1-59, Supplementary Tables 1-3, Supplementary Methods and Supplementary References. [file ncomms12406-s1.pdf]

Supplementary Figures

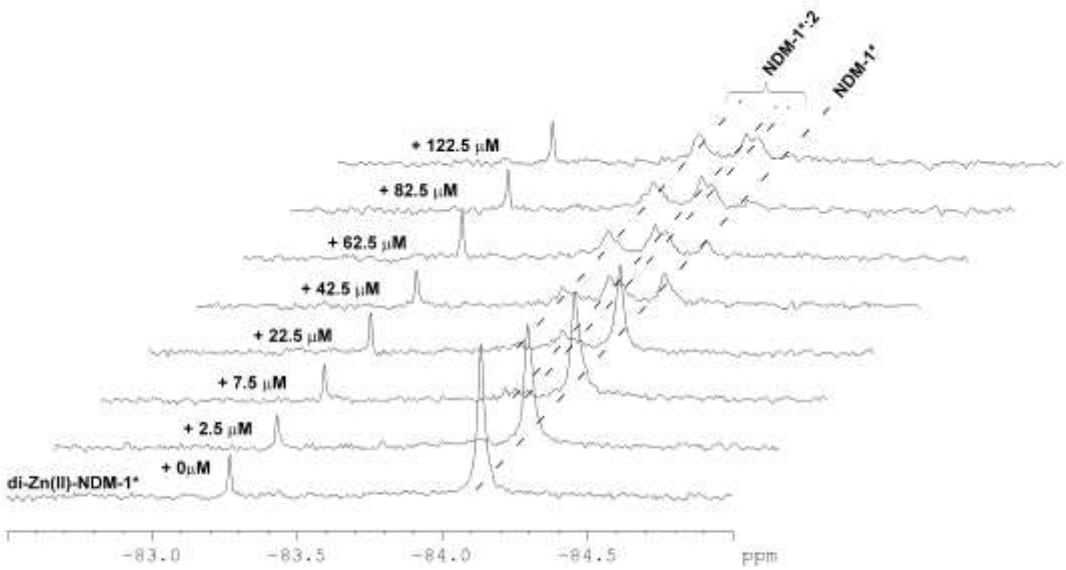

Supplementary Fig. 1. ^{19}F NMR analyses of the NDM-1*-di-Zn(II)-**2** complex. Titration using **2** gave rise to a new signal corresponding to a **2**-NDM-1* complex, even at low concentrations of **2** indicating tight binding; at a 1:1, enzyme:inhibitor ratio (NDM-1*:**2**) no signals corresponding to uncomplexed NDM-1* species were observed. NDM-1* = NDM-1 M67C variant modified by reaction with $BrCH_2COCF_3$[1].

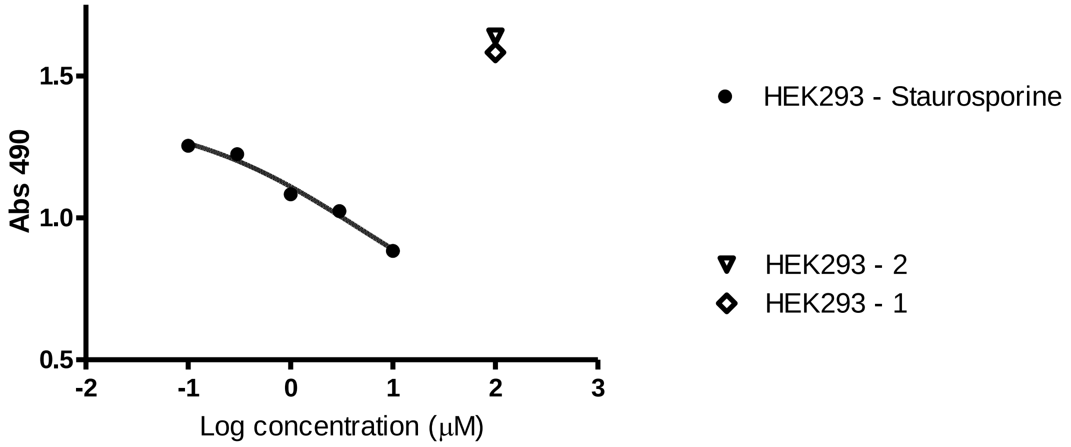

Supplementary Fig. 2. Cytotoxicity of **1**,**2** and Staurosporine (a control) using HEK293 cells.

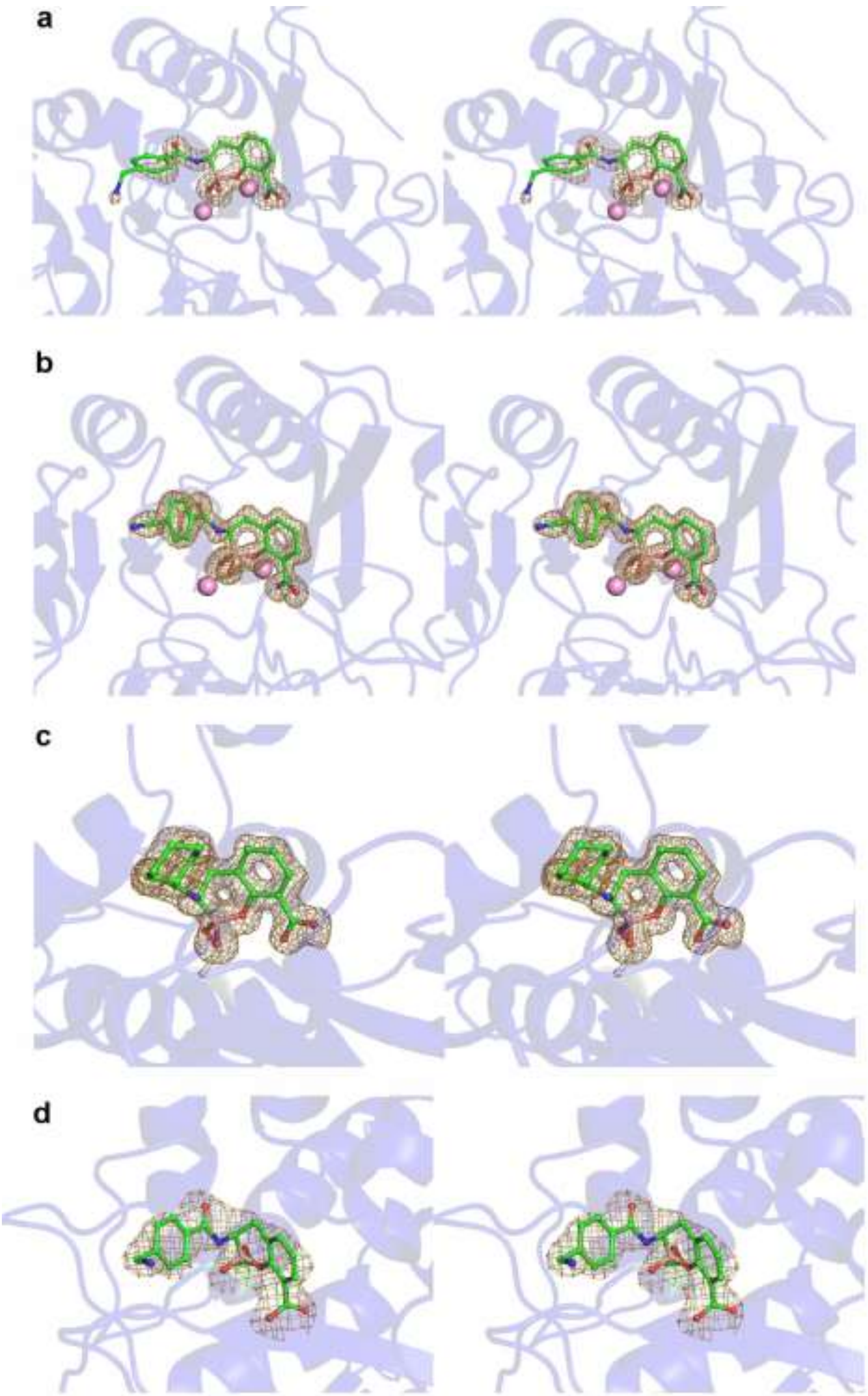

Supplementary Fig. 3. Stereoimage from (a) BcII:**2** (PDB ID: 5FQB), (b) VIM-2:**2** (PDB ID: 5FQC), (c) OXA-10:**1** (PDB ID: 5FQ9) and (d) PBP 5:**2** (PDB ID: 5J8X) complex structures (Chain A in all cases) with the *Fo-Fc* OMIT density map contoured to 3σ (brown mesh). Note, that for the BcII structure the inhibitor **2** was modelled and refined at 70% occupancy; for VIM-2, OXA-10, PBP 5 the inhibitors **2, 1** and **2**, respectively, were modelled and refined at full occupancy.

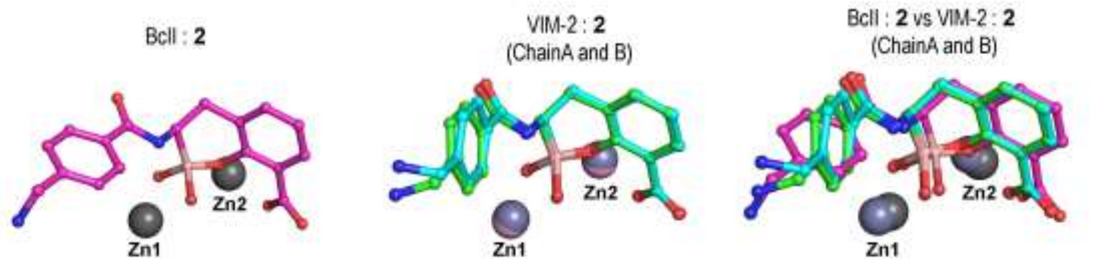

Supplementary Fig. 4. Views from crystal structures of BcII (PDB ID: 5FQB) and VIM-2 (PDB ID: 5FQC) complexed with **2**. Note, that for VIM-2, **2** was modelled in Chains A and B.

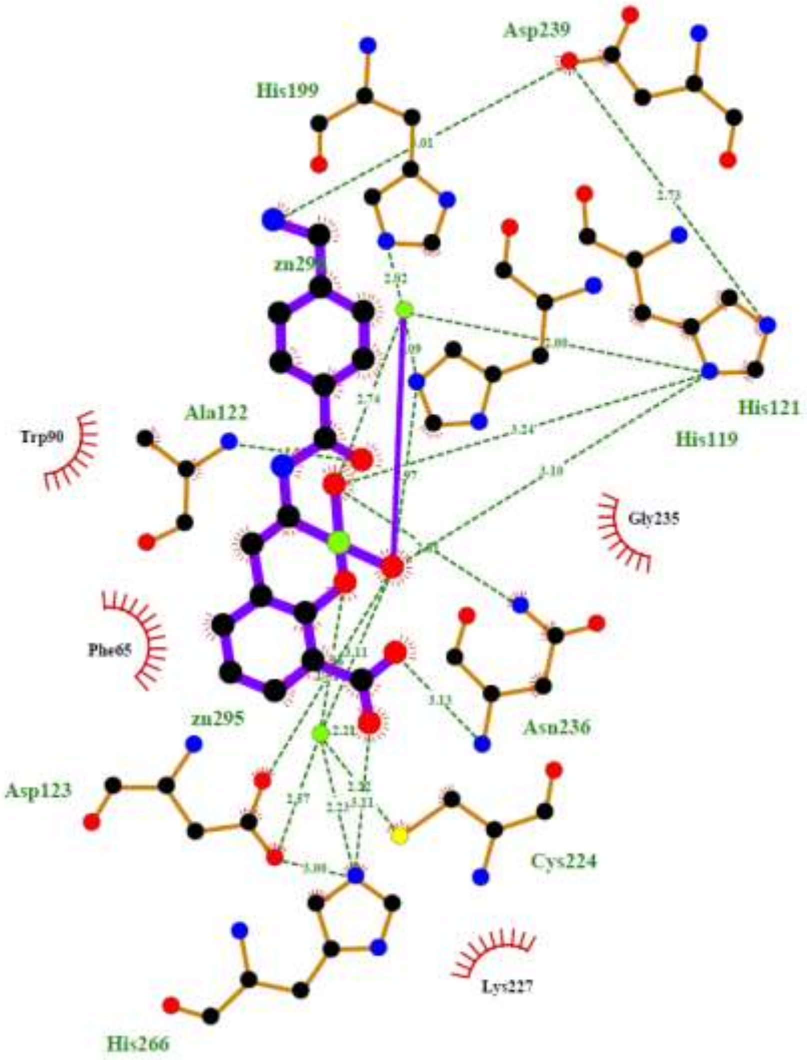

Supplementary Fig. 5. View of the protein-ligand interactions derived from a structure obtained by co-crystallization of 2 with BcII (PDB ID: 5FQB). Protein-ligand interactions between BcII and **2** depicted using LIGPLOT[2]. Hydrogen bonding interactions are shown as green dashed lines and ligand-protein hydrophobic contacts are shown as large red curved combs.

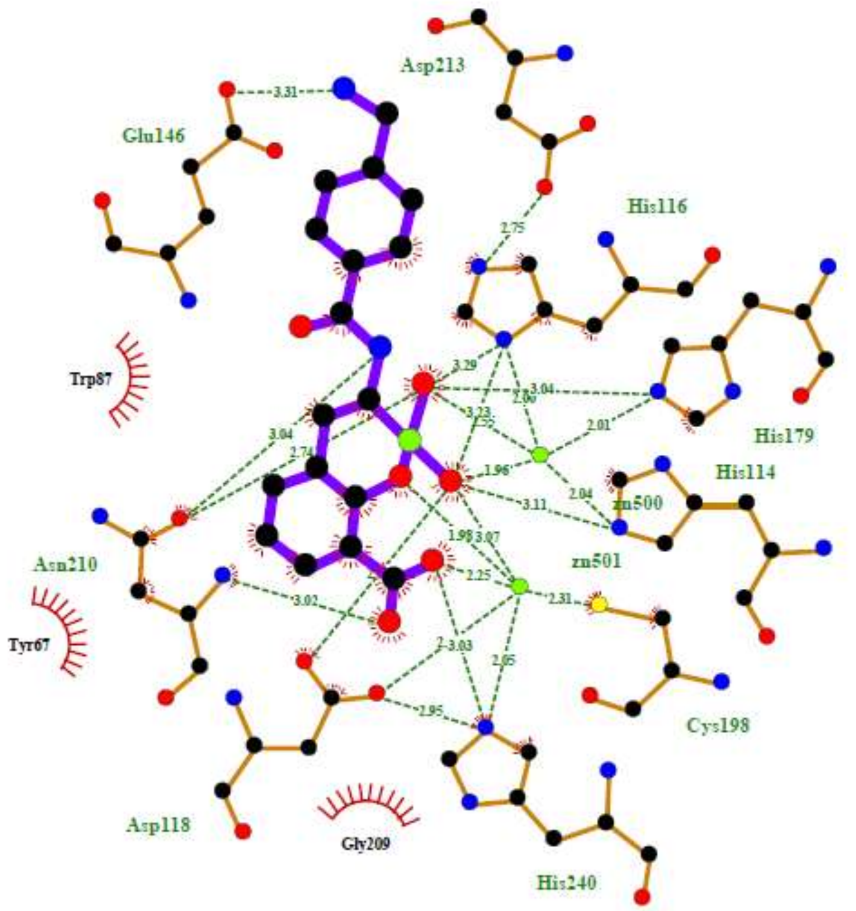

Supplementary Fig. 6. View of the protein-ligand interactions derived from a structure obtained by co-crystallization of 2 with VIM-2 (PDB ID: 5FQC). Protein-ligand interactions between VIM-2 and **2** depicted as observed in monomer A using LIGPLOT[2]. Hydrogen bonding interactions are shown as green dashed lines and ligand-protein hydrophobic contacts are shown as large red curved combs.

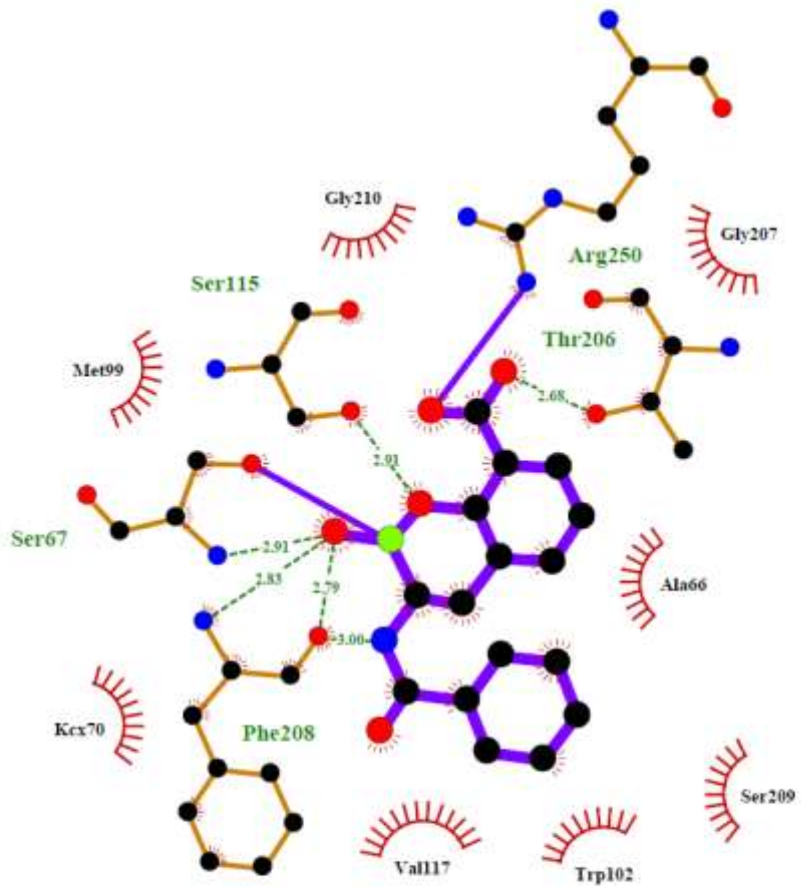

Supplementary Fig. 7. View of the protein-ligand interactions derived from a structure obtained by co-crystallization of 1 with OXA-10 (PDB ID: 5FQ9). Protein-ligand interactions between OXA-10 and **1** depicted as observed in monomer A using LIGPLOT[2]. Hydrogen bonding interactions are shown as green dashed lines and ligand-protein hydrophobic contacts are shown as red curved combs.

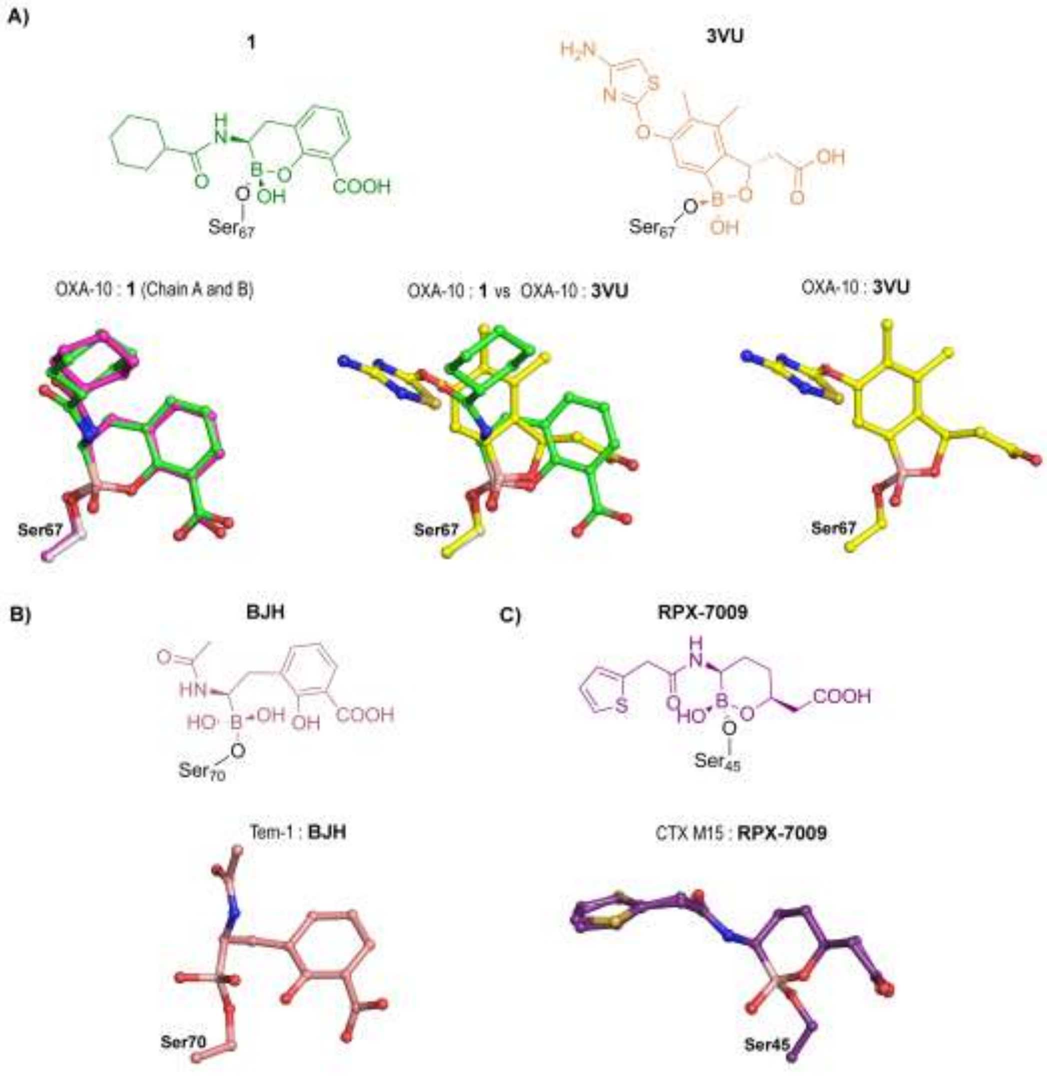

Supplementary Fig. 8. Views from crystal structures of serine β-lactamases with various boronic acids (OXA-10:**1** complex structure PDB ID: 5FQ9; OXA-10:**3VU** complex structure PDB ID: 4WZ5[3]; TEM-1:**BJH** complex structure PDB ID: 1ERQ[4], and a CTX M-15:**RPX-7009** complex structure PDB ID: 4XUZ[5]).

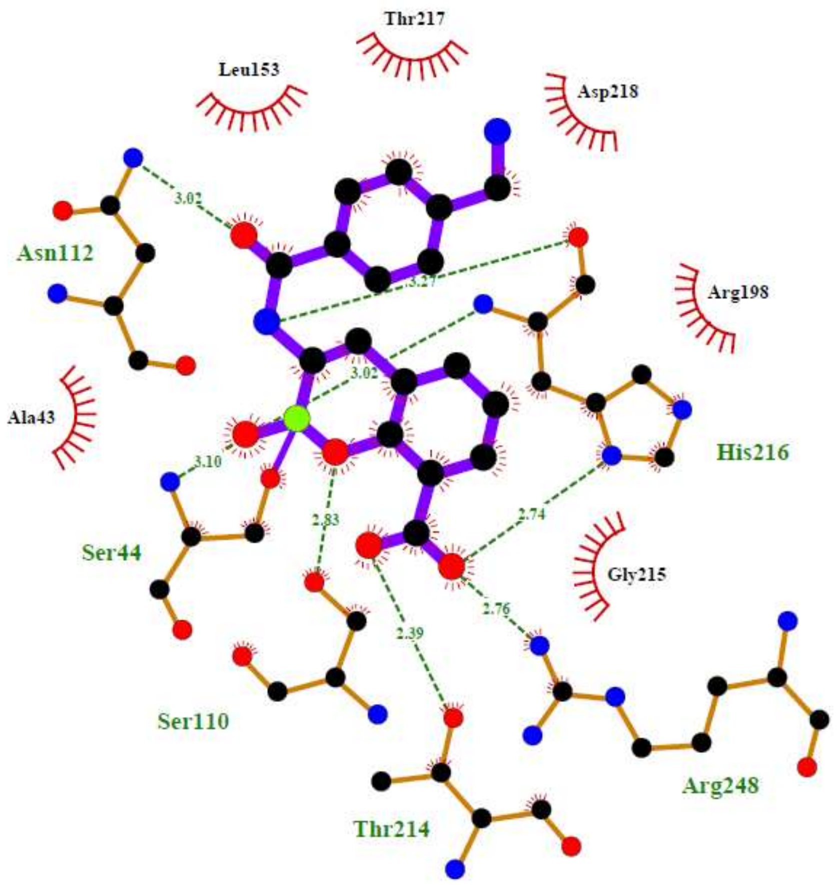

Supplementary Fig. 9. View of the protein-ligand interactions derived from a structure obtained by co-crystallization of 2 with PBP5 (PDB ID: 5J8X). Protein-ligand interactions between PBP 5 and **2** depicted using LIGPLOT[2]. Hydrogen bonding interactions are shown as green dashed lines and ligand-protein hydrophobic contacts are shown as red curved combs.

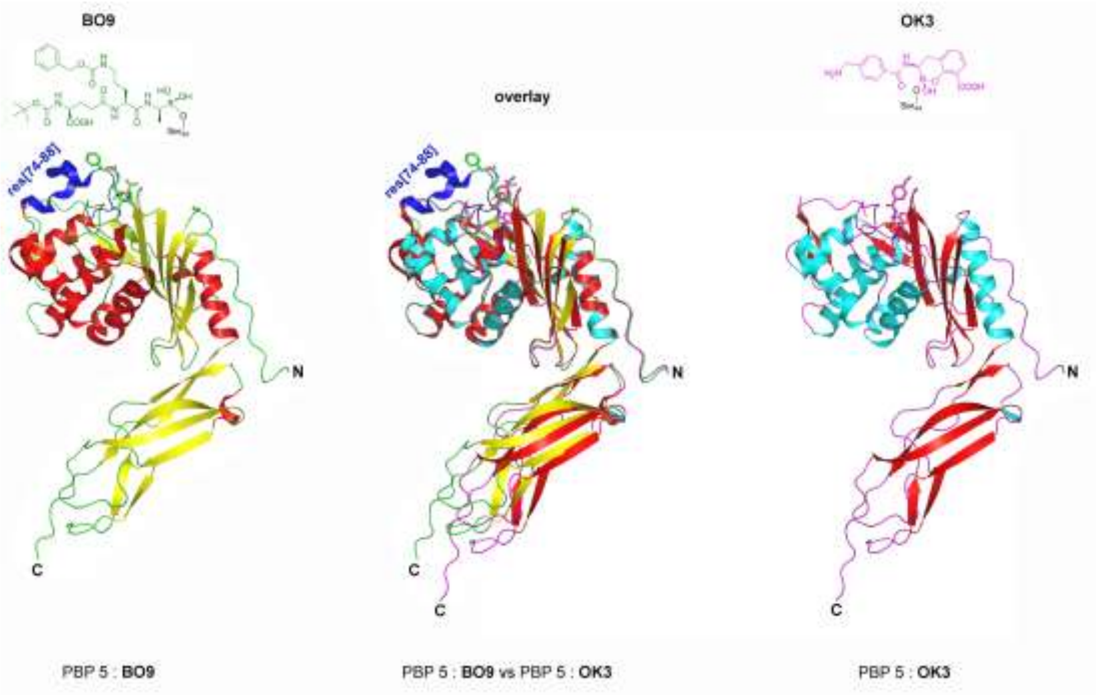

Supplementary Fig. 10. Views from crystal structures of penicillin binding protein 5 (dacA) from *E. coli* complexed with various boronic acids; Right, a reported structure of PBP 5 complexed with a tripeptide boronic acid[6] (PBP 5: **BO9** complex structure, PDB ID: 1Z6F); Left, structure of PBP 5 complexed with cyclic boronates – reported in this paper (PBP 5:**2** complex structure, PDB ID: 5J8X); Centre, overlay of the two structures. Note, (a) the distinct conformation of the domain 2, and (b) the unmodelled residues 74-88 (disordered region) in the case of the PBP 5:**2** complex structure.

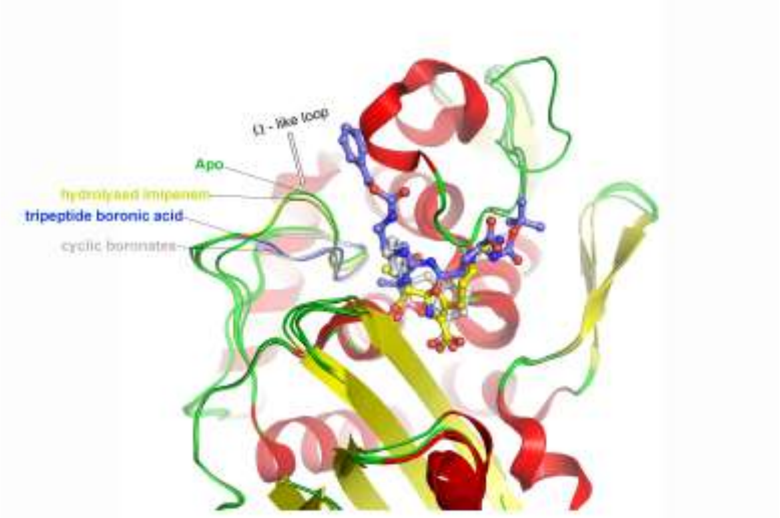

Supplementary Fig. 11. Views from crystal structures of penicillin binding protein 5 (dacA) from *E. coli* in uncomplexed form[7] (PDB ID: 1NJ4) and complexed with various inhibitors (PBP 5 with hydrolysed imipenem[8], PDB ID: 3MZF; PBP 5 with a tripeptide boronic acid[6], PDB ID: 1Z6F and PBP 5 with cyclic boronate **2** PDB ID: 5J8X). Note that in the active site the two different conformations of the Ω-like loop.

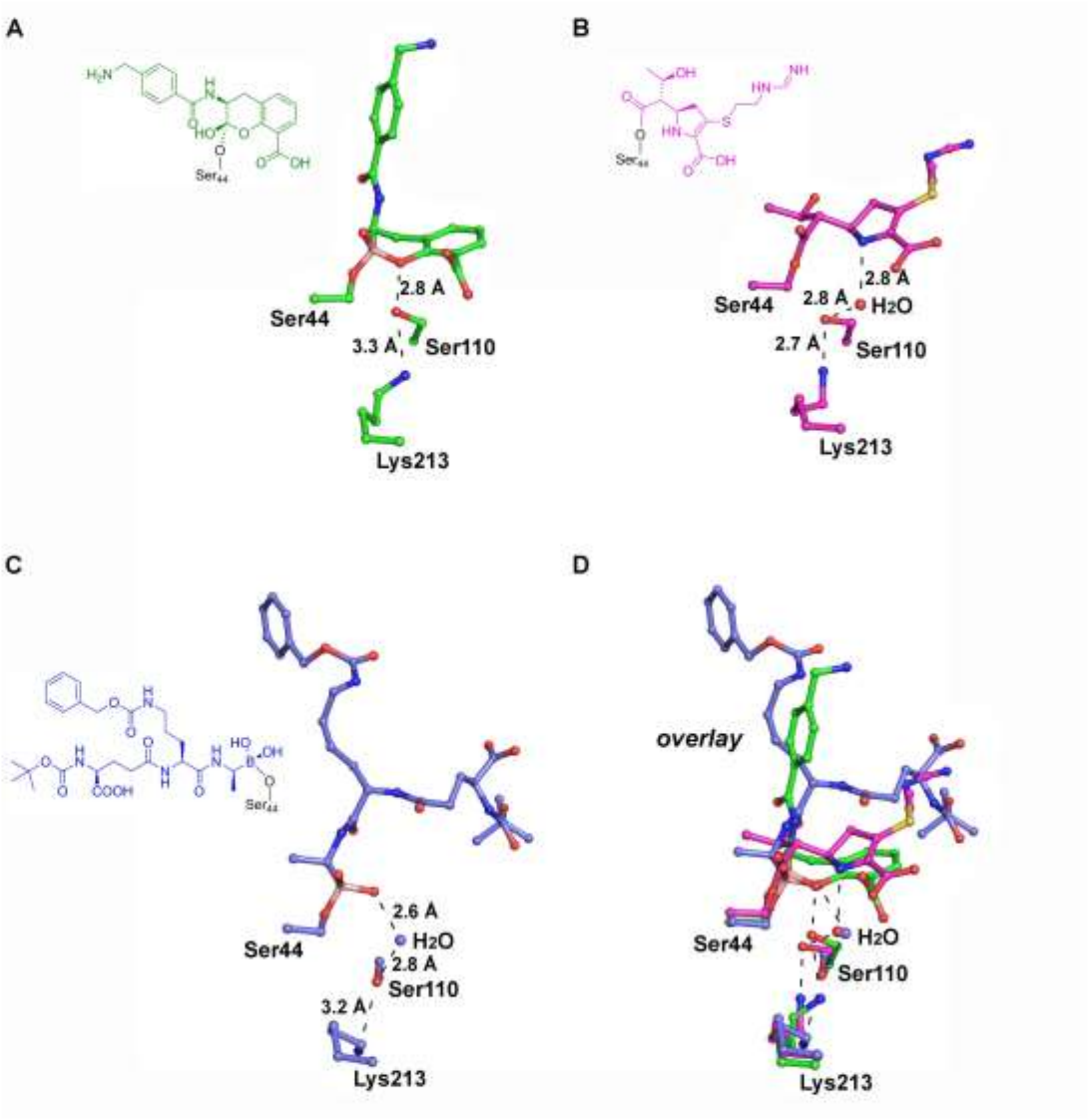

Supplementary Fig. 12. Views from crystal structures of penicillin binding protein 5 from *E. coli* (PBP 5) with various inhibitors (PBP 5 with cyclic boronate PDB ID: 5J8X; PBP 5 with hydrolysed imipenem[8], PDB ID: 3MZF and PBP 5 with the tripeptide boronic acid[6], PDB ID: 1Z6F). Note that in the case of the cyclic boronate co-crystal structure the "bridging" water molecule that is proposed to polarise and orient a water molecule for hydrolysis of the acyl-enzyme complex is not observed.

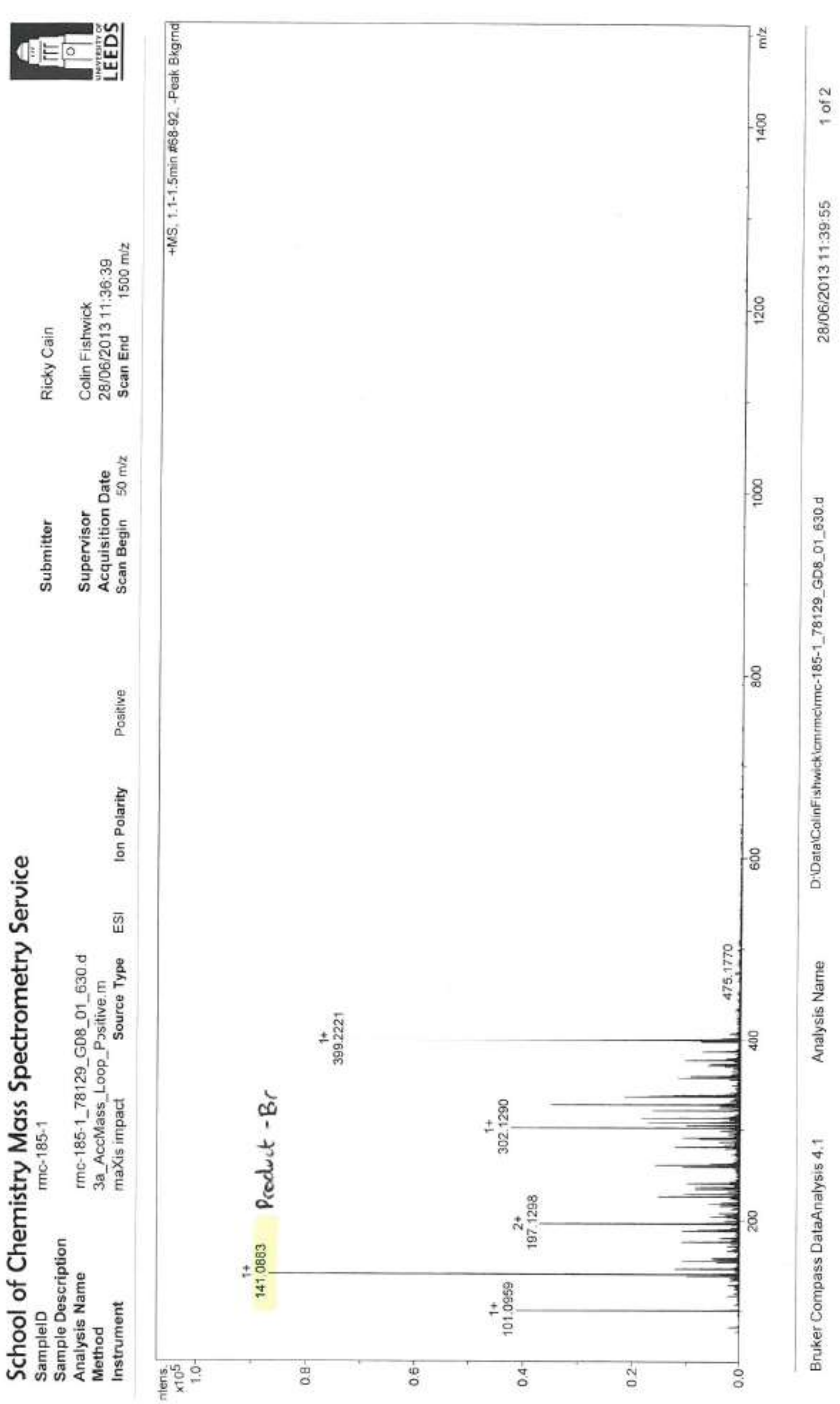

Supplementary Fig. 13. MS spectrum of S2.

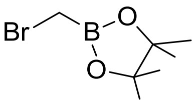

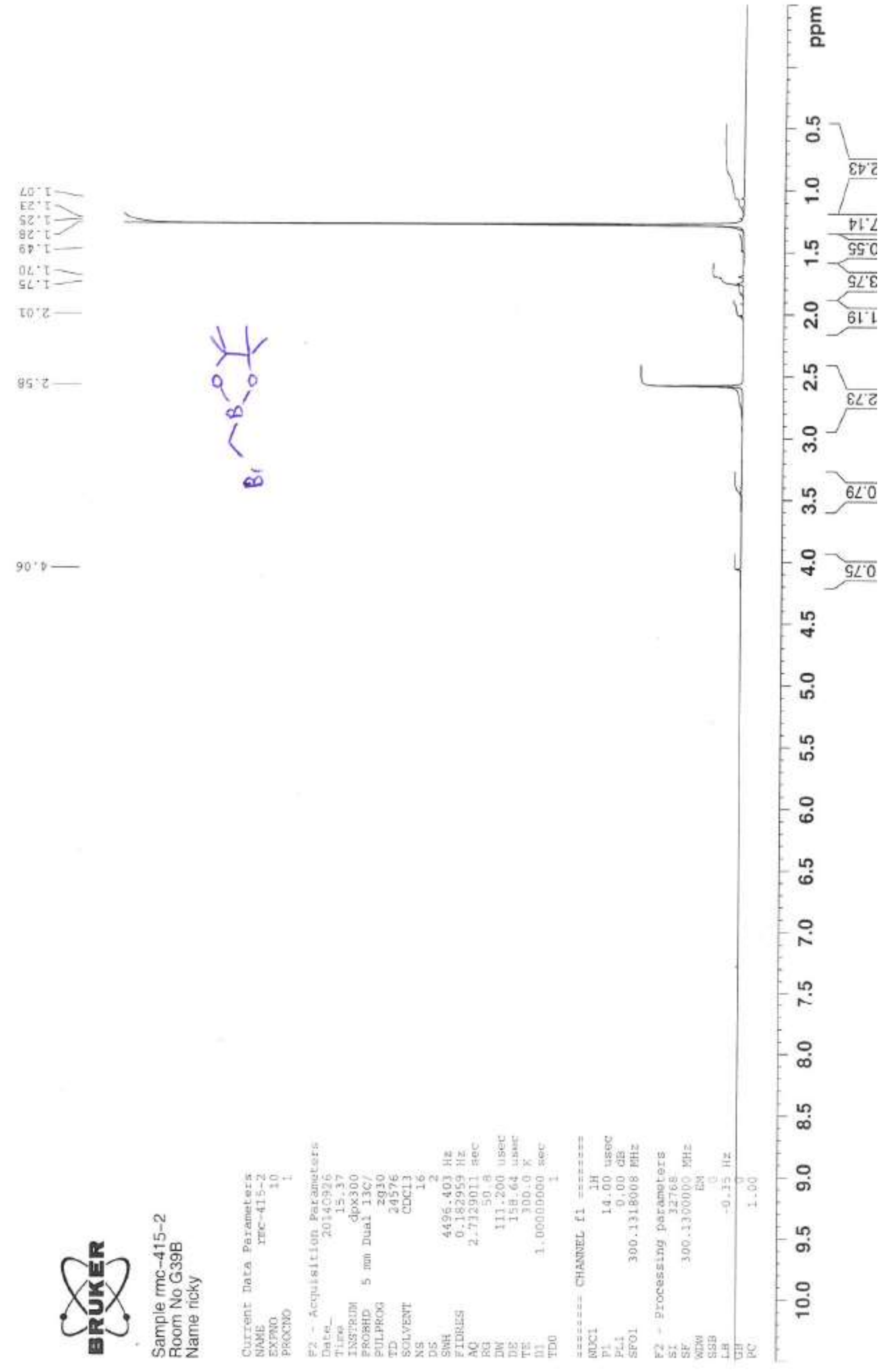

Supplementary Fig. 14. ^{1}H NMR spectrum of S2.

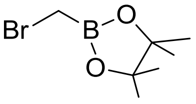

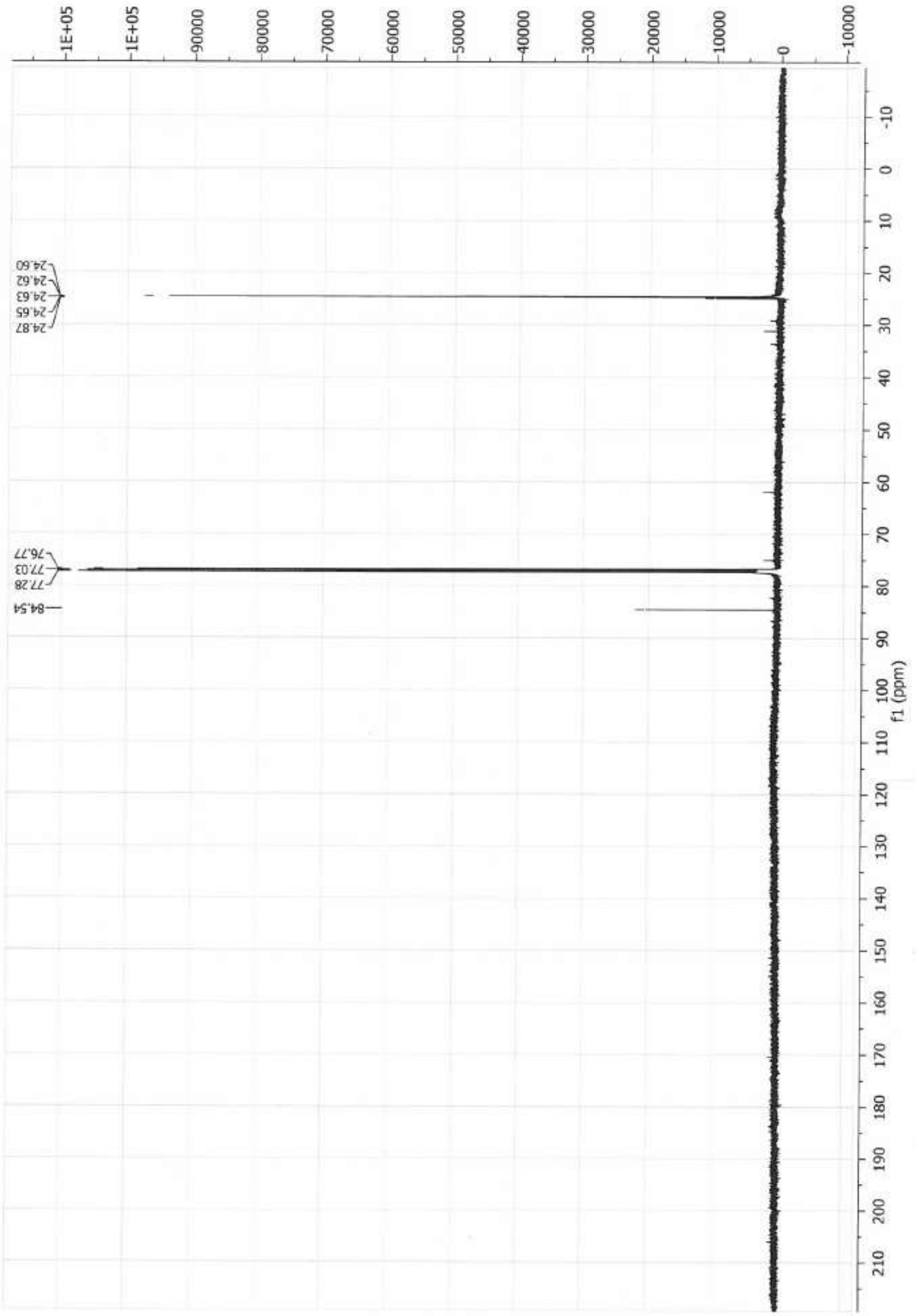

Supplementary Fig. 15. ^{13}C NMR spectrum of S2.

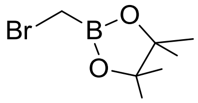

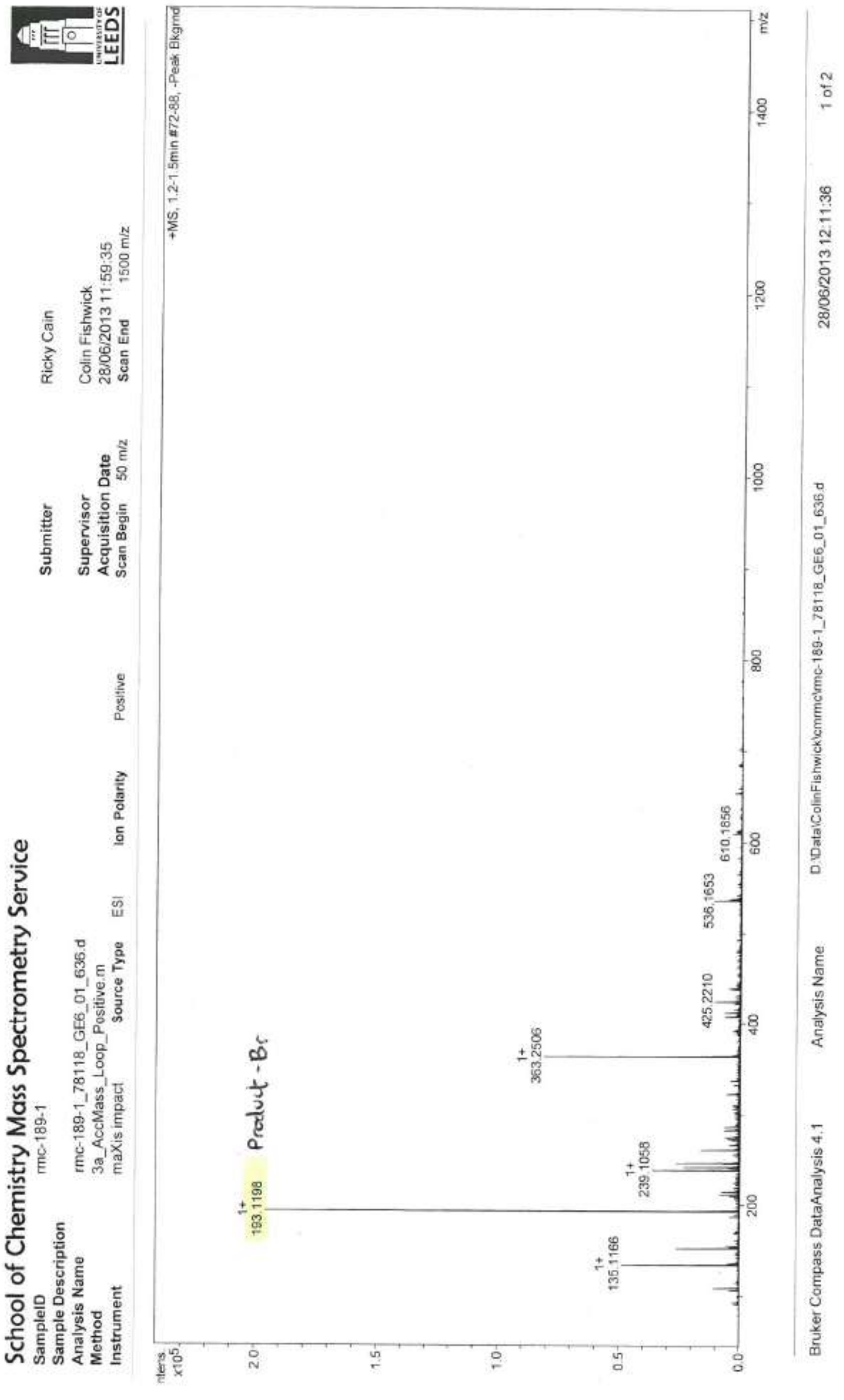

Supplementary Fig. 16. MS spectrum of S3.

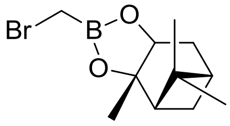

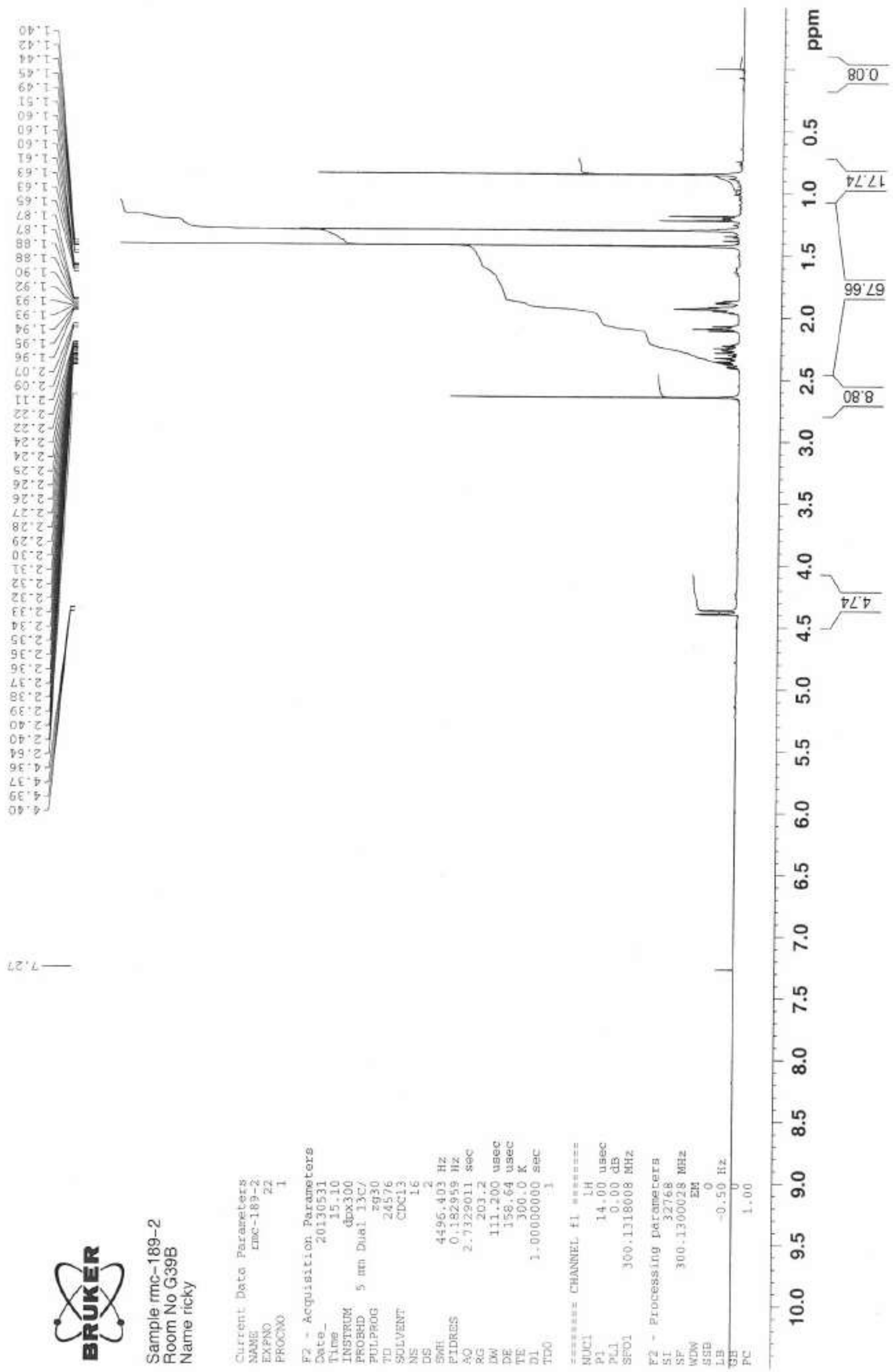

Supplementary Fig. 17. ^{1}H NMR spectrum of S3.

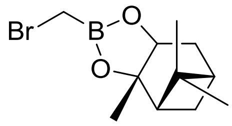

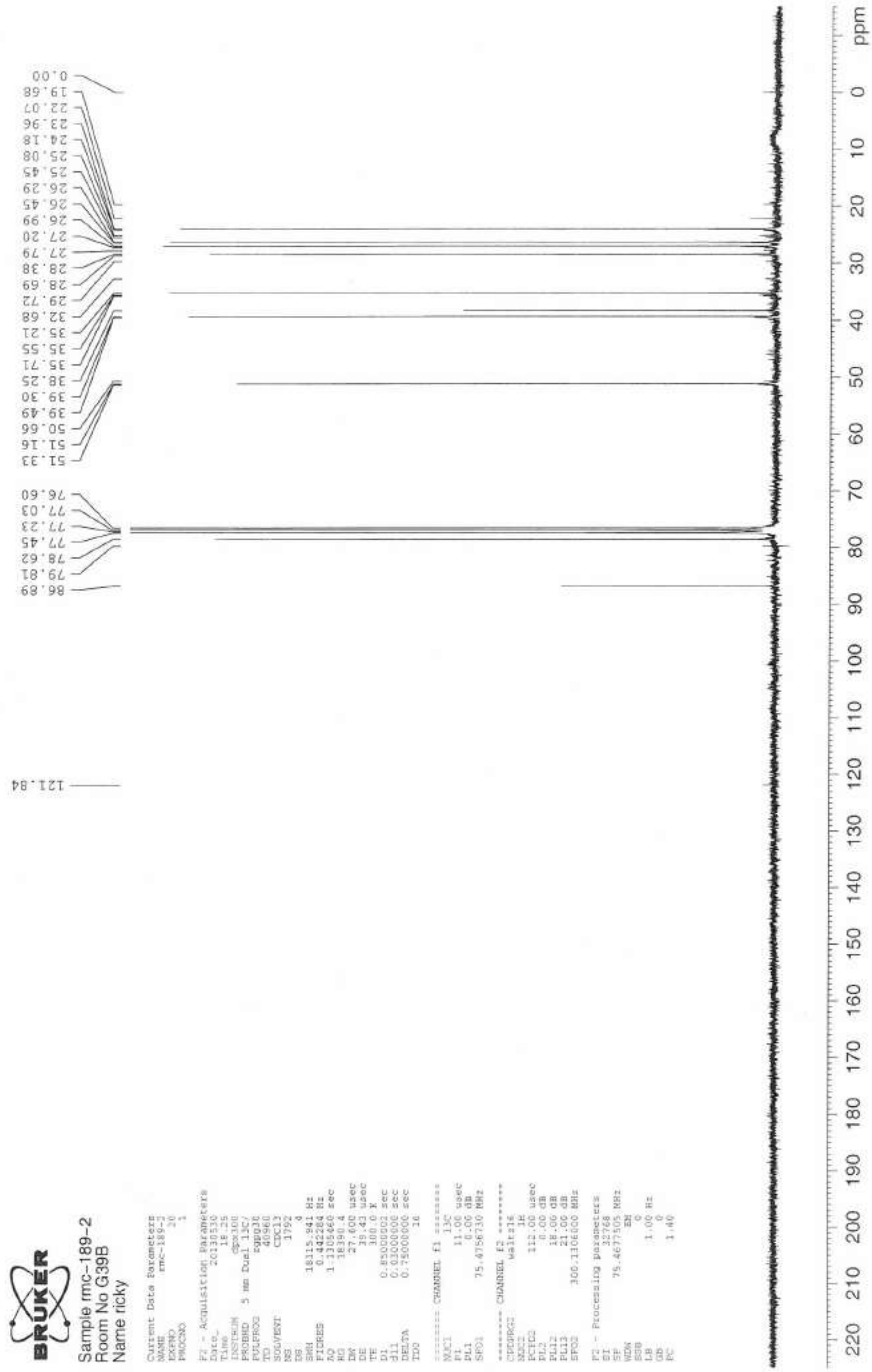

Supplementary Fig. 18. ^{13}C NMR spectrum of S3.

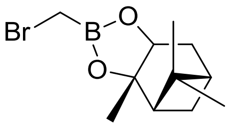

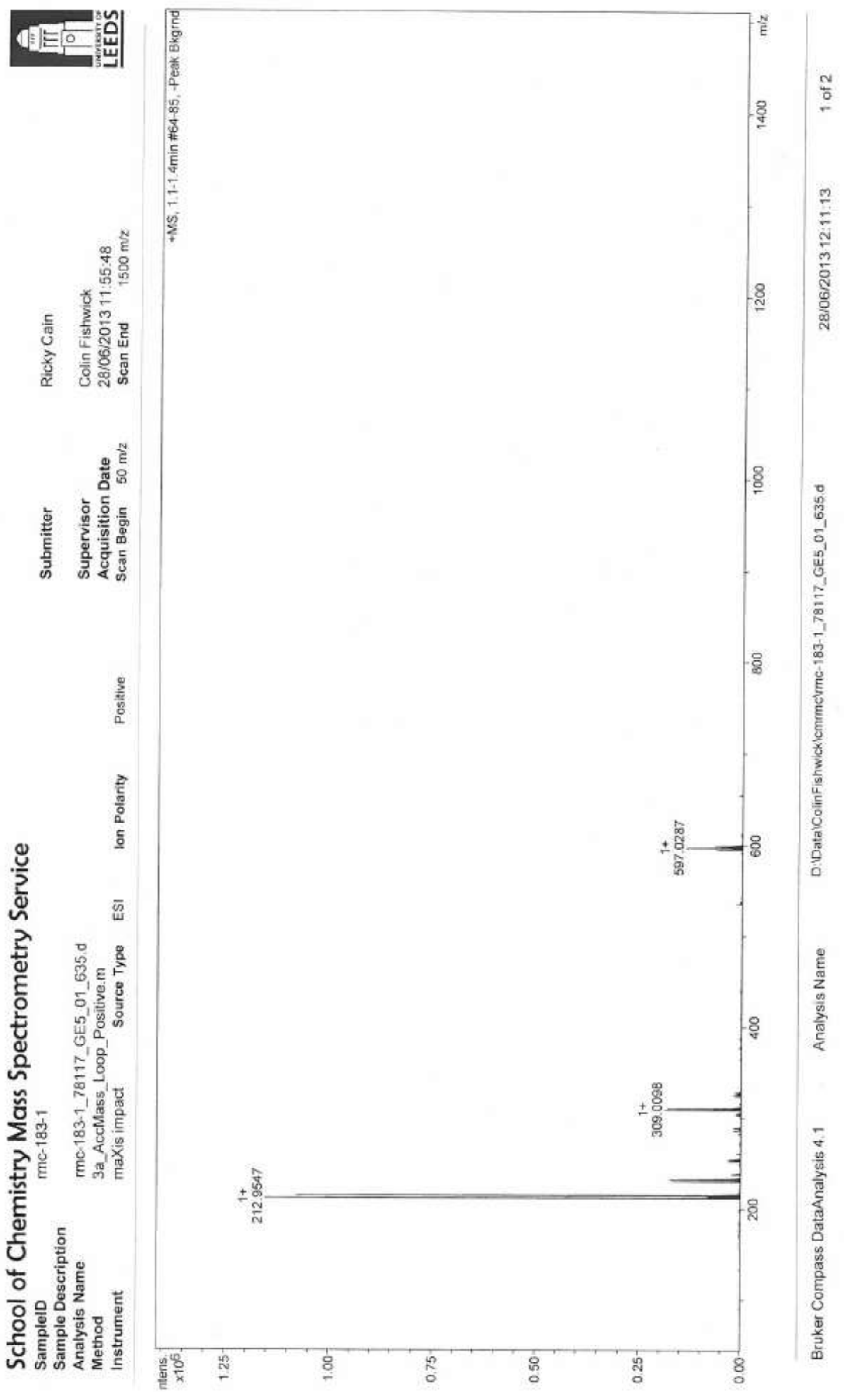

Supplementary Fig. 19. MS spectrum of S5.

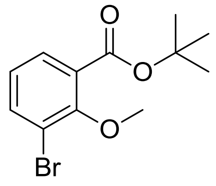

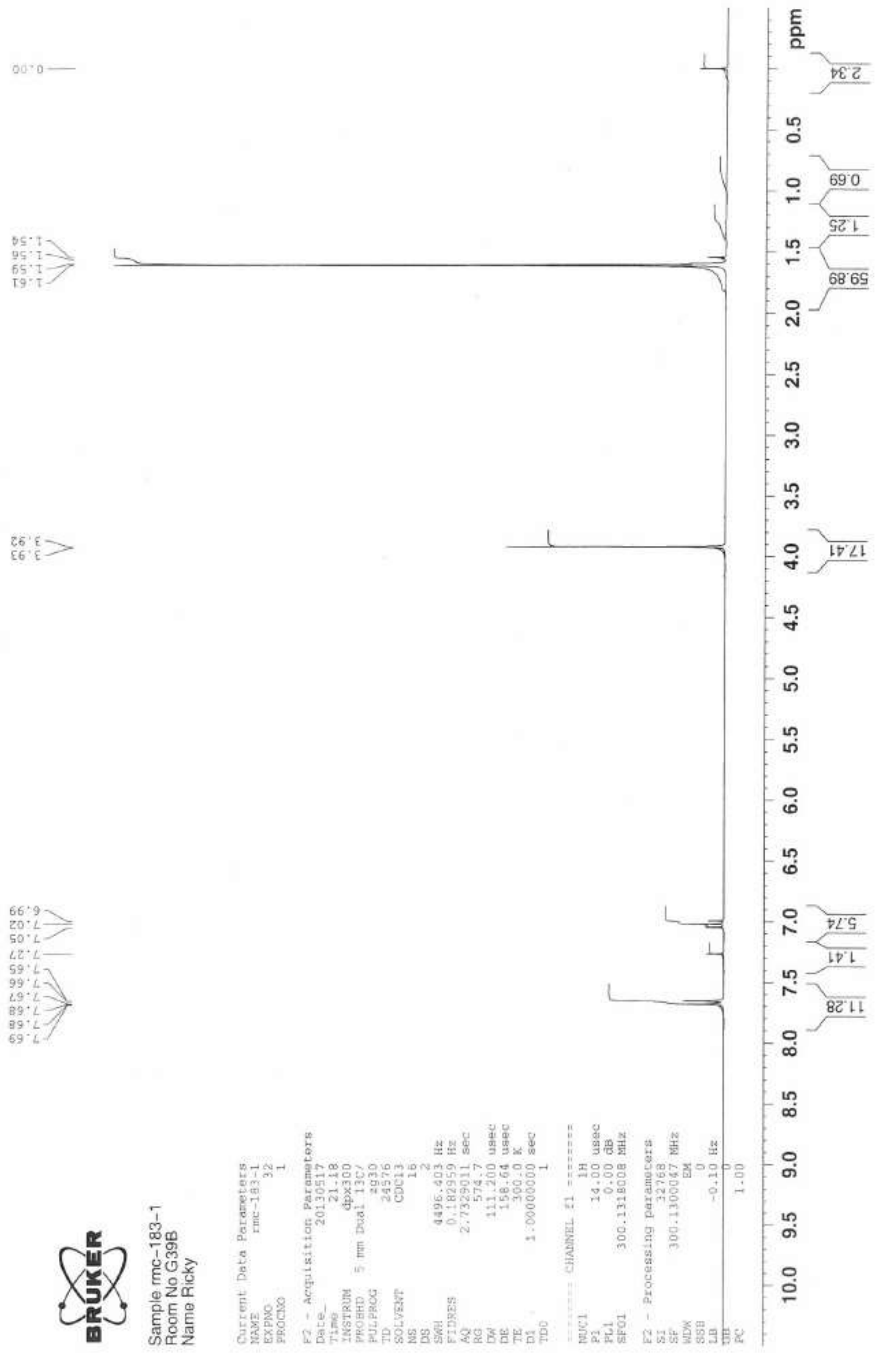

Supplementary Fig. 20. ^{1}H NMR spectrum of S5.

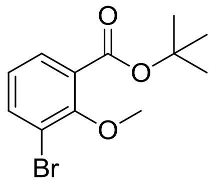

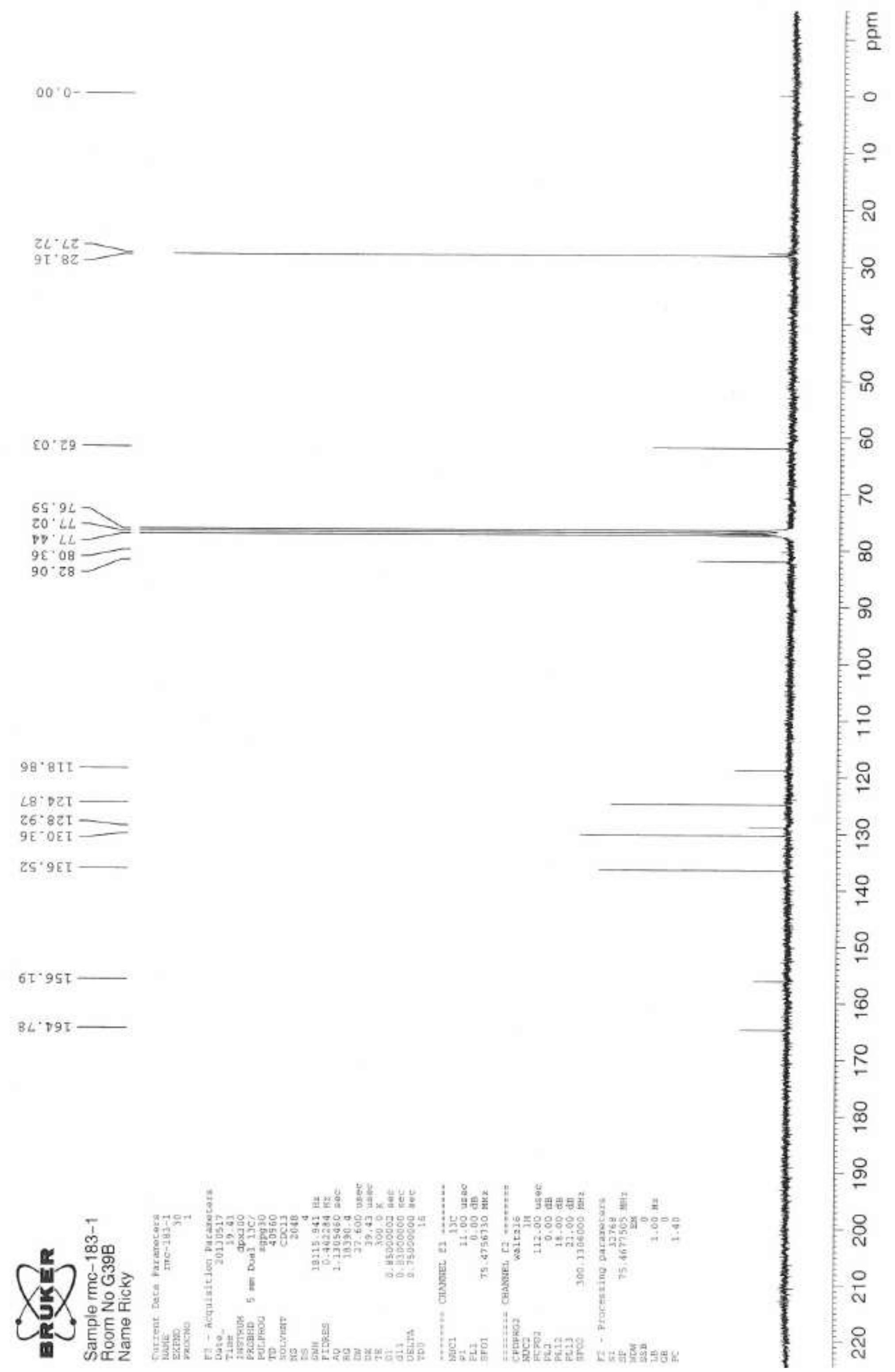

Supplementary Fig. 21. ^{13}C NMR spectrum of S5.

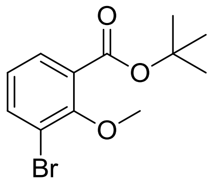

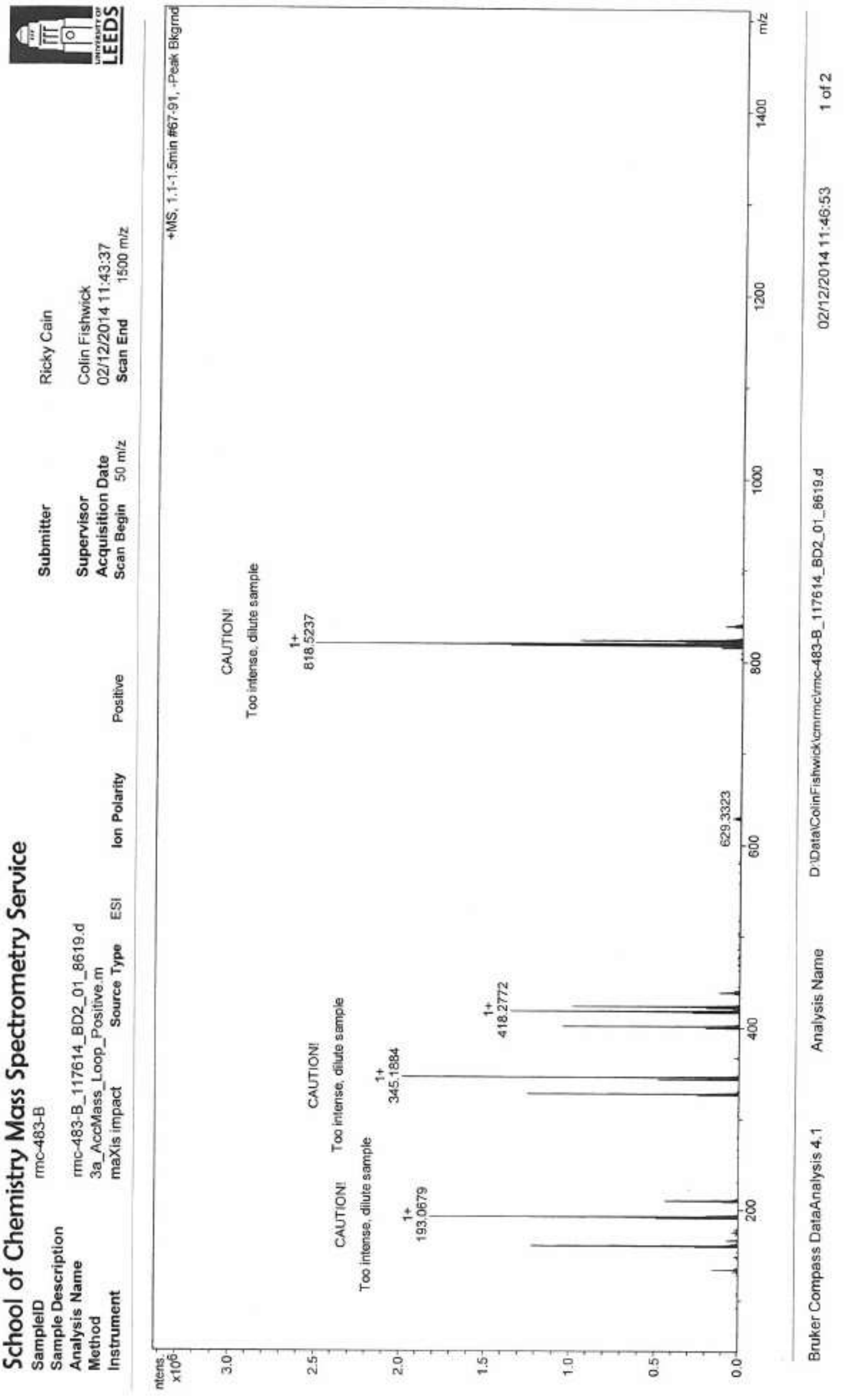

Supplementary Fig. 22. MS spectrum of S6.

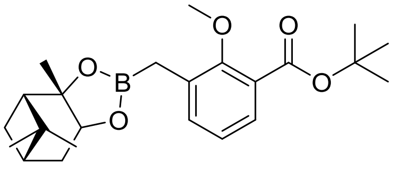

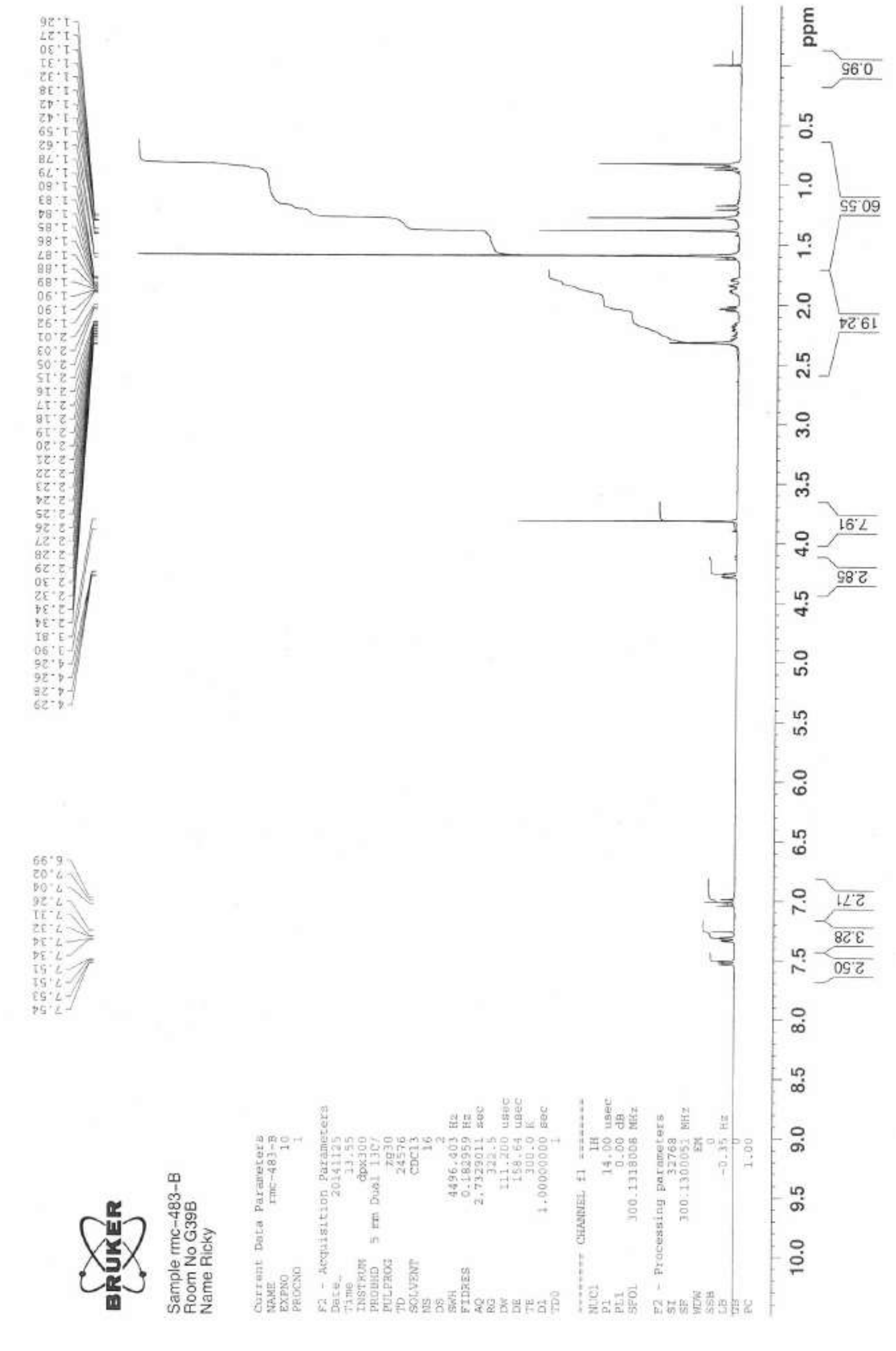

Supplementary Fig. 23. ^{1}H NMR spectrum of S6.

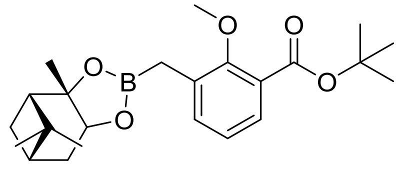

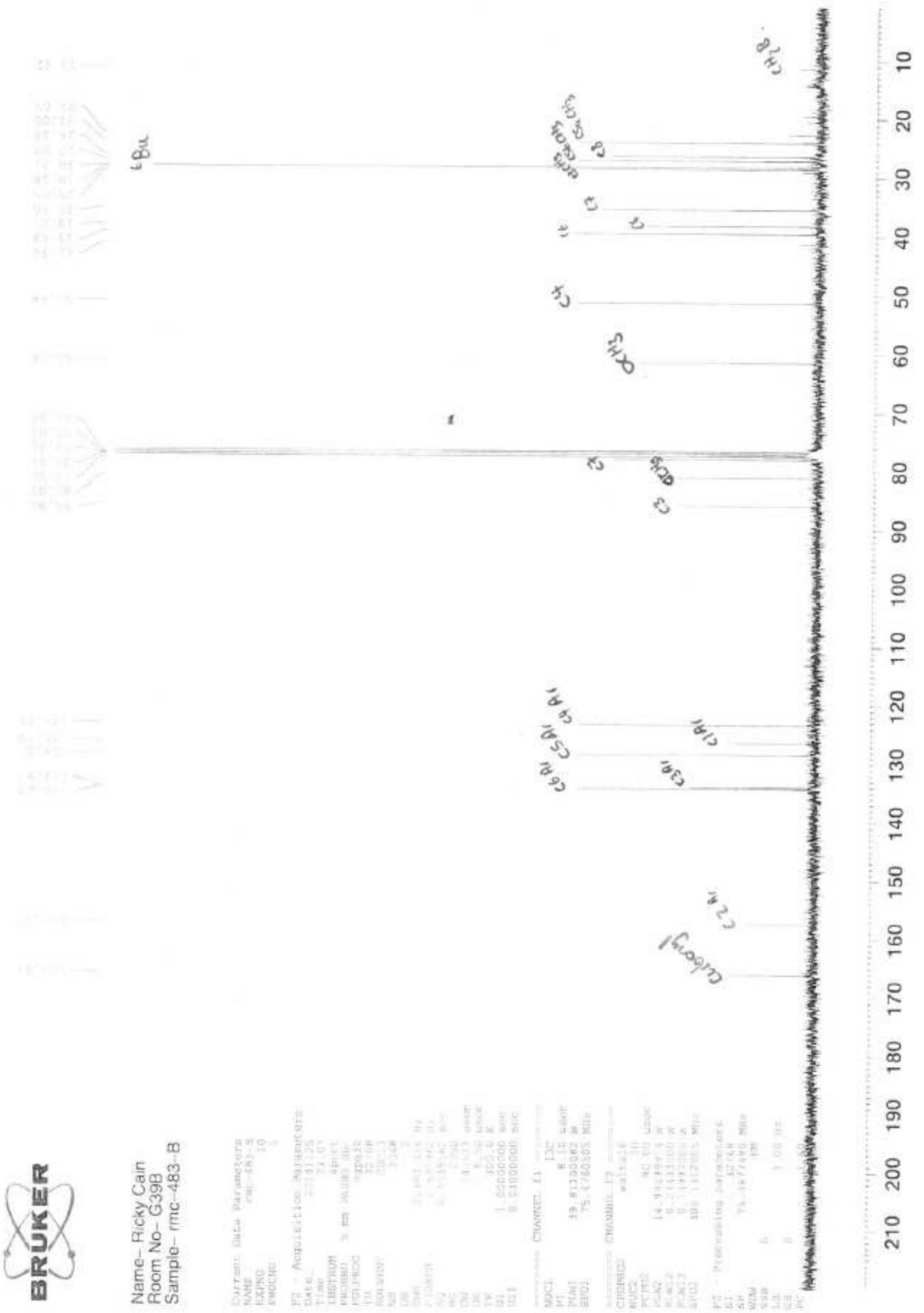

Supplementary Fig. 24. ^{13}C NMR spectrum of S6.

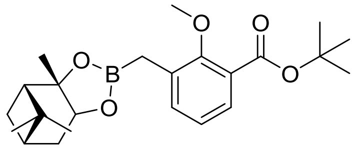

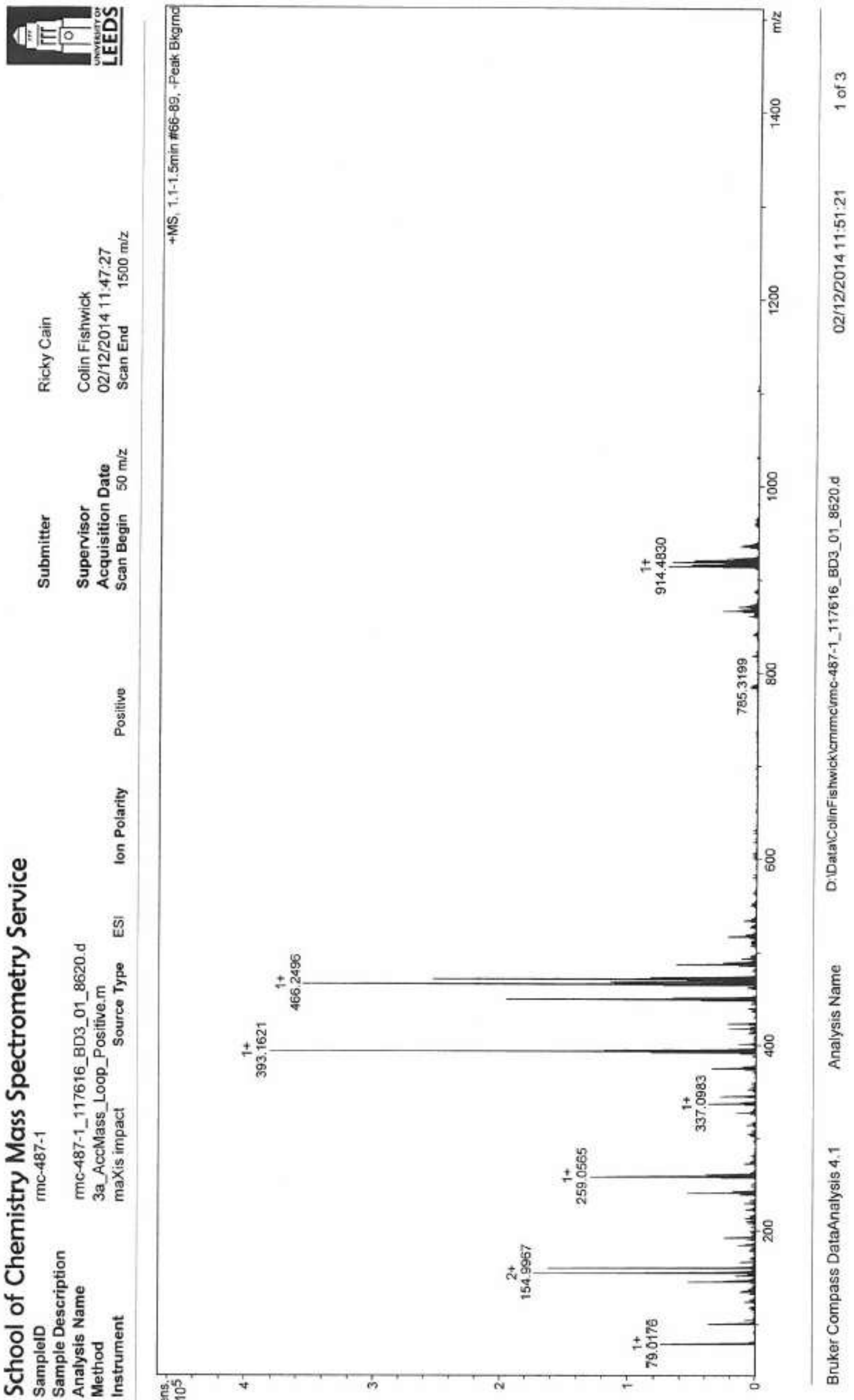

Supplementary Fig. 25. MS spectrum of S7.

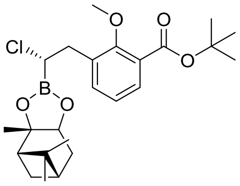

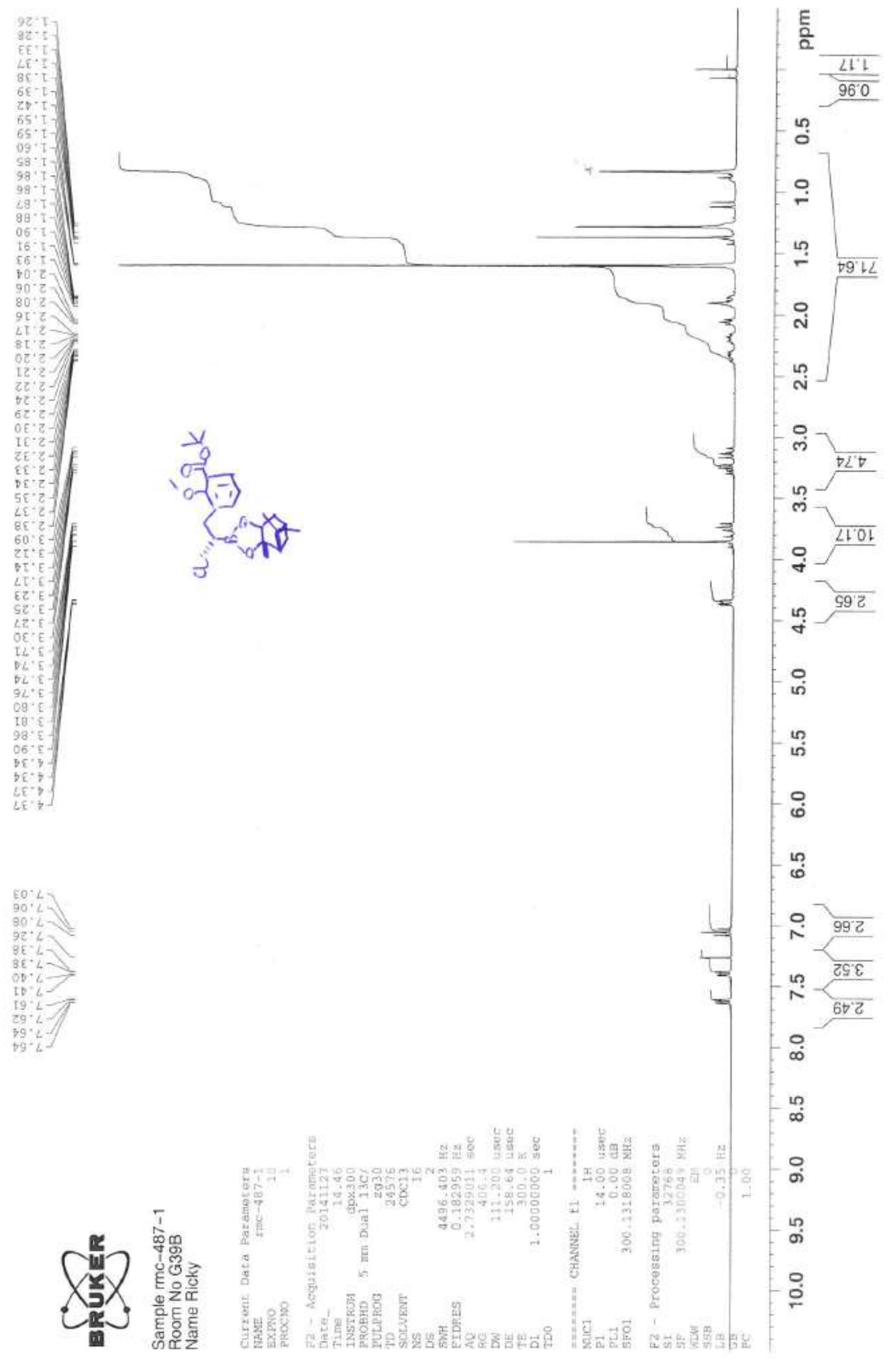

Supplementary Fig. 26. ^{1}H NMR spectrum of S7.

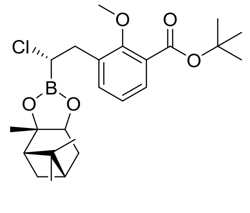

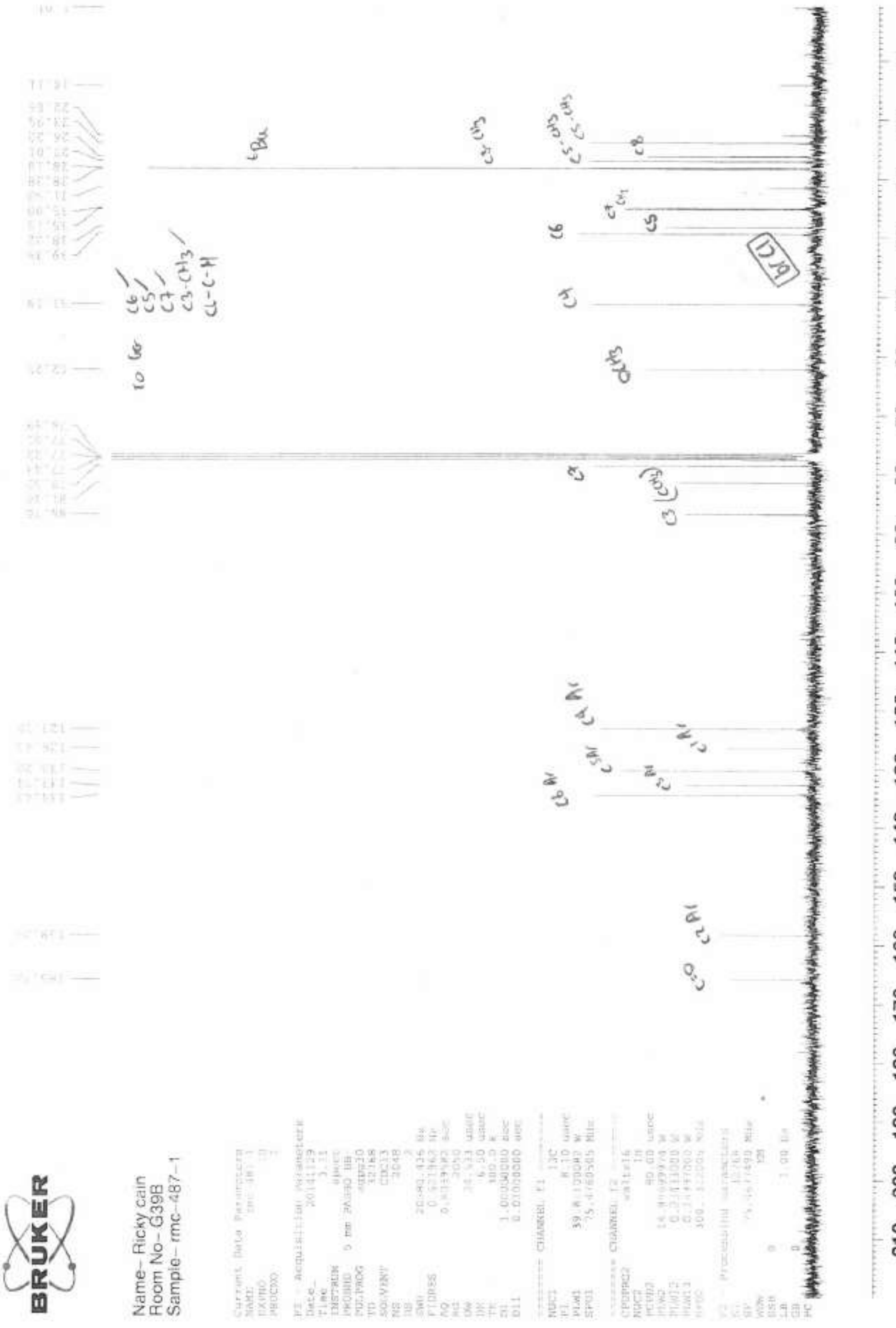

Supplementary Fig. 27. ^{13}C NMR spectrum of S7.

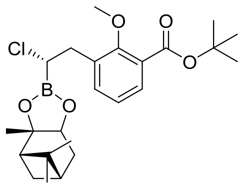

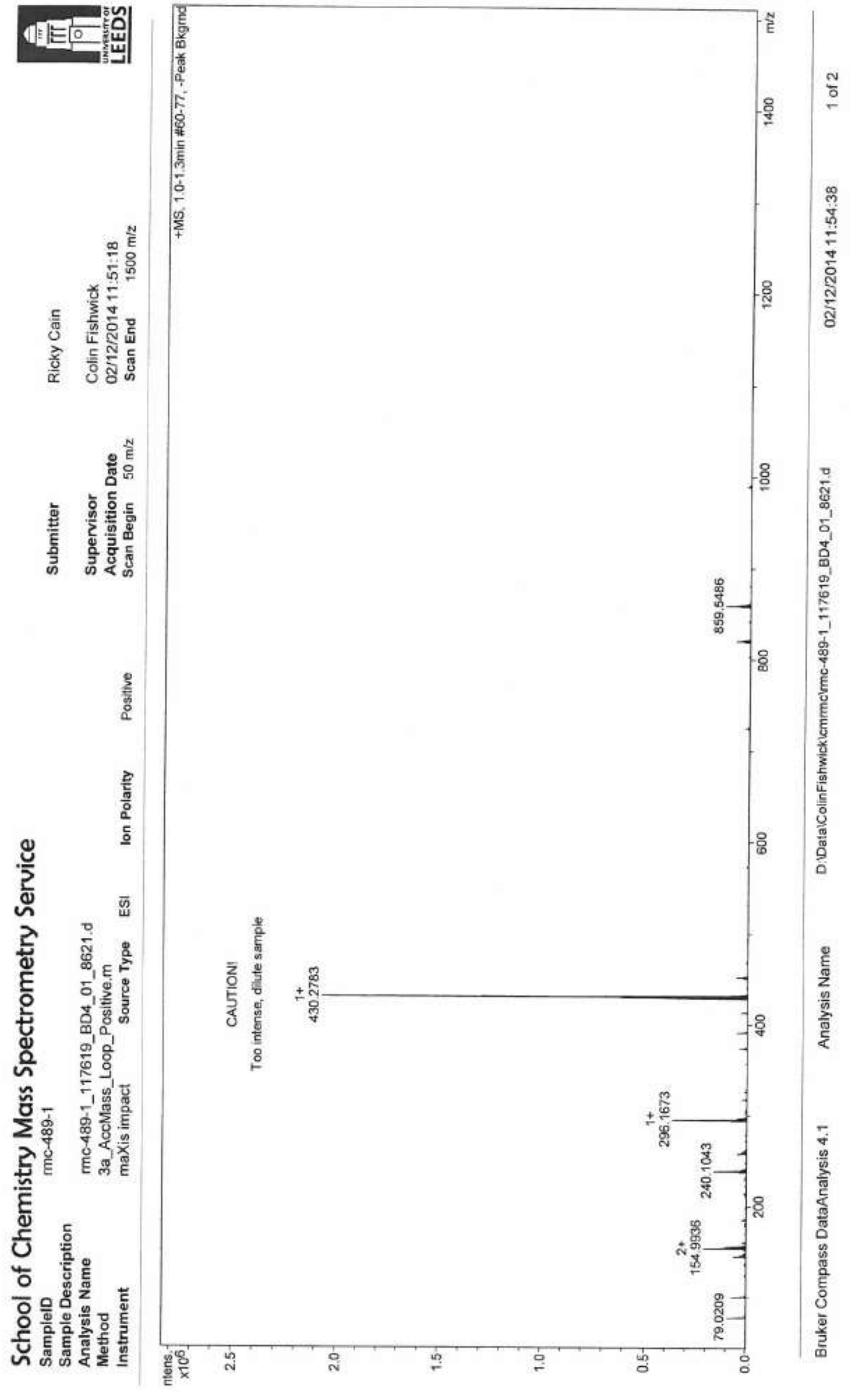

Supplementary Fig. 28. MS spectrum of S8.

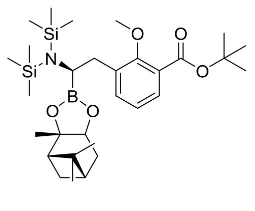

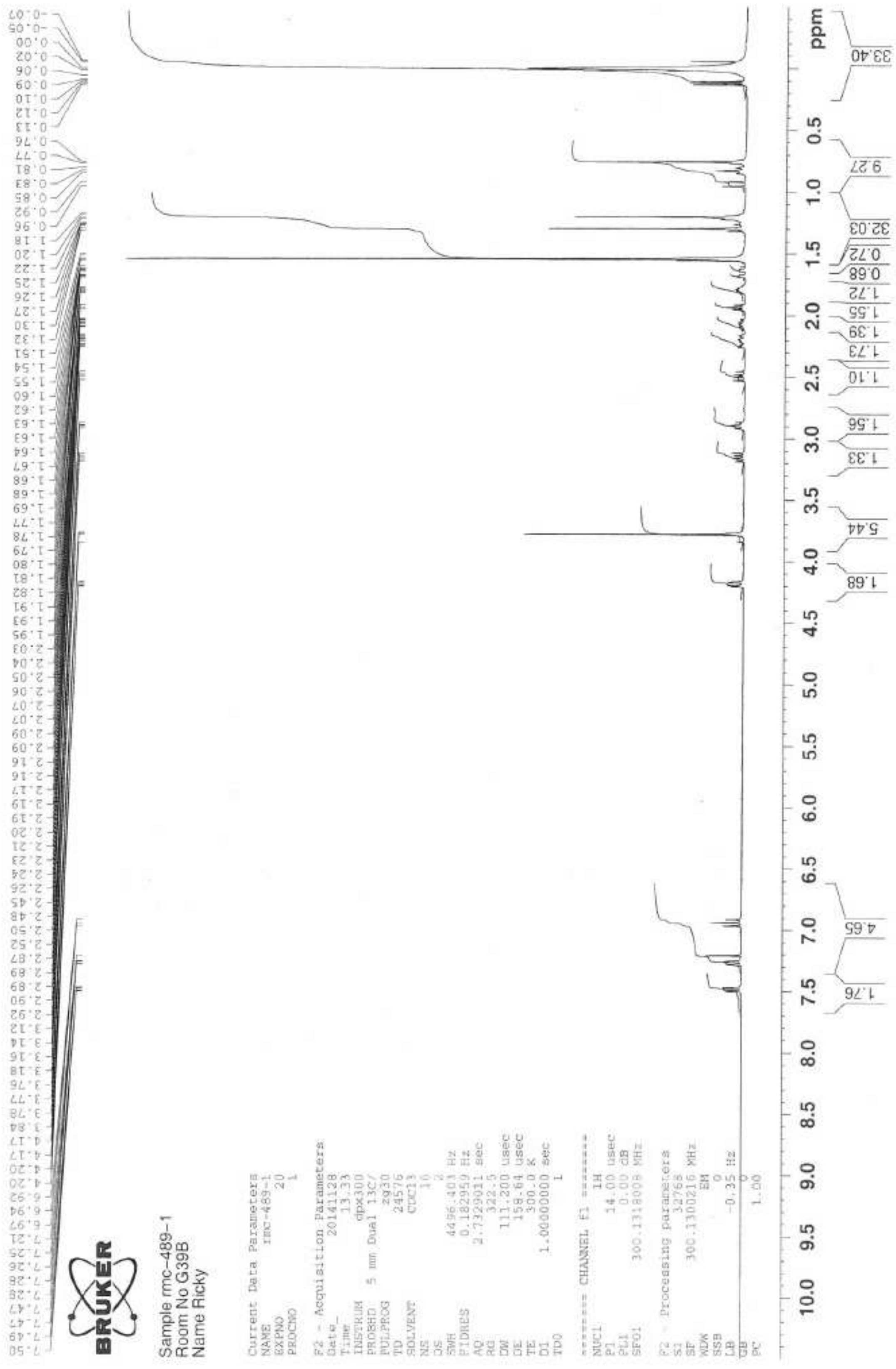

Supplementary Fig. 29. ^{1}H NMR spectrum of S8.

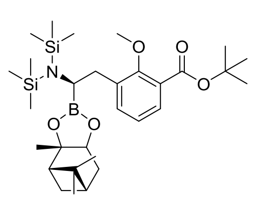

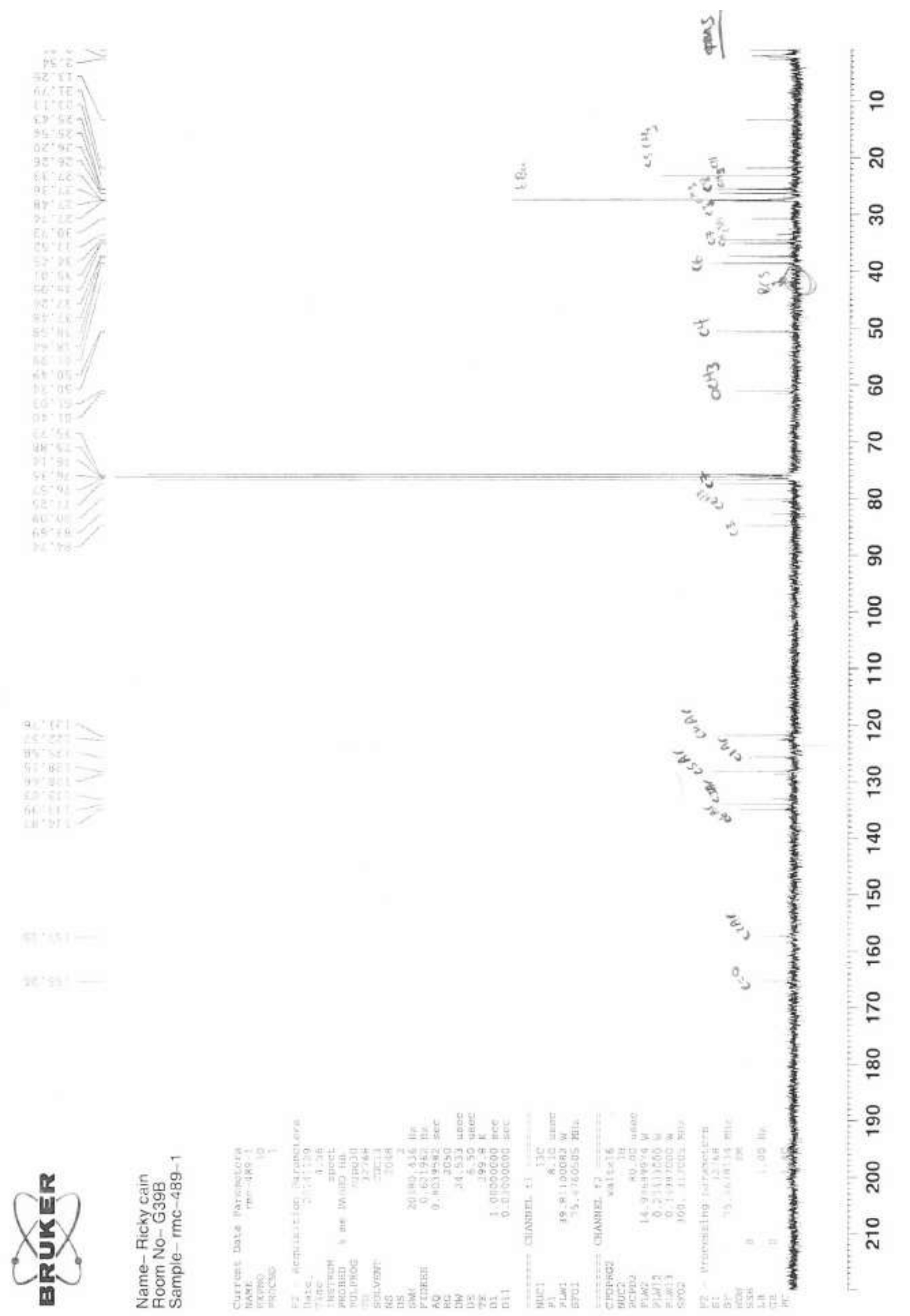

Supplementary Fig. 30. ^{13}C NMR spectrum of S8.

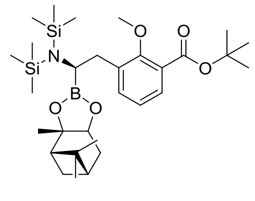

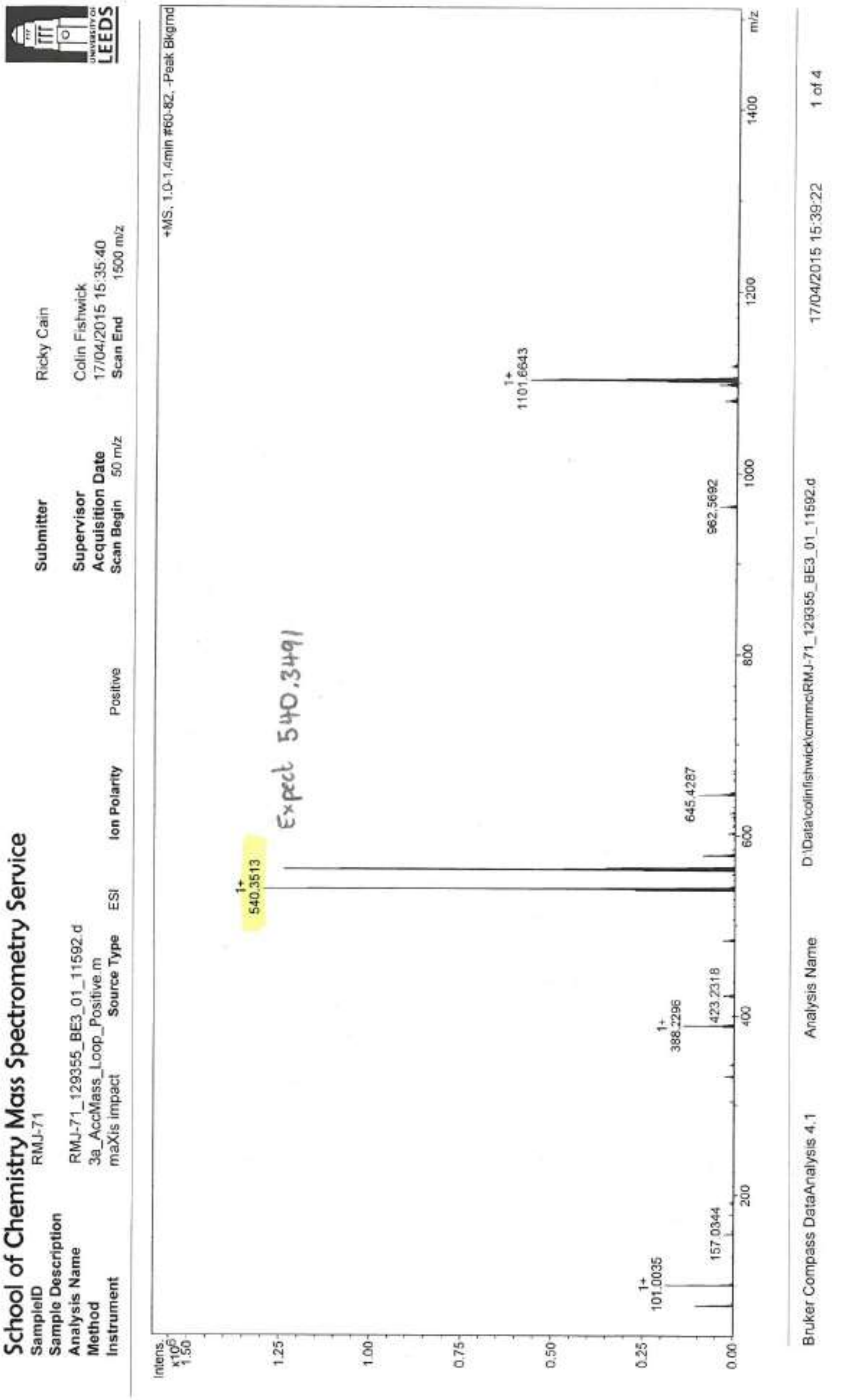

Supplementary Fig. 31. MS spectrum of S9a.

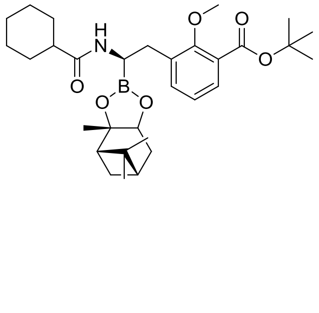

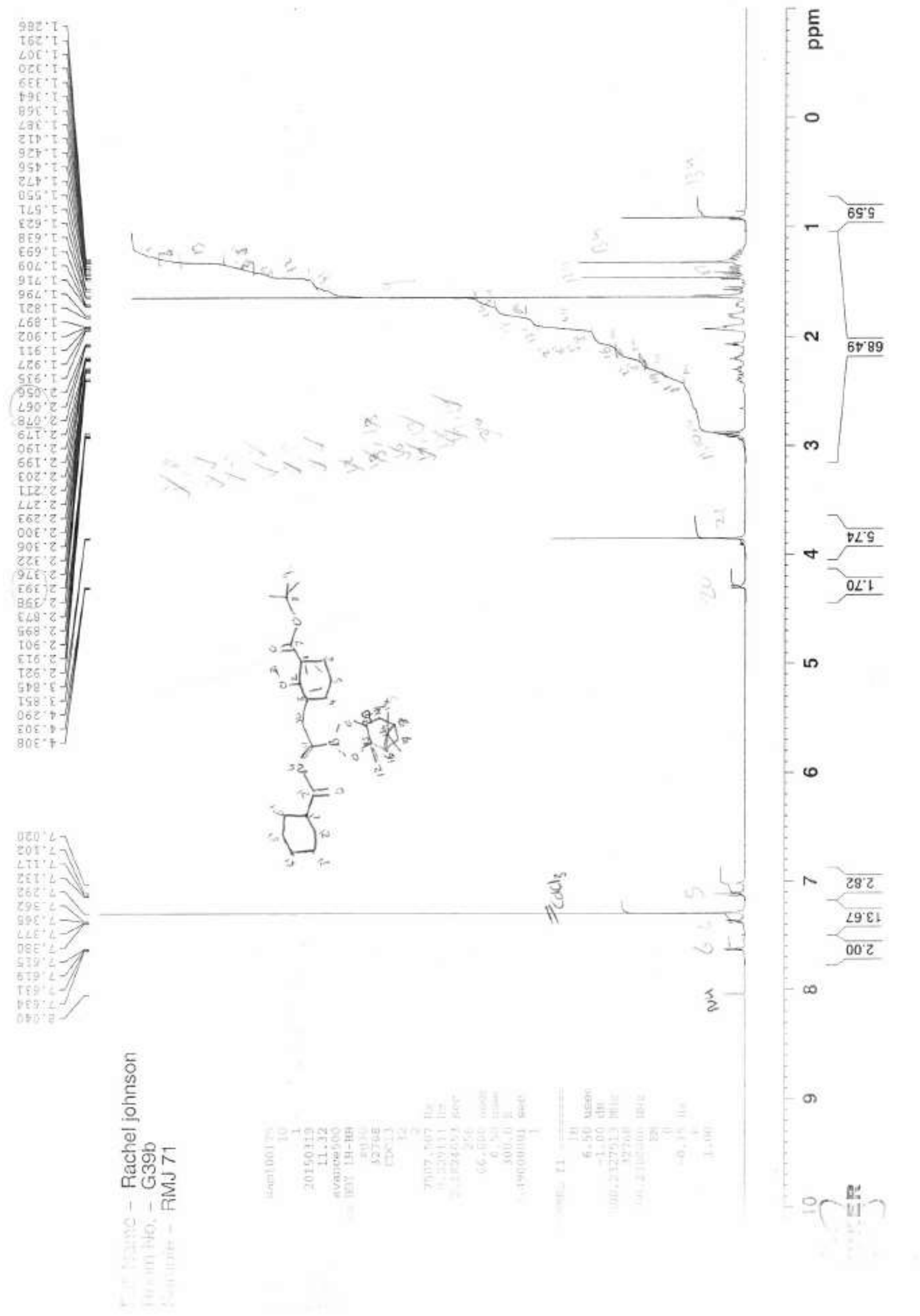

Supplementary Fig. 32. ^{1}H NMR spectrum of S9a.

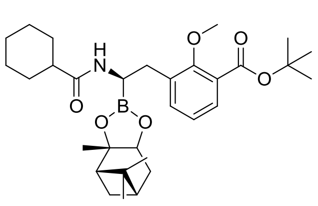

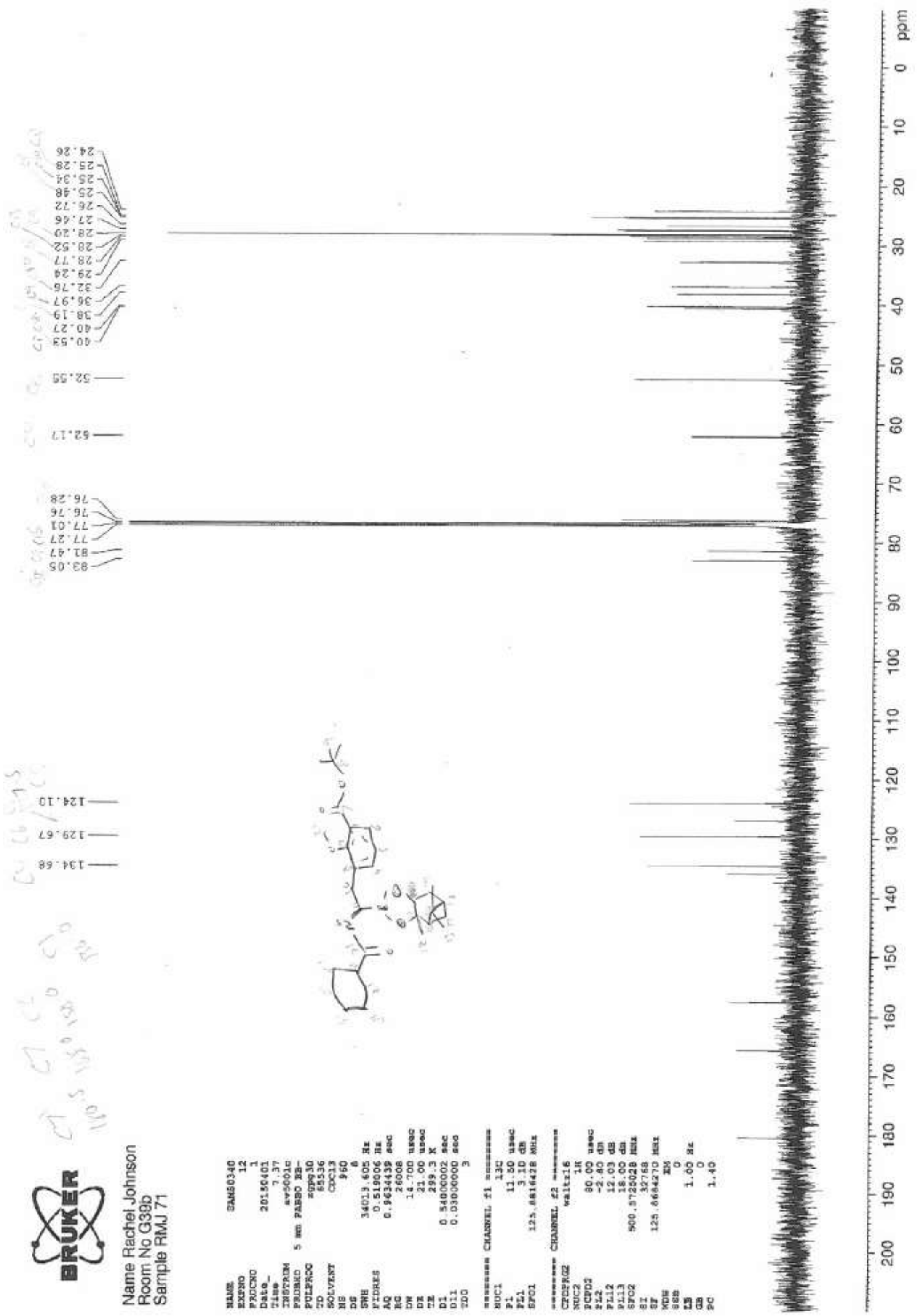

Supplementary Fig. 33. ^{13}C NMR spectrum of S9a.

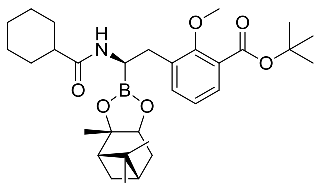

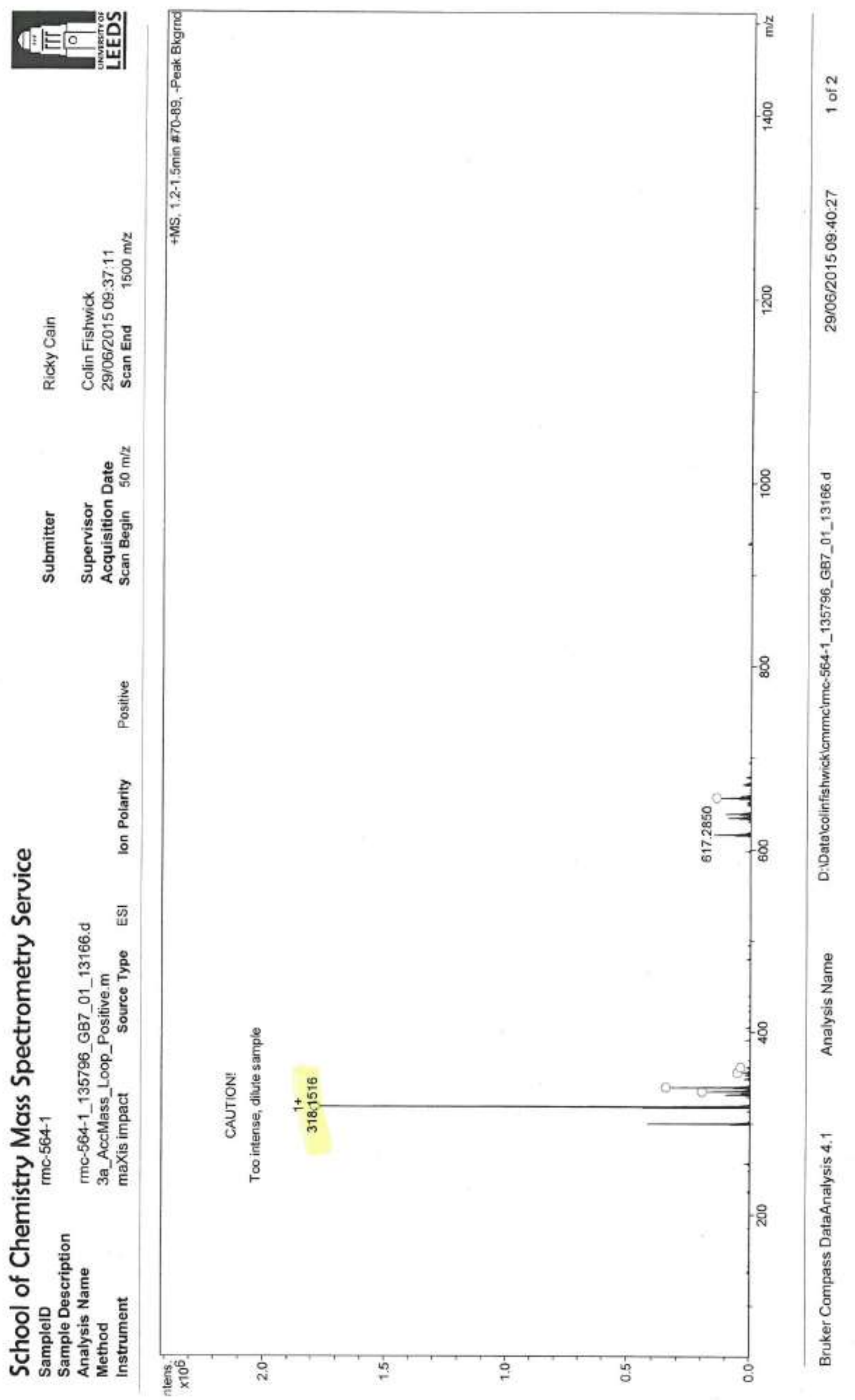

Supplementary Fig. 34. MS spectrum of 1.

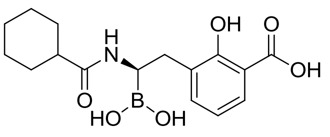

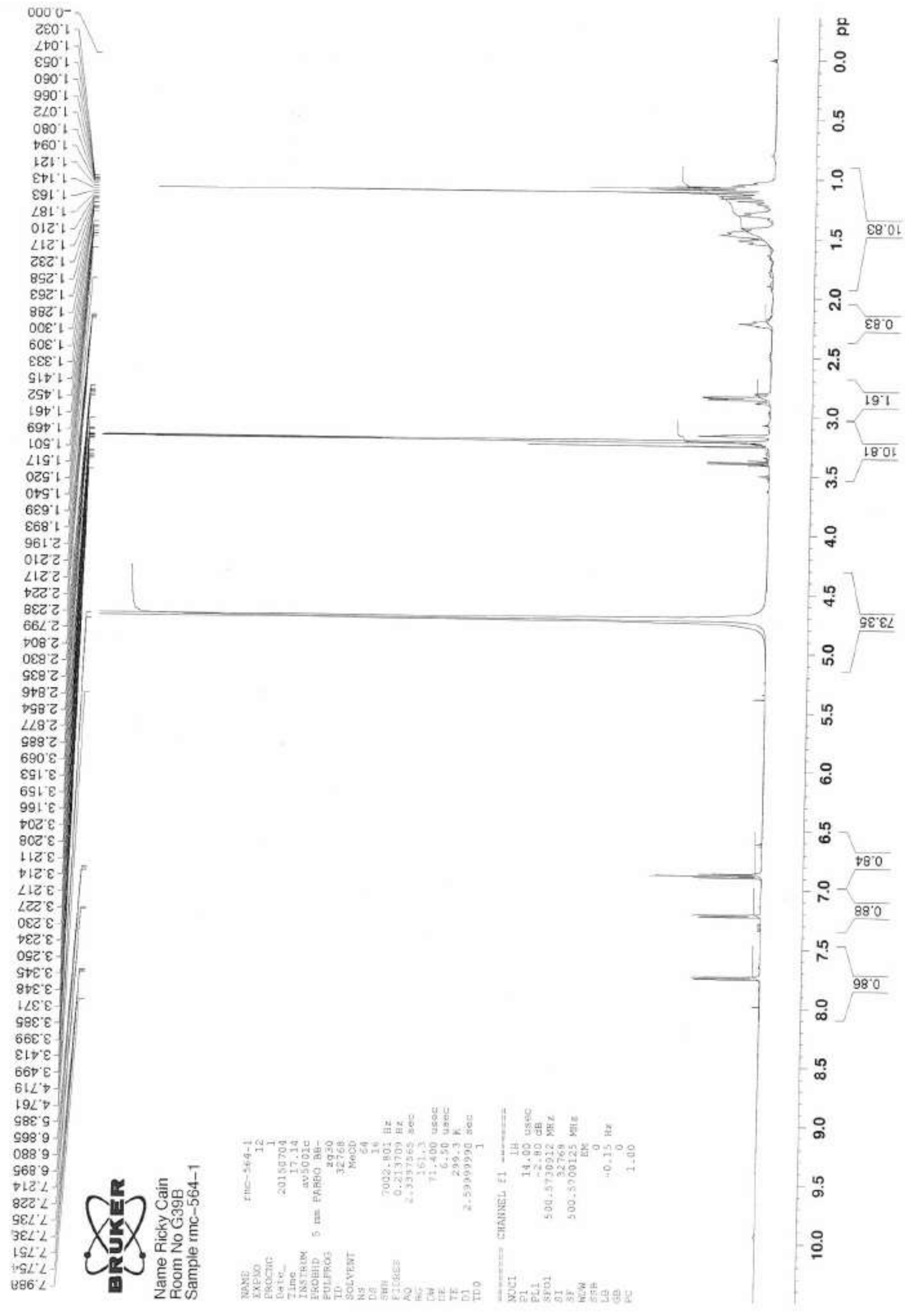

Supplementary Fig. 35. ^{1}H NMR spectrum of 1.

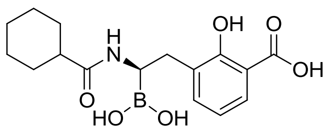

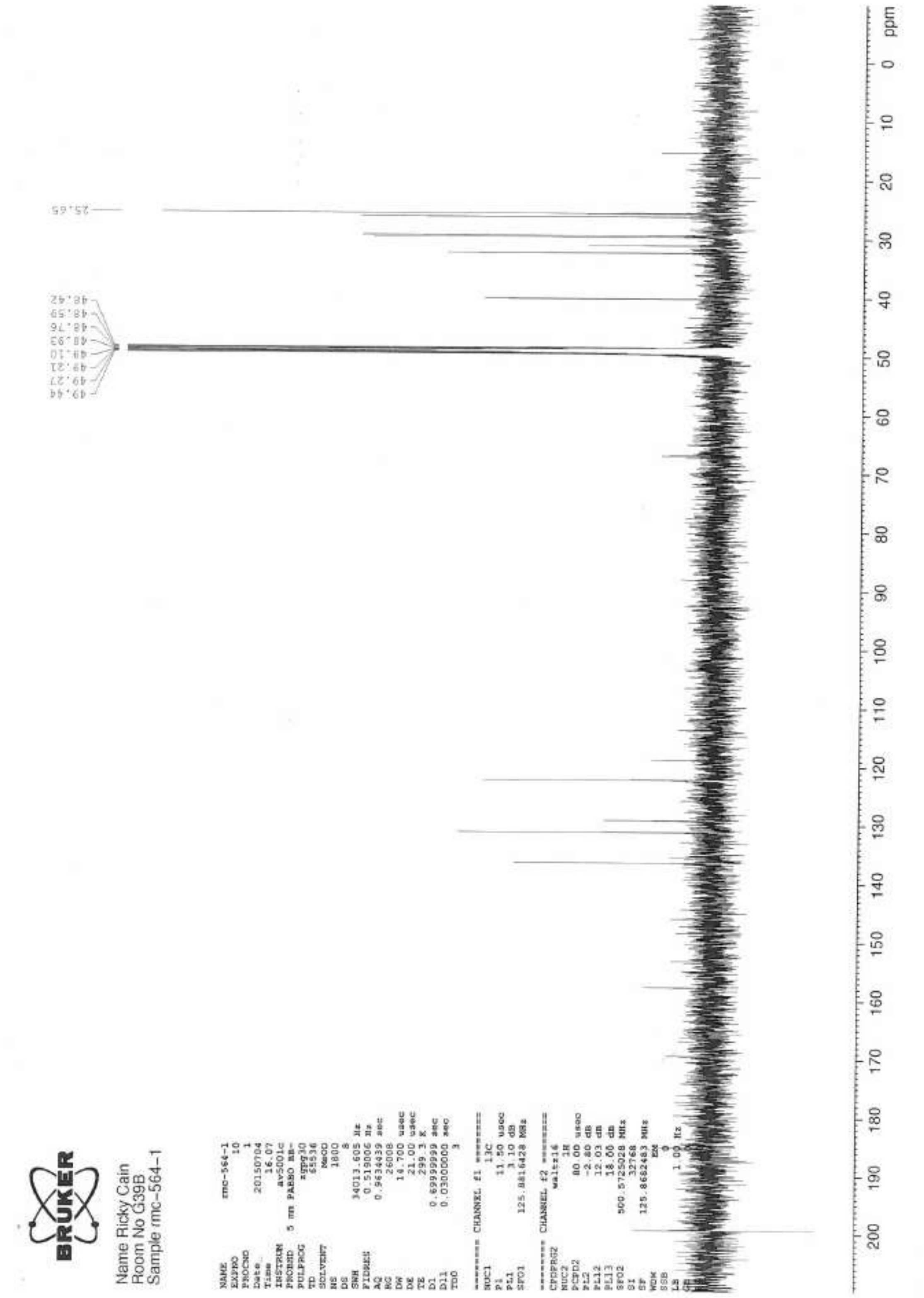

Supplementary Fig. 36. ^{13}C NMR spectrum of 1.

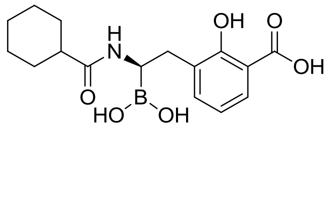

School of Chemistry Mass Spectrometry Service

**SampleID**	rmc-282-3	**Submitter**	Ricky Cain
**Sample Description**			
**Analysis Name**	rmc-282-3_126884_RD1_01_11067.d	**Supervisor**	Colin Fishwick
**Method**	3a_AccMass_Loop_Positive.m	**Acquisition Date**	17/03/2015 13:22:38
**Instrument**	maXis impact **Source Type** ESI **Ion Polarity** Positive	**Scan Begin** 50 m/z	**Scan End** 1500 m/z

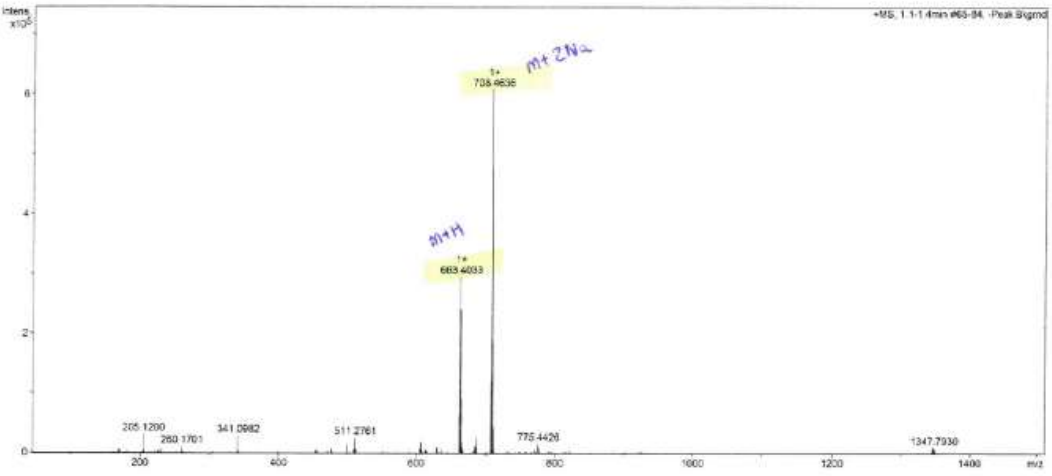

Bruker Compass DataAnalysis 4.1 | Analysis Name | D:\Data\ColinFishwick\rmc\rmc-282-3_126884_RD1_01_11067.d | 17/03/2015 13:26:04 | 1 of 4

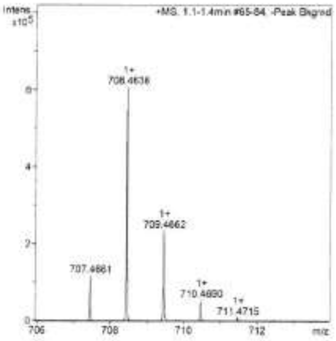

Smart Formula Parameter	Value
Expected Formula	C37H51BN2O8
Adducts Considered	M+H;M+NH4;M+Na;M+K;M+Na2-H;2M+H;2M+Na
Smart Formula Search Parameters	
CHNO and adducts considered implicitly	
Formula Search Minimum	
Formula Search Maximum	B 2
Algorithm Parameters	
Tolerance	4 ppm
Match to Isotope Pattern(mSigma)	40
Electron Configuration	even
Estimate No of Carbons	yes
Filter by H/C Ratio	0<= H/C < 3
Number of Double Bonds & Rings	0<= rings&DB < 80

Confirm/Find Formula Results

The section below shows the results of formula calculation. If an expected formula was provided and found these are the results that are listed. If no formula was provided or no matches were found the system has attempted to determine the formula constrained by the parameters listed to the left

Meas. m/z	Ion Formula	z	m/z	err [mDa]	err [ppm]	mSigma	Score	Sum Formula	Adduct
663.403320	C34H56BN2O10	1+	663.402858	0.5	0.7	7.2	100.00	C34 H55 B N2 O10	M+H
	C29H51N12O6	1+	663.404904	1.6	2.4	8.0	41.33	C29 H50 N12 O6	M+H
	C35H52BN6O6	1+	663.404207	-0.9	-1.3	8.8	78.16	C35 H51 B N6 O6	M+H
	C32H44BN16	1+	663.402808	-0.5	-0.8	9.5	93.63	C32 H43 B N16	M+H
	C36H48BN10O2	1+	663.405557	2.2	3.4	16.0	27.26	C36 H47 B N10 O2	M+H
	C28H55N8O10	1+	663.403566	0.2	0.4	18.7	70.65	C28 H54 N8 O10	M+H
	C27H59N4O14	1+	663.402229	1.1	1.6	30.1	35.40	C27 H58 N4 O14	M+H
	C34H56BN2O10	1+	663.402858	0.5	0.7	7.2	100.00	C34 H52 B N O10	M+NH4
	C29H51N12O6	1+	663.404904	1.6	2.4	8.0	41.33	C29 H47 N11 O6	M+NH4
	C35H52BN6O6	1+	663.404207	-0.9	-1.3	8.8	78.16	C35 H48 B N5 O6	M+NH4
	C32H44BN16	1+	663.402808	-0.5	-0.8	9.5	93.63	C32 H40 B N15	M+NH4
	C36H48BN10O2	1+	663.405557	2.2	3.4	16.0	27.26	C36 H44 B N9 O2	M+NH4
	C28H55N8O10	1+	663.403566	0.2	0.4	18.7	70.65	C28 H51 N7 O10	M+NH4
	C27H59N4O14	1+	663.402229	1.1	1.6	30.1	35.40	C27 H55 N3 O14	M+NH4
	C34H49BN10NaO2	1+	663.403121	0.2	0.3	9.2	100.00	C34 H49 B N10 O2	M+Na
	C37H57BNaO8	1+	663.404520	1.2	1.8	9.8	58.85	C37 H57 B O8	M+Na
	C28H48N16NaO2	1+	663.403838	0.5	0.8	10.4	67.64	C28 H48 N16 O2	M+Na
	C30H60N2NaO12	1+	663.403846	0.5	0.8	17.7	58.21	C30 H60 N2 O12	M+Na
	C27H52N12NaO6	1+	663.402498	0.8	1.2	19.2	48.55	C27 H52 N12 O6	M+Na
	C31H53BKN10O3	1+	663.403227	0.1	0.1	25.9	100.00	C31 H53 B N10 O3	M+K
	C30H57BKN6O7	1+	663.401877	-1.4	-2.2	27.1	47.48	C30 H57 B N6 O7	M+K
	C34H61BKO9	1+	663.404627	1.3	2.0	31.0	47.12	C34 H61 B O9	M+K
	C35H57BKN4O5	1+	663.405977	-2.7	-4.0	32.4	16.34	C35 H57 B N4 O5	M+K
	C28H60KN6O9	1+	663.405336	-2.0	-3.0	34.0	22.30	C28 H60 N6 O9	M+K
	C33H61Na2O10	1+	663.405463	2.1	3.2	5.4	25.53	C33 H62 O10	M+Na2-H
	C35H58BNa2O8	1+	663.402084	1.2	1.9	6.9	58.75	C35 H59 B O8	M+Na2-H
	C30H53N10Na2O4	1+	663.404115	-0.8	-1.2	8.2	59.15	C30 H54 N10 O4	M+Na2-H
	C36H54BN4Na2O4	1+	663.403433	0.1	0.2	9.3	100.00	C36 H55 B N4 O4	M+Na2-H
	C31H49N14Na2	1+	663.405455	2.1	3.2	9.5	23.92	C31 H50 N14	M+Na2-H
	C37H50BN8Na2	1+	663.404783	-1.5	-2.2	16.7	42.14	C37 H51 B N8	M+Na2-H
	C29H57N6Na2O8	1+	663.402778	-0.5	-0.8	17.8	55.86	C29 H58 N6 O8	M+Na2-H
	C28H55N8O10	1+	663.403560	0.2	0.4	18.7	70.65	C14 H27 N4 O5	2M+H
	C28H48N16NaO2	1+	663.403836	0.5	0.8	10.4	67.64	C14 H24 N8 O	2M+Na
	C30H60N2NaO12	1+	663.403846	0.5	0.8	17.7	58.21	C15 H30 N O6	2M+Na

Bruker Compass DataAnalysis 4.1 | Analysis Name | D:\Data\ColinFishwick\rmc\rmc-282-3_126884_RD1_01_11067.d | 17/03/2015 13:26:04 | 2 of 4

Supplementary Fig. 37. MS spectrum of S9b.

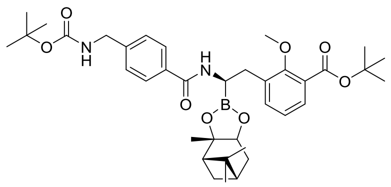

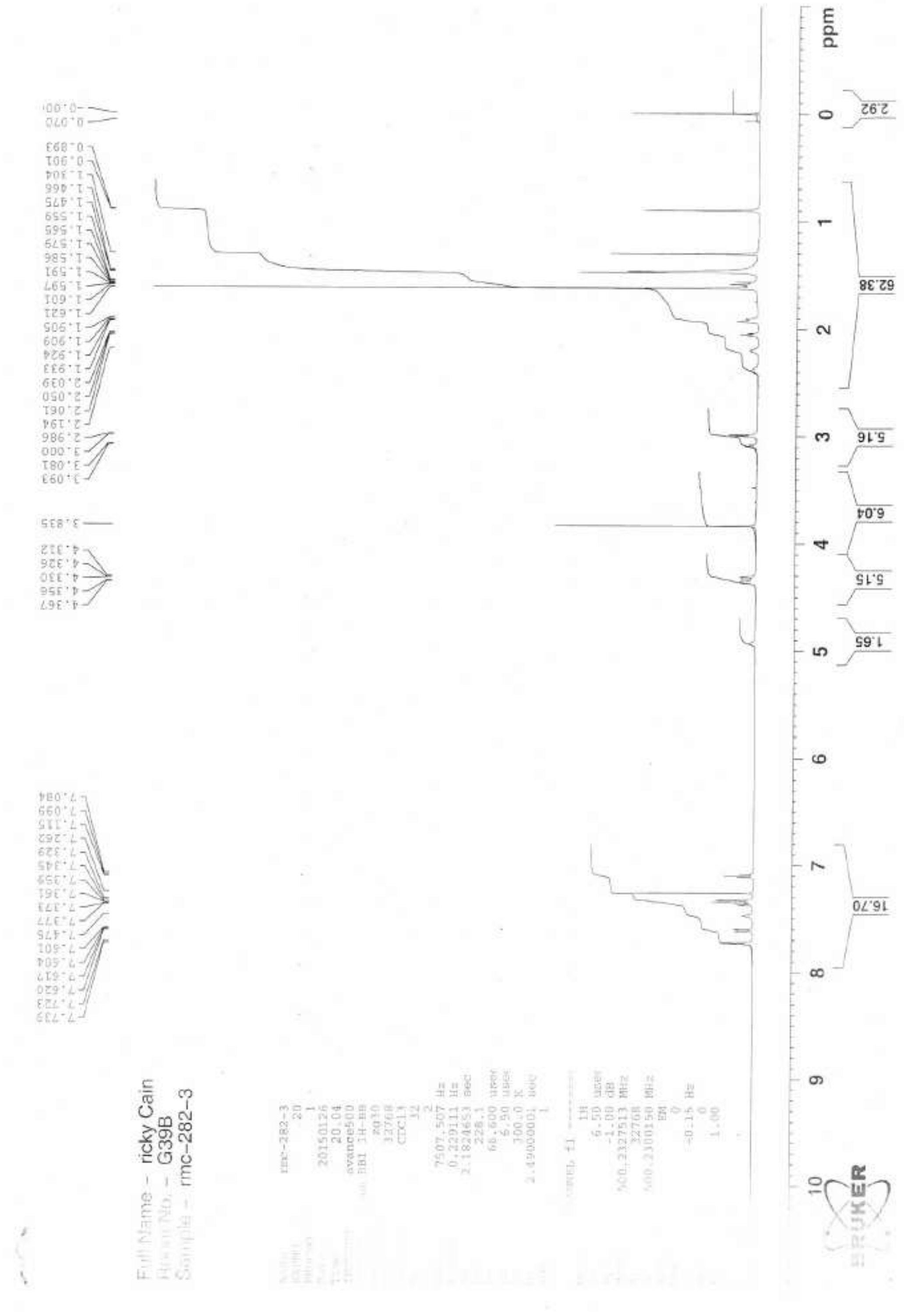

Supplementary Fig. 38. ^{1}H NMR spectrum of S9b.

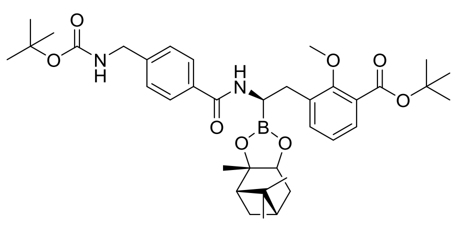

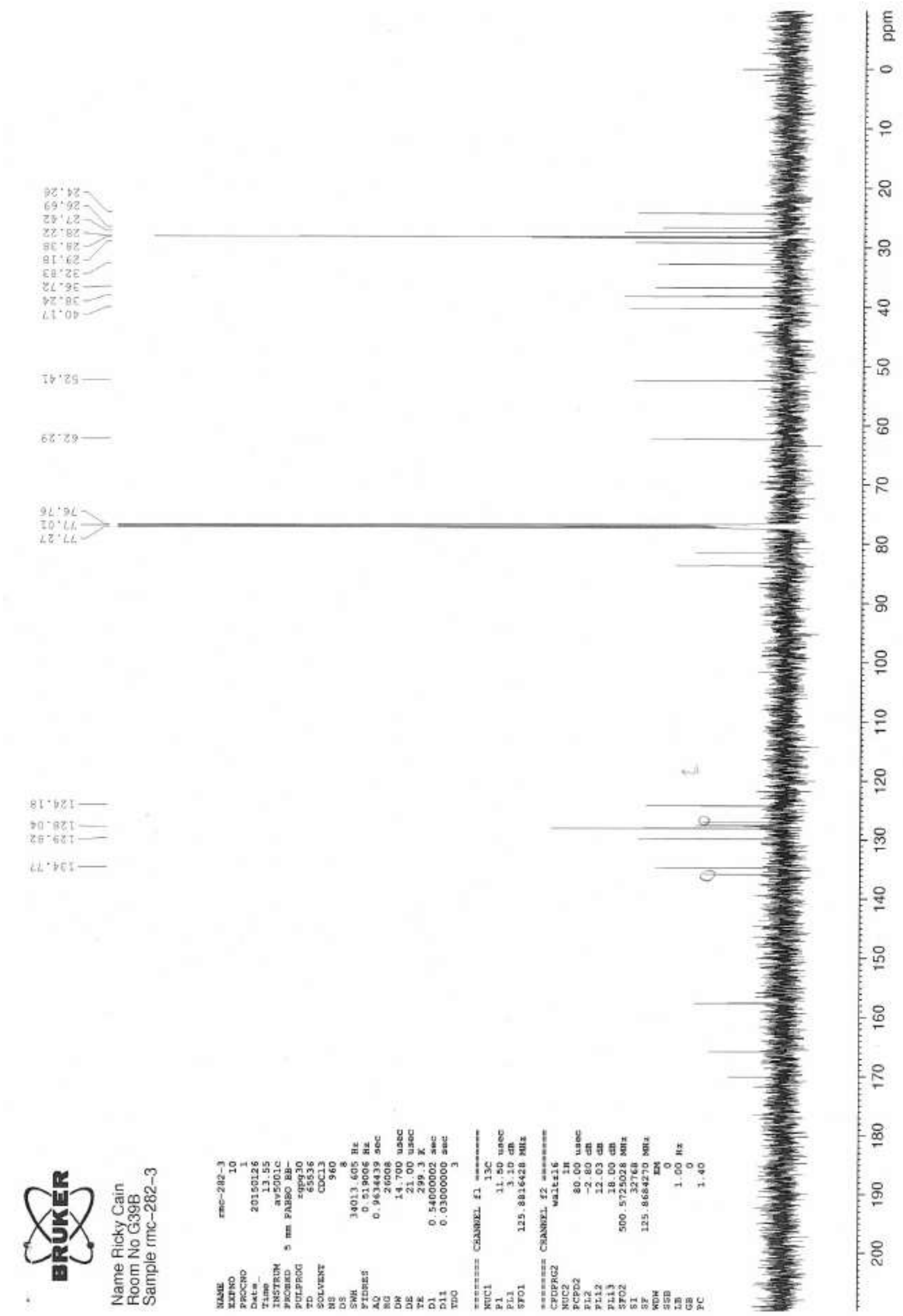

Supplementary Fig. 39. ^{13}C NMR spectrum of S9b.

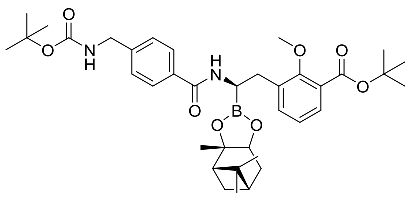

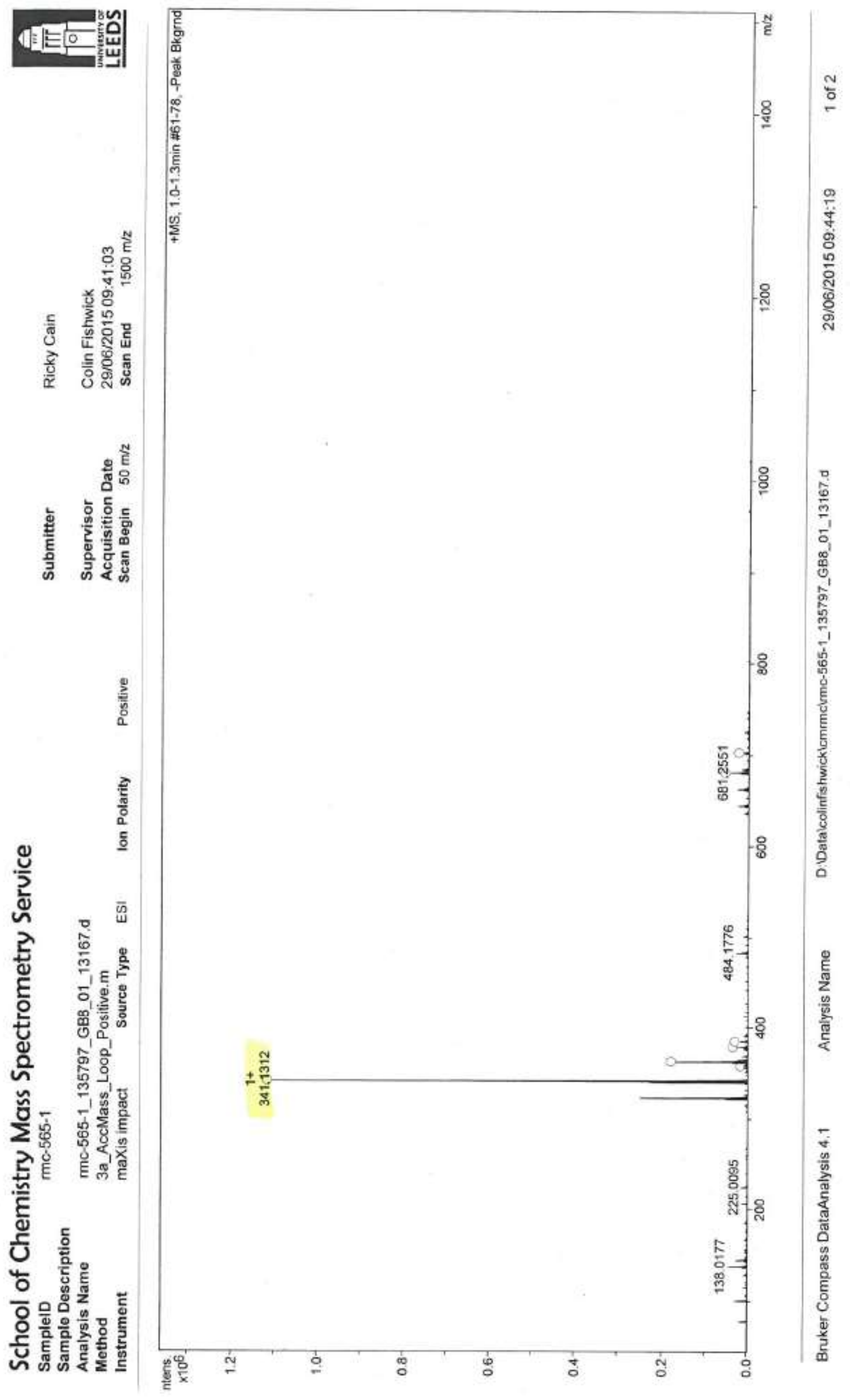

Supplementary Fig. 40. MS spectrum of 2.

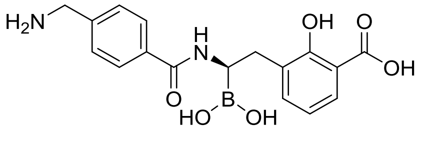

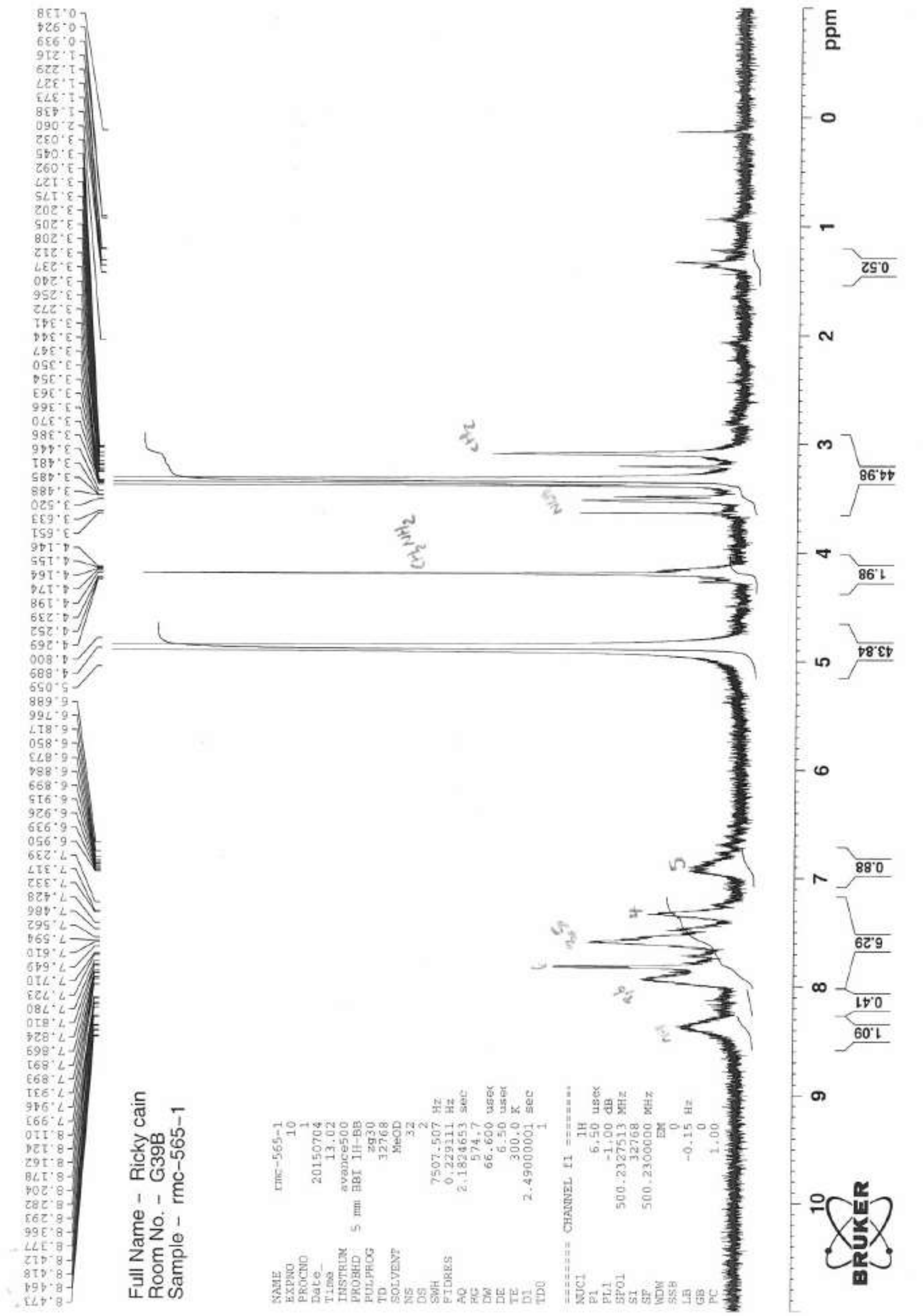

Supplementary Fig. 41. ^{1}H NMR spectrum of 2.

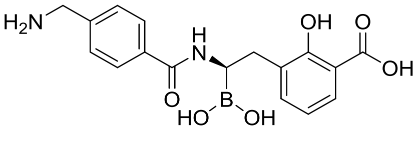

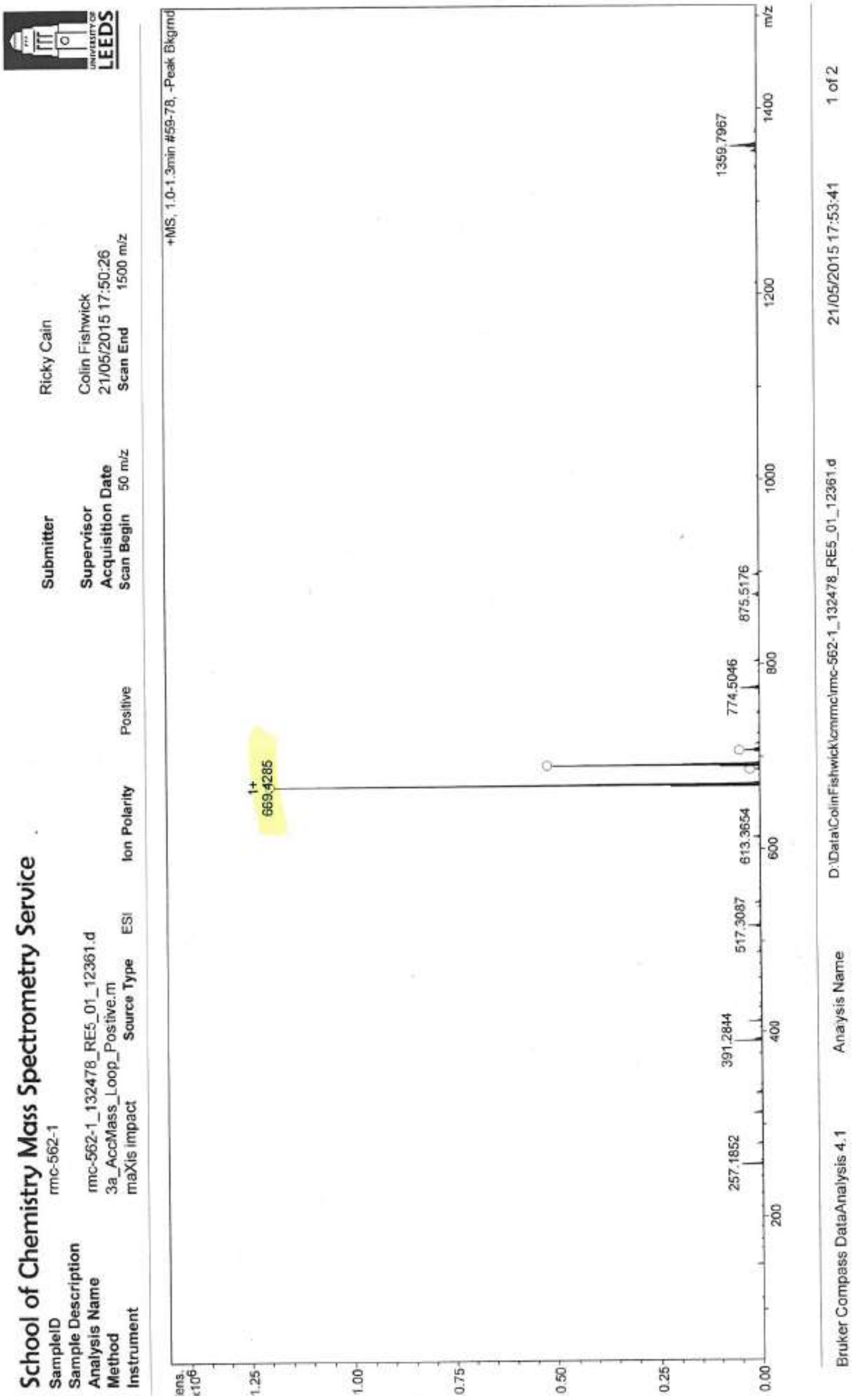

Supplementary Fig. 42. MS spectrum of S9c.

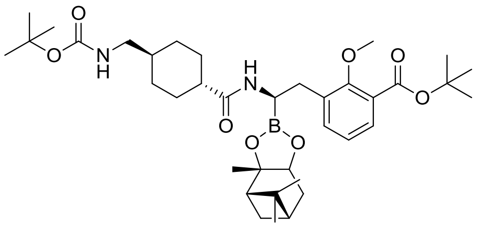

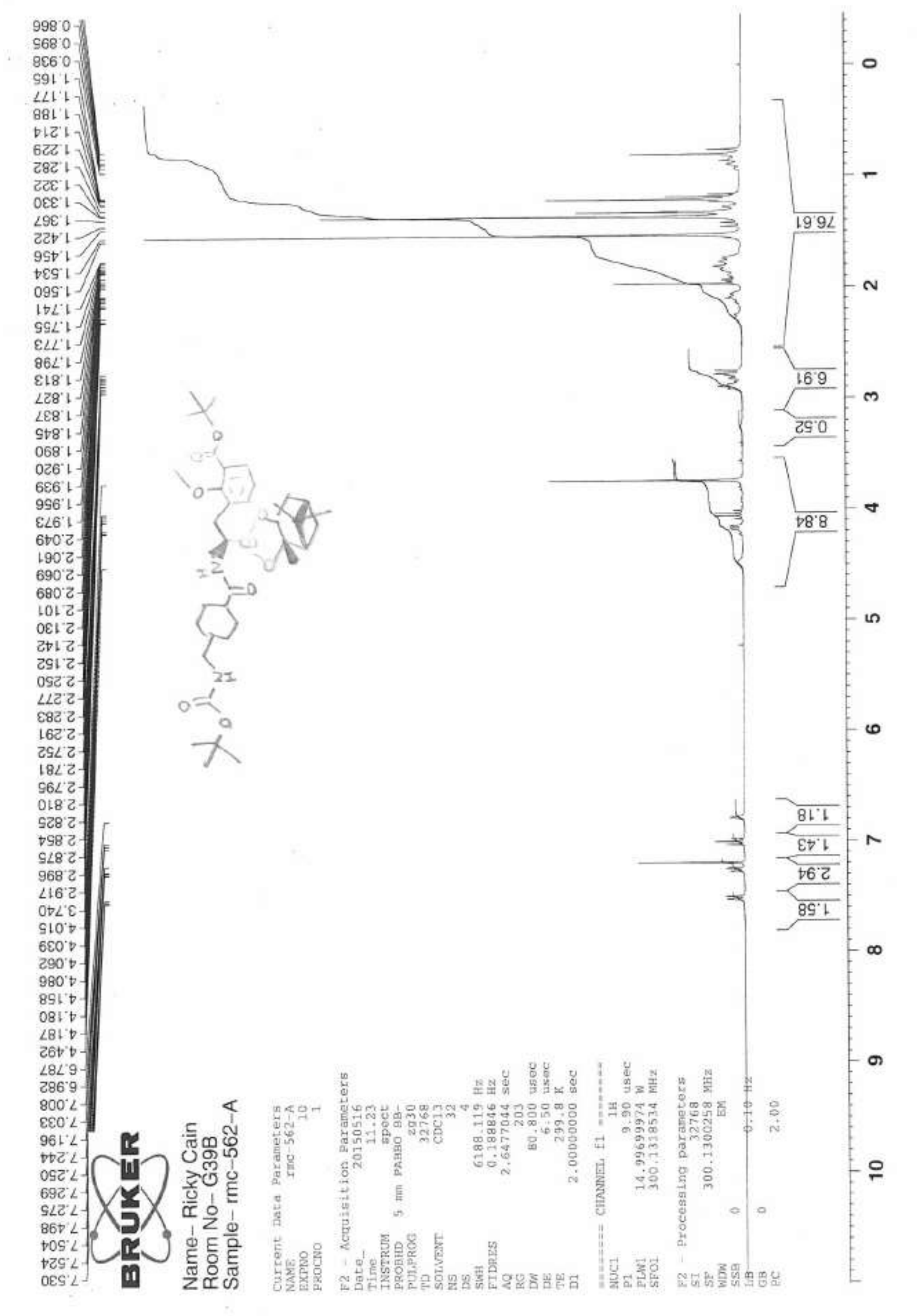

Supplementary Fig. 43. ^{1}H NMR spectrum of S9c.

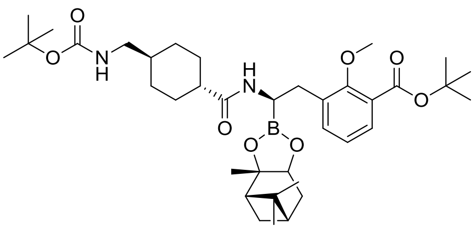

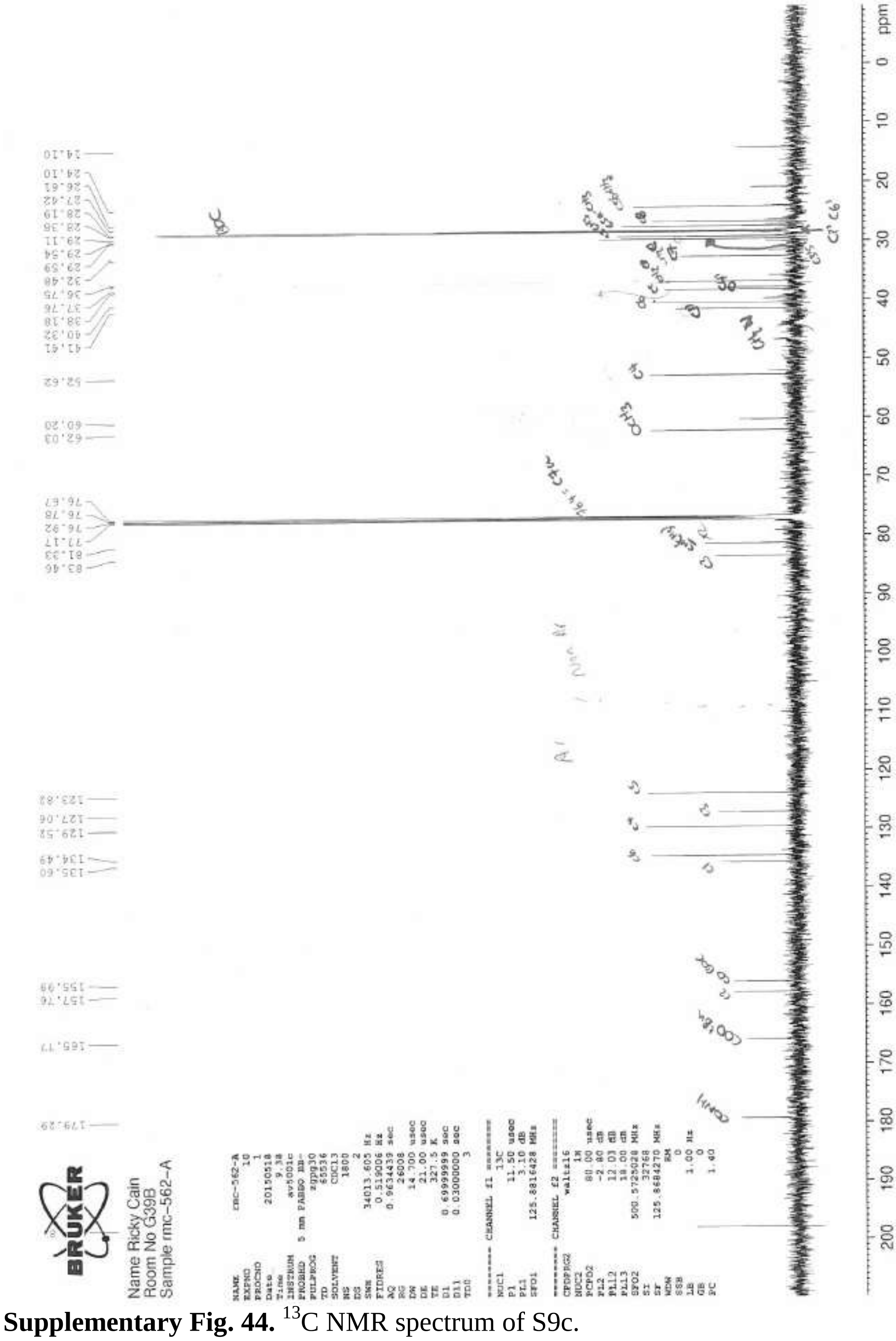

Supplementary Fig. 44. ^{13}C NMR spectrum of S9c.

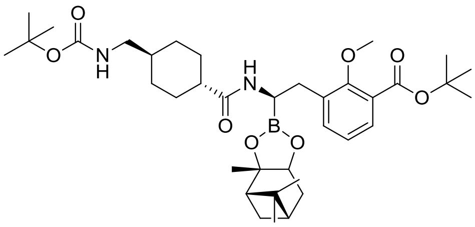

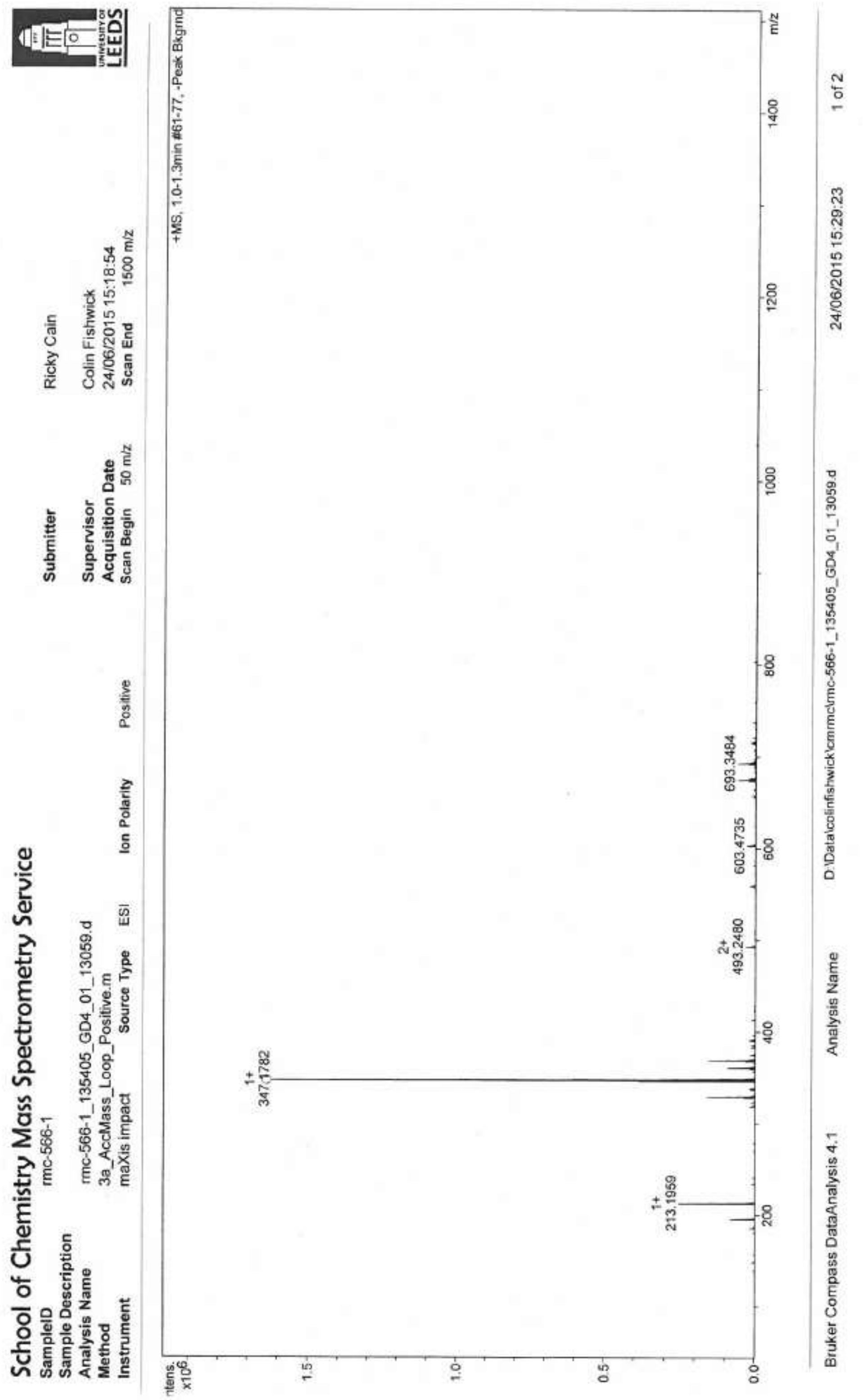

Supplementary Fig. 45. MS spectrum of 3.

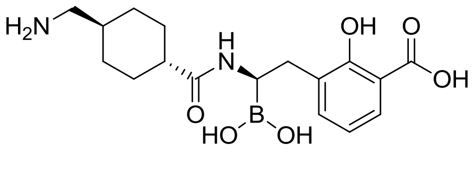

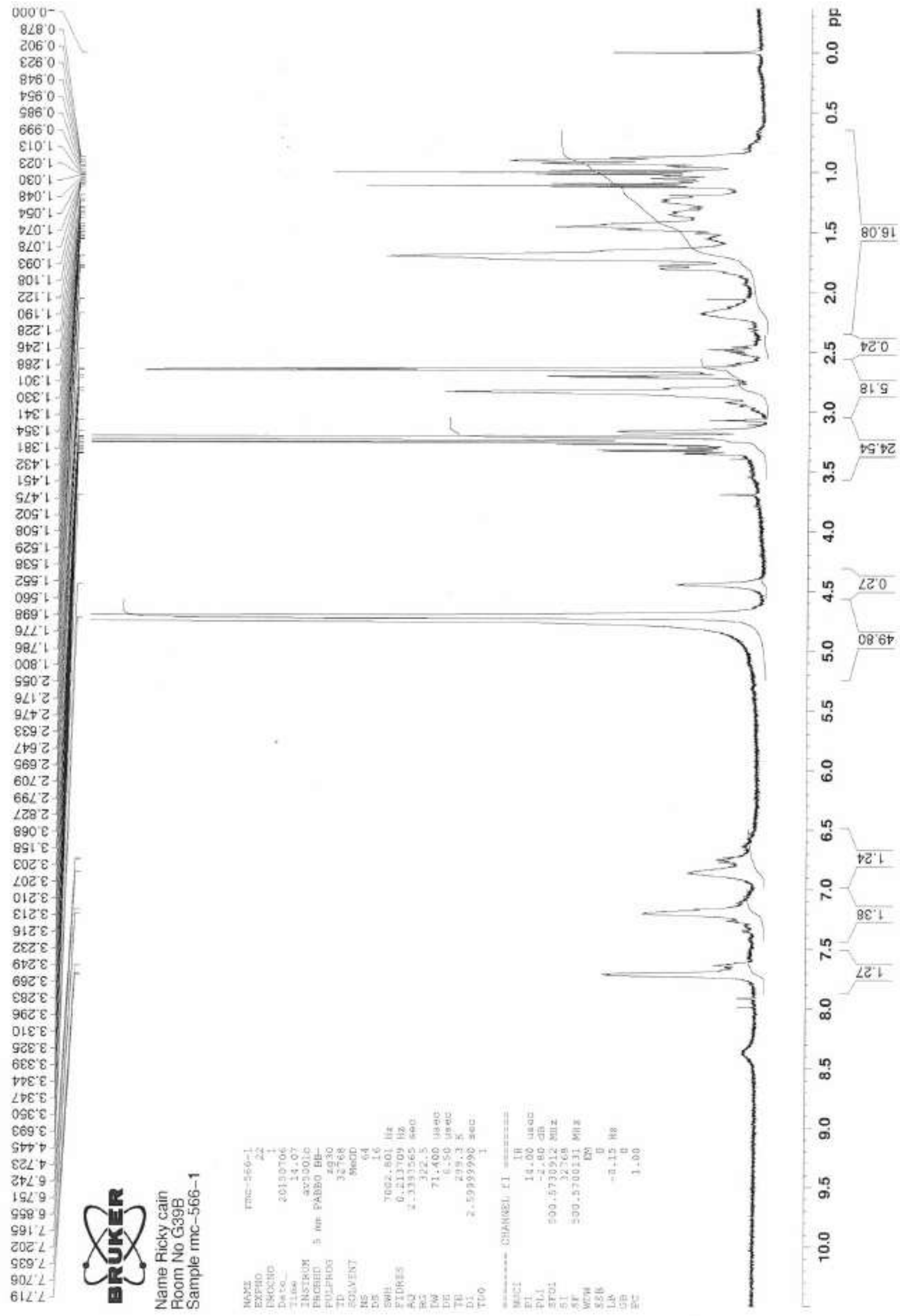

Supplementary Fig. 46. ^{1}H NMR spectrum of 3.

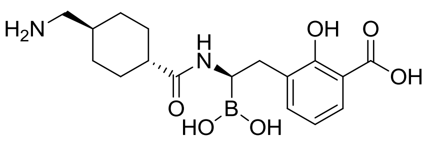

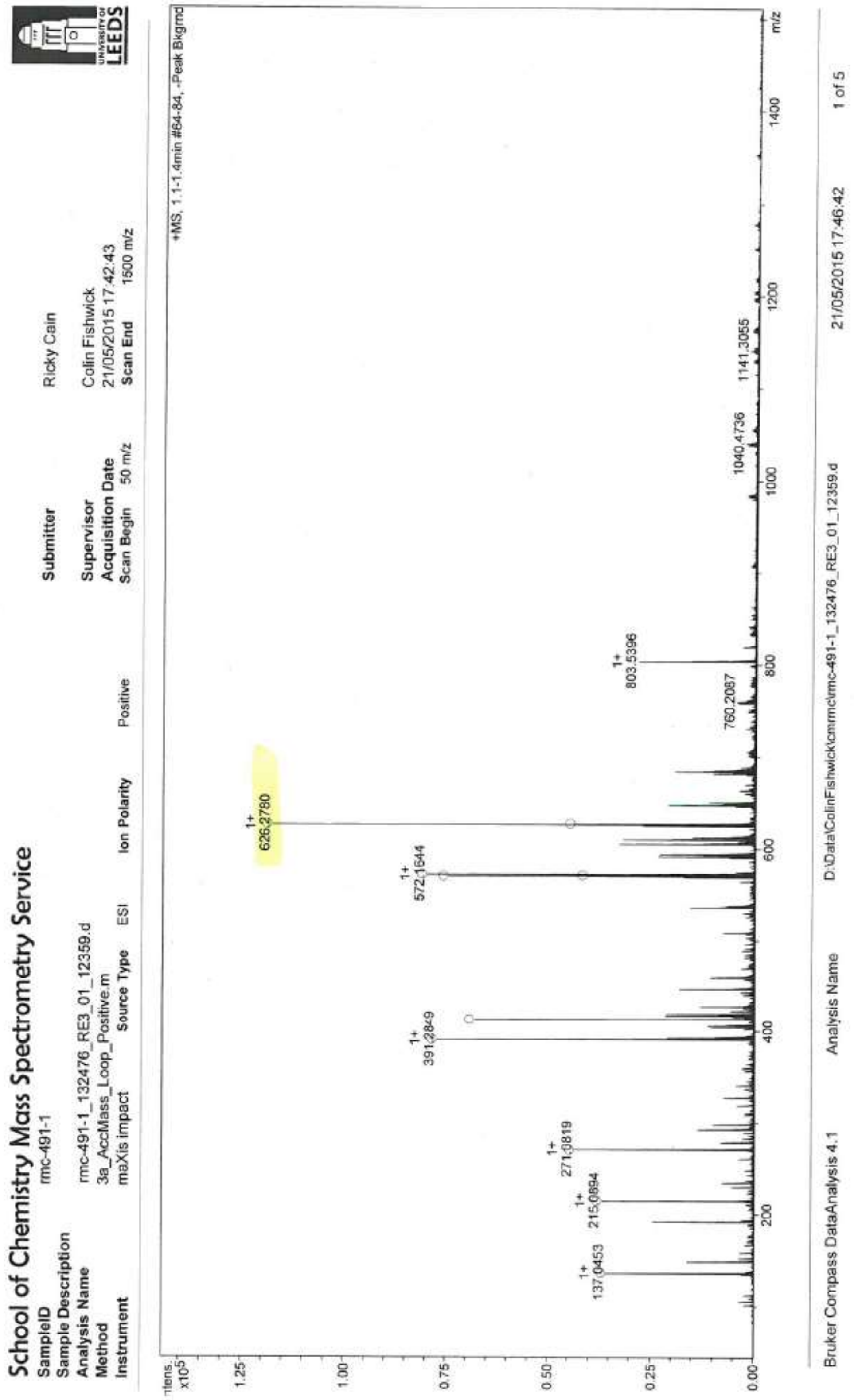

Supplementary Fig. 47. MS spectrum of S9d.

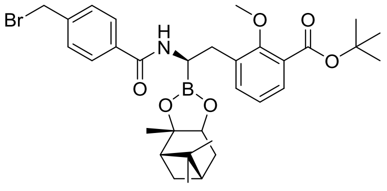

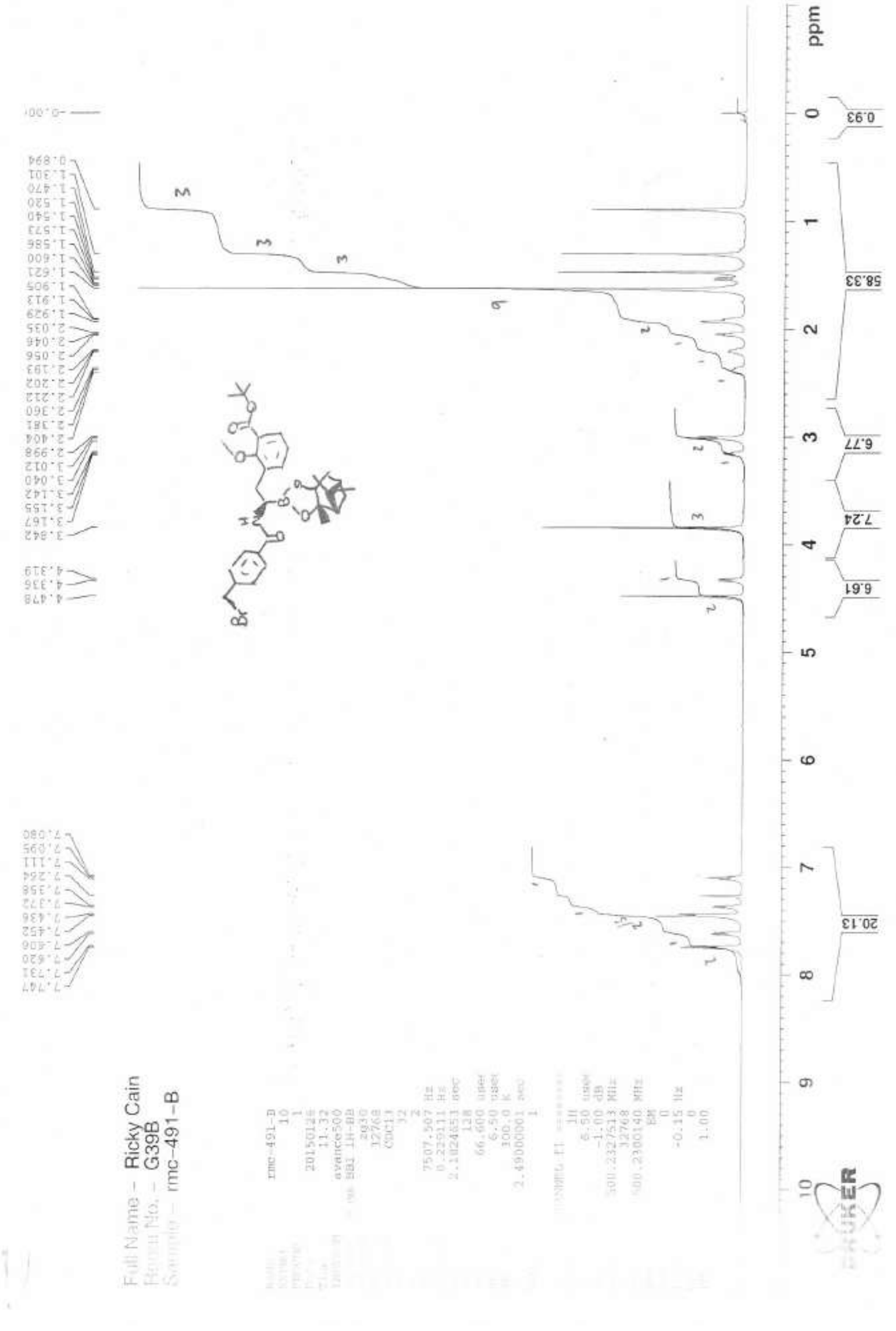

Supplementary Fig. 48. ^{1}H NMR spectrum of S9d.

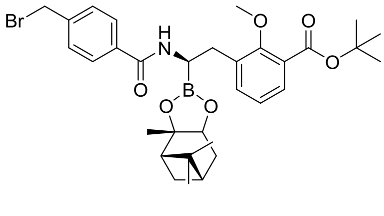

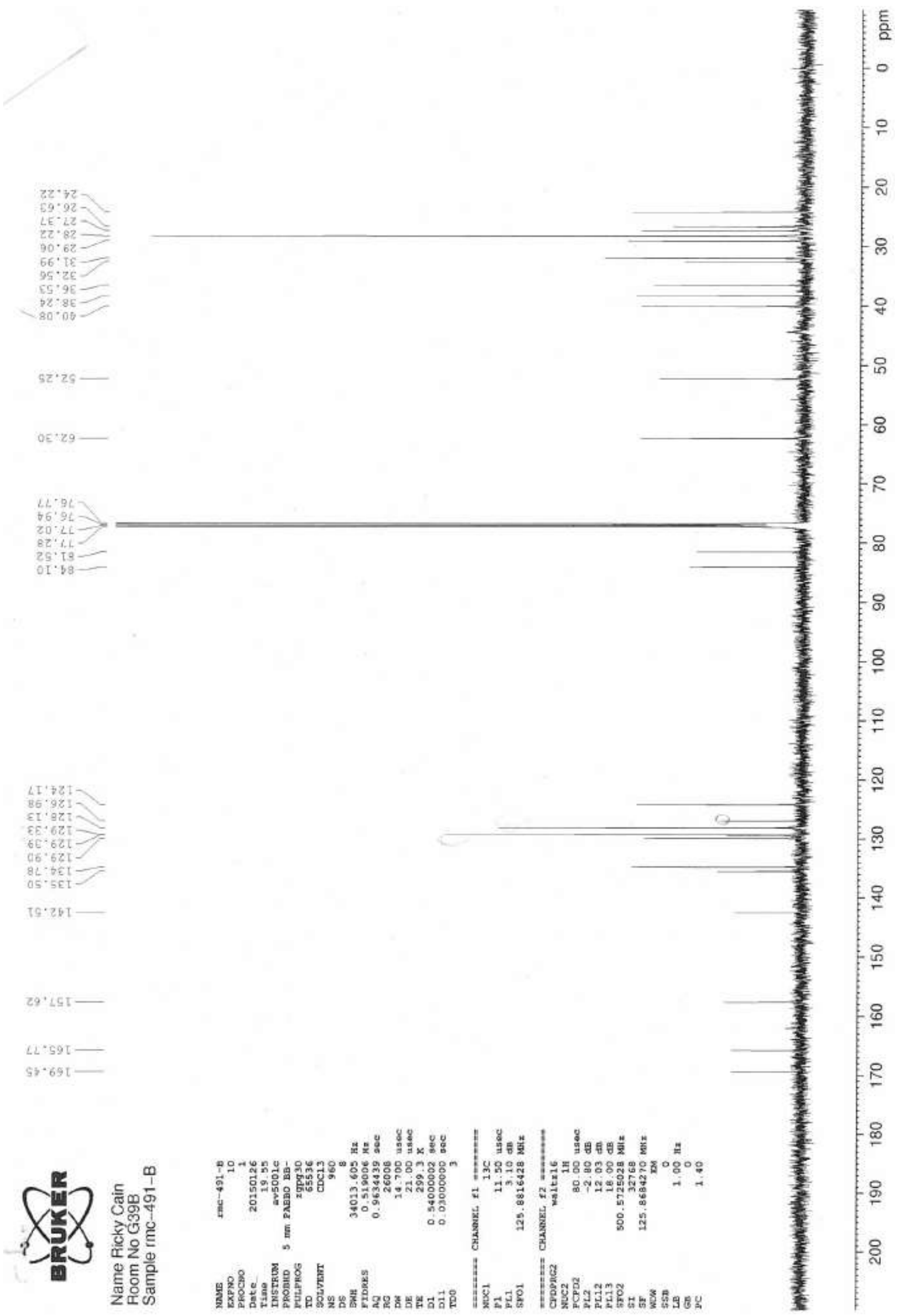

Supplementary Fig. 49. ^{13}C NMR spectrum of S9d.

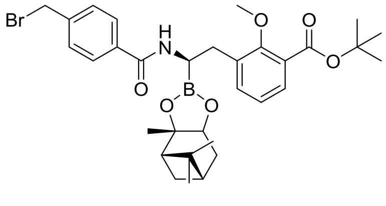

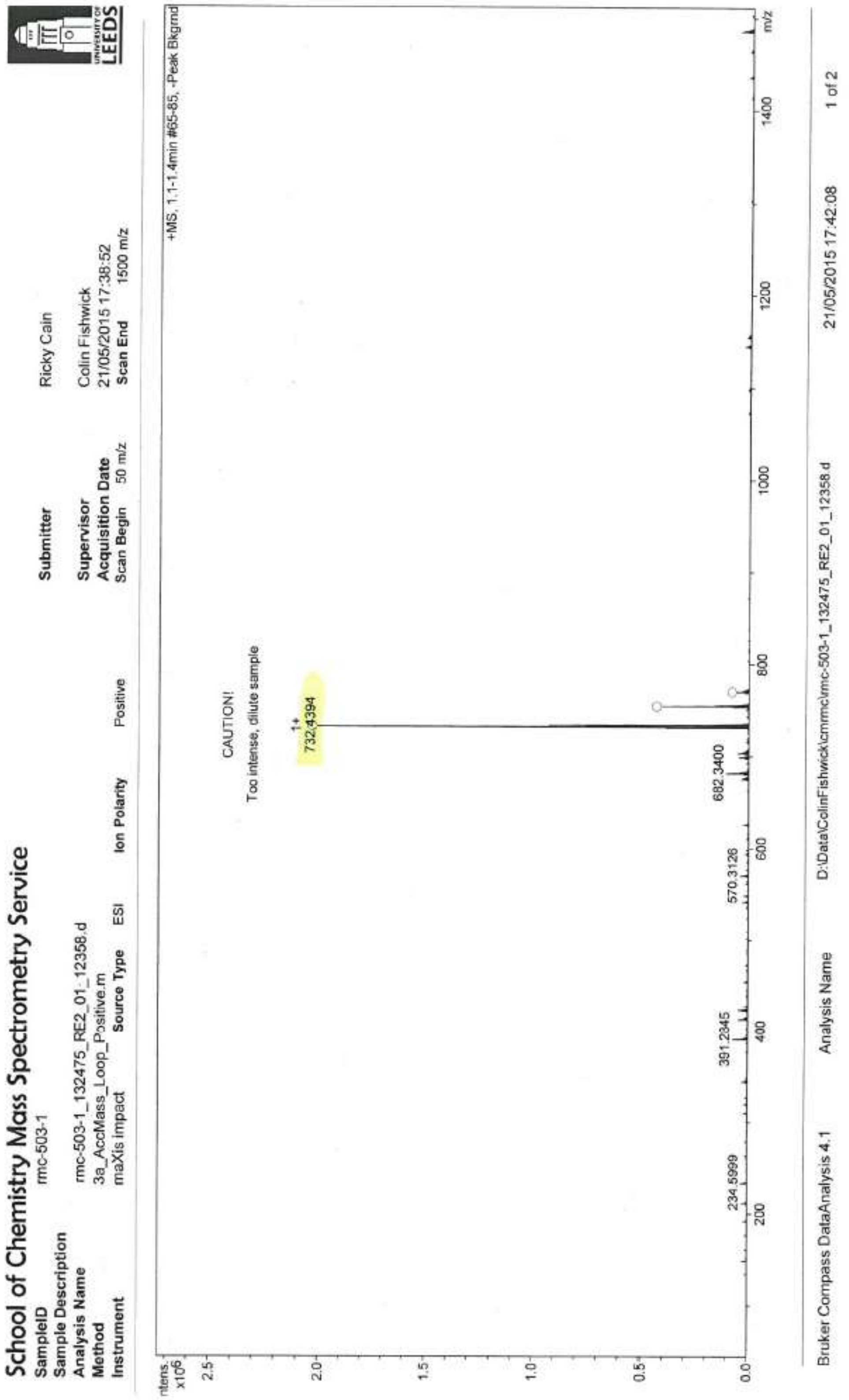

Supplementary Fig. 50. MS spectrum of S10a.

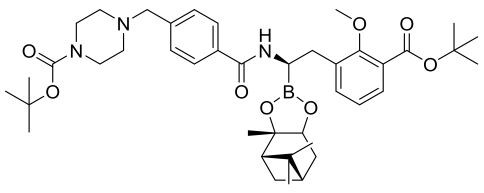

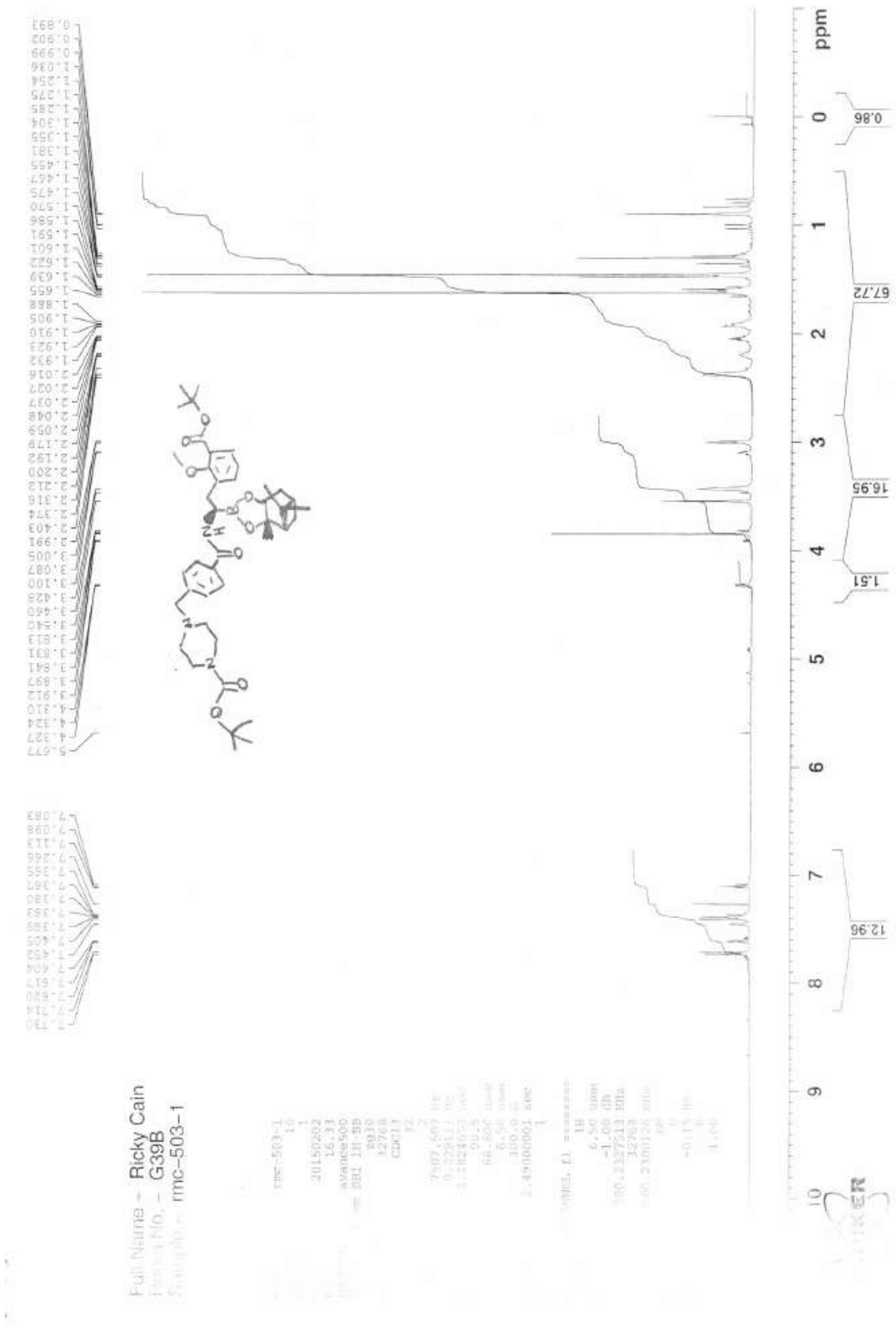

Supplementary Fig. 51. ^{1}H NMR spectrum of S10a.

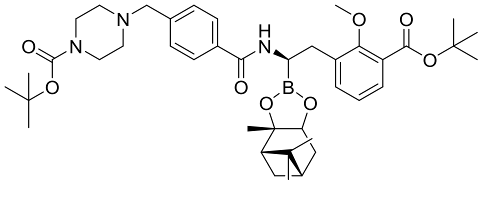

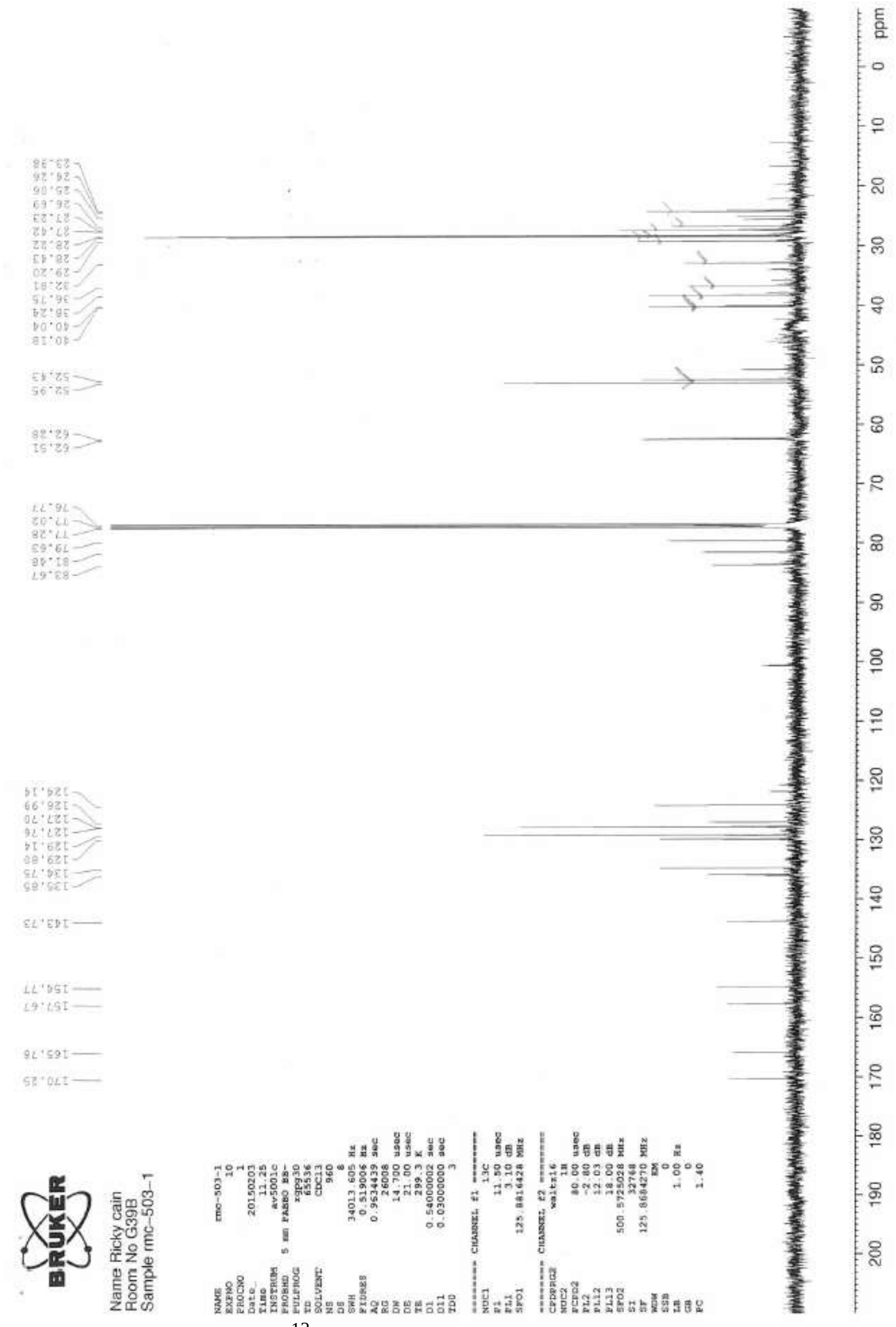

Supplementary Fig. 52. ^{13}C NMR spectrum of S10a.

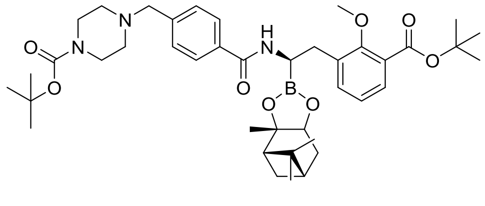

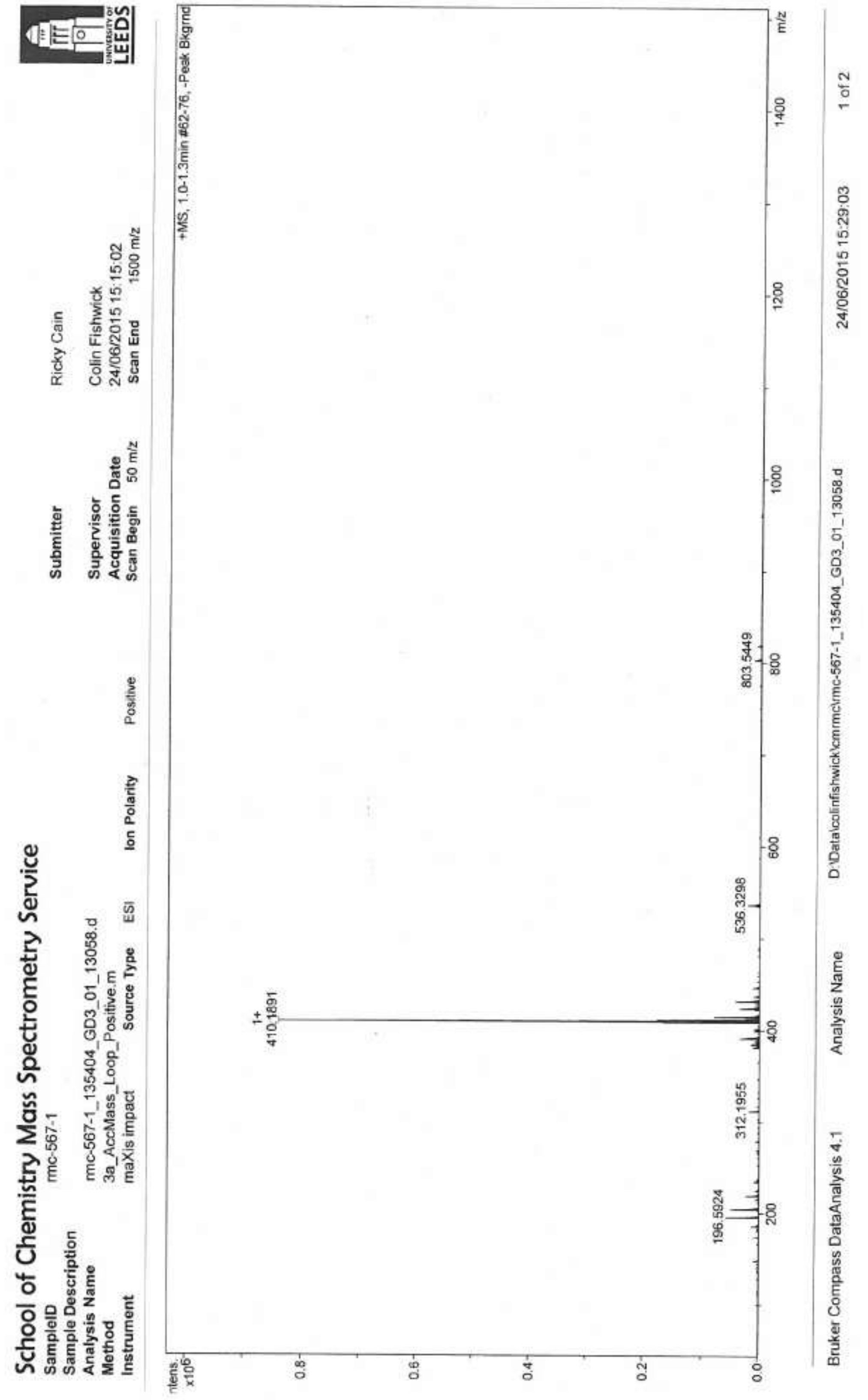

Supplementary Fig. 53. MS spectrum of 4.

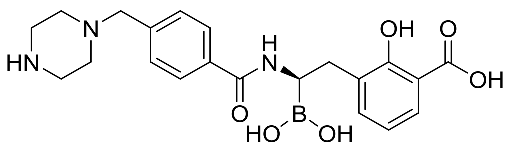

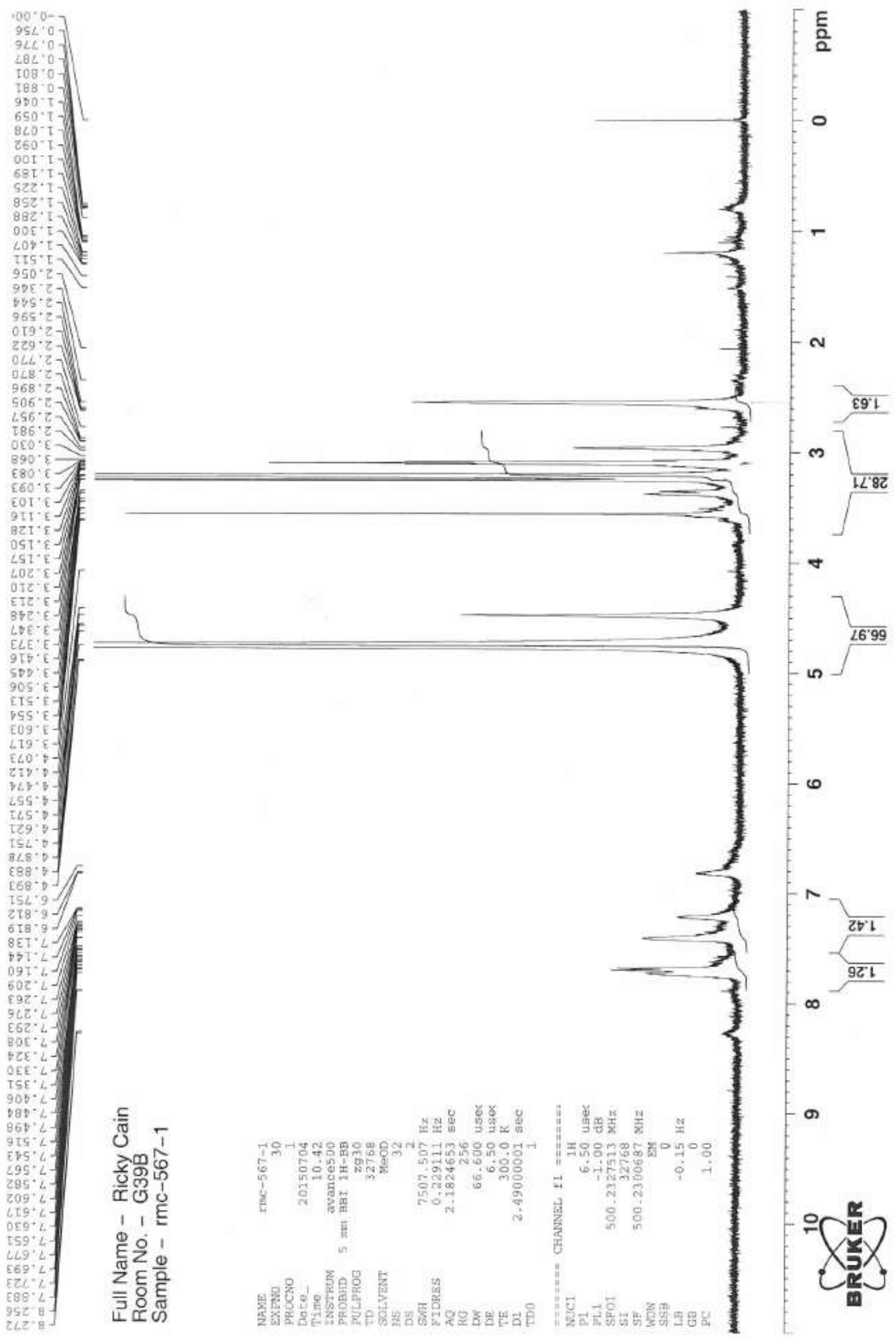

Supplementary Fig. 54. ^{1}H NMR spectrum of 4.

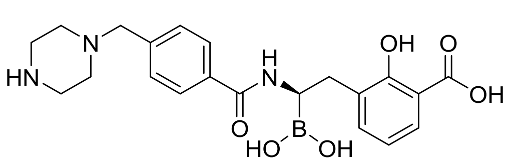

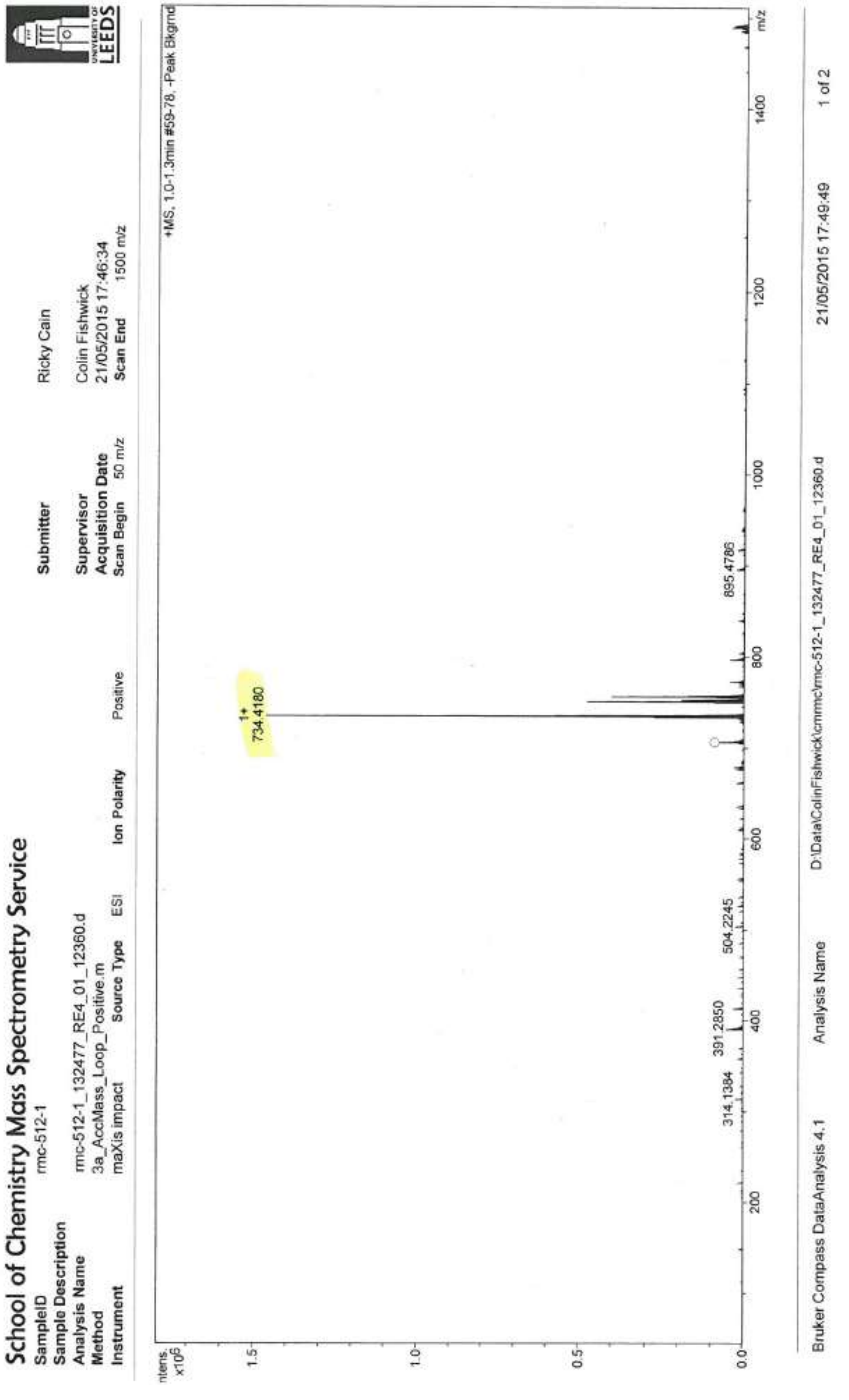

Supplementary Fig. 55. MS spectrum of S10b.

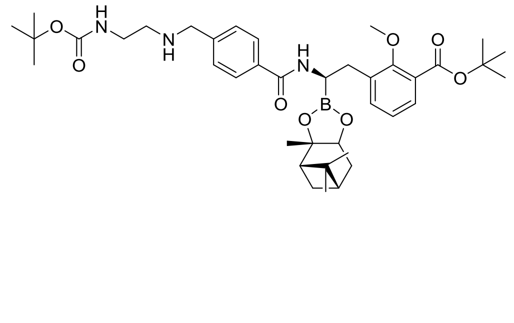

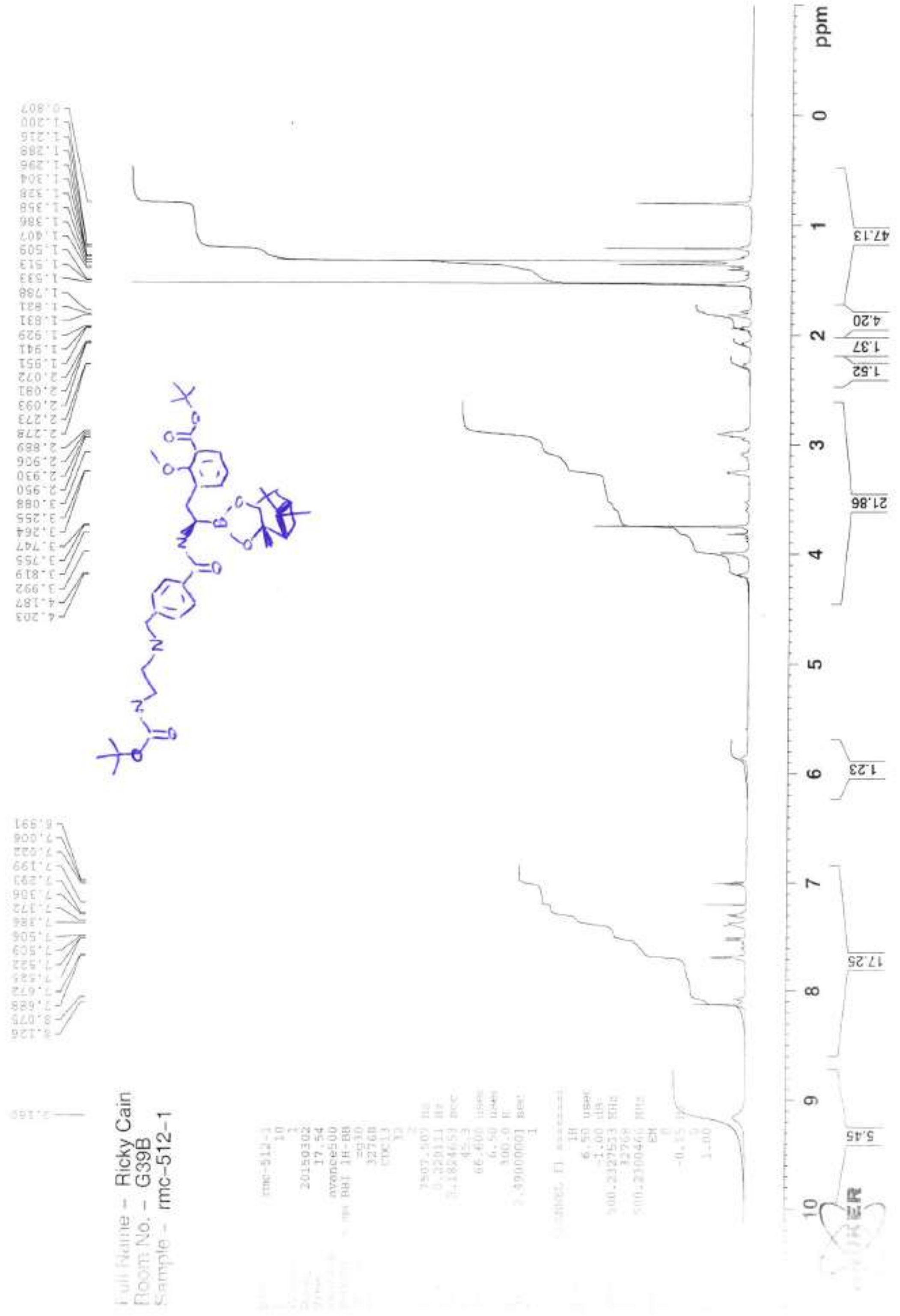

Supplementary Fig. 56. ^{1}H NMR spectrum of S10b.

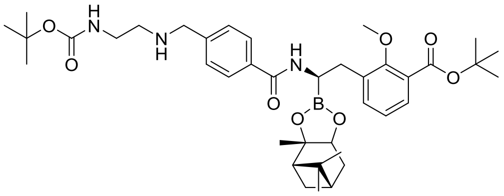

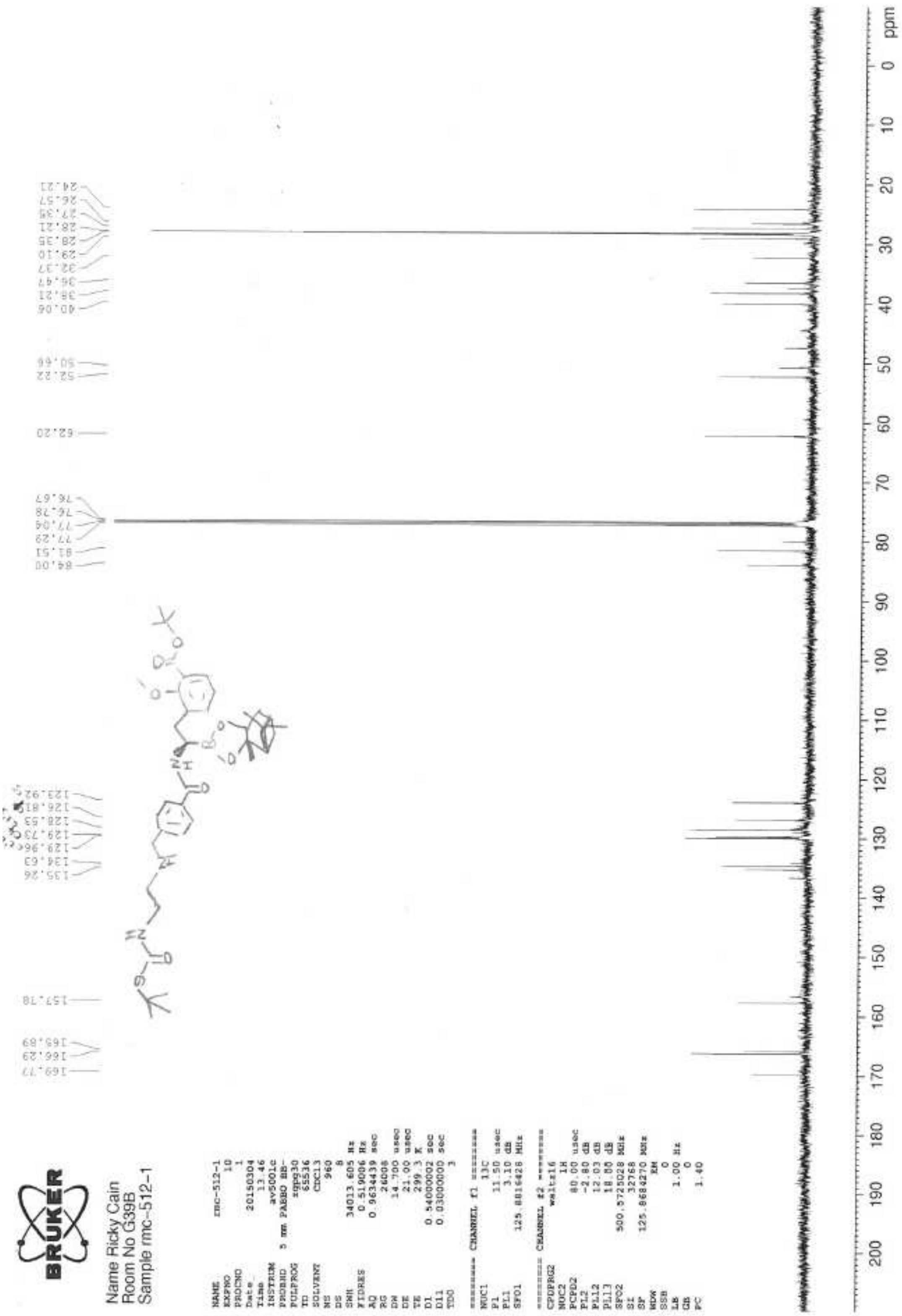

Supplementary Fig. 57. ^{13}C NMR spectrum of S10b.

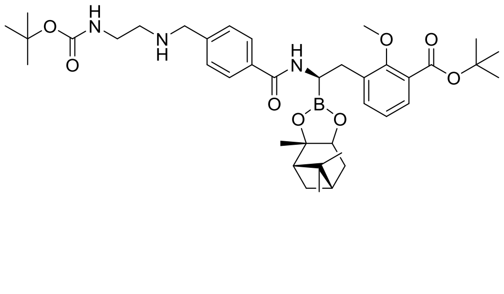

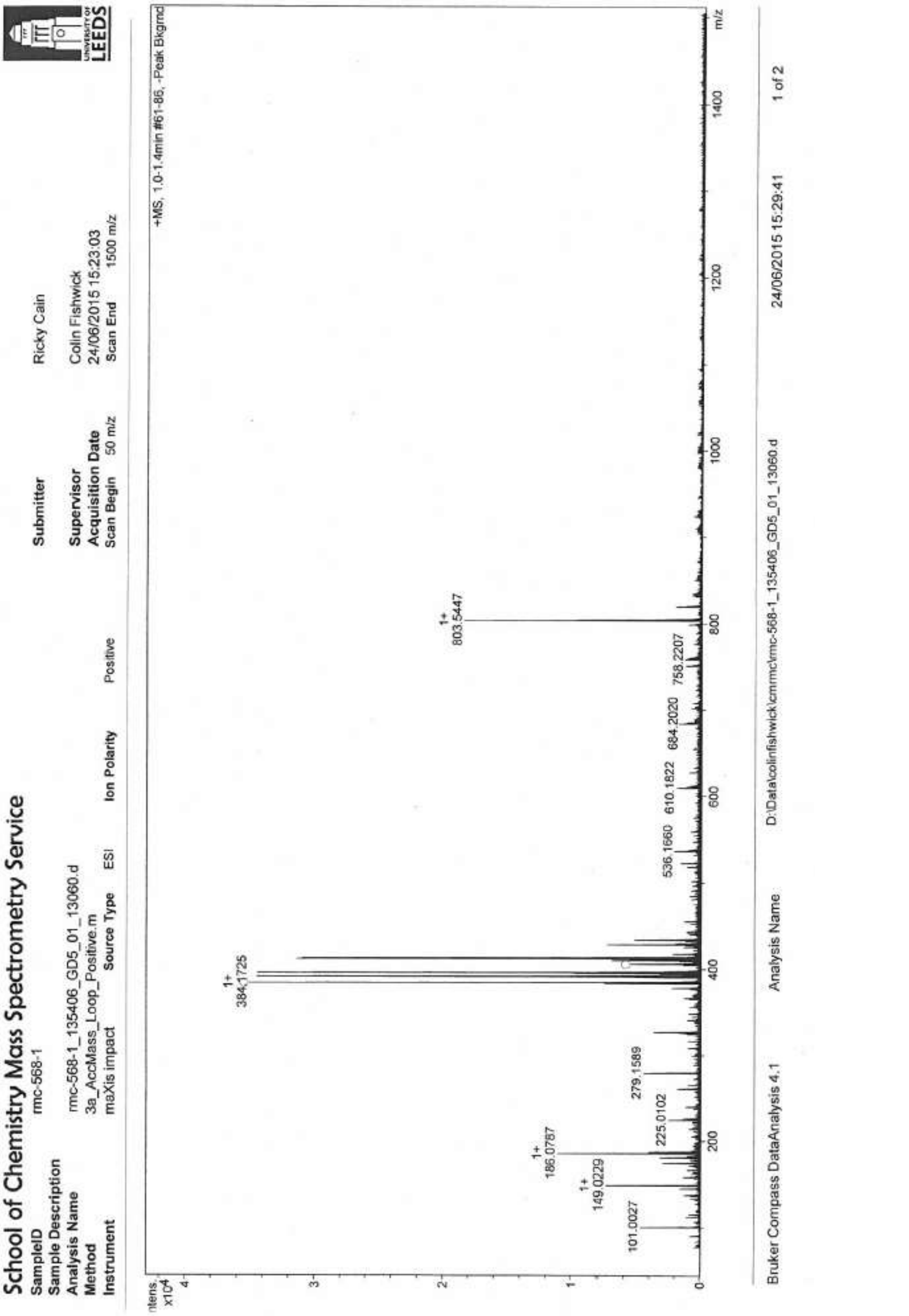

Supplementary Fig. 58. MS spectrum of 5.

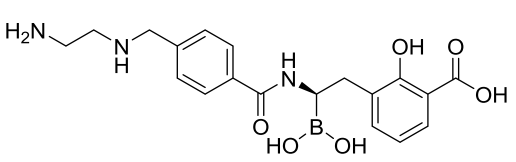

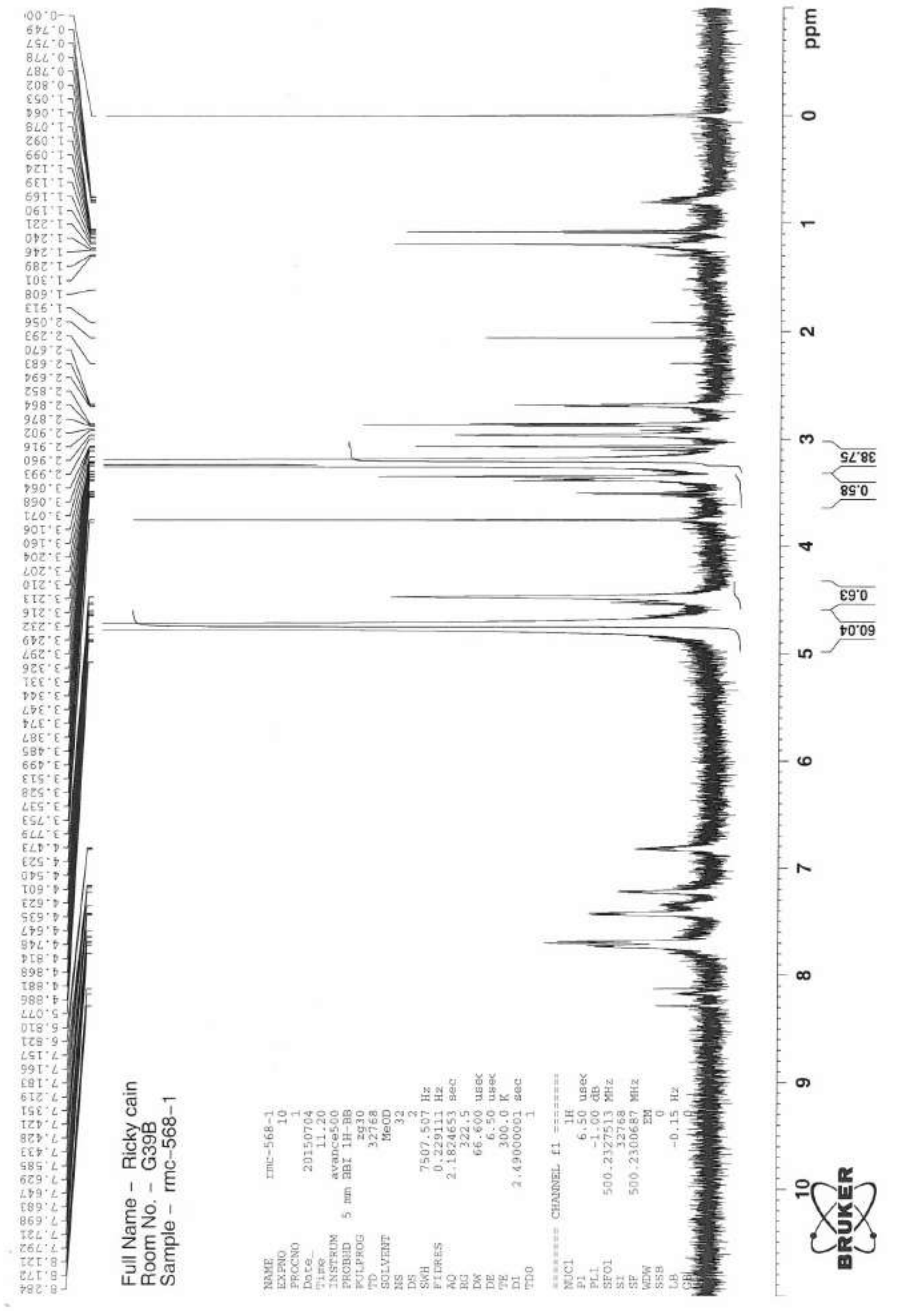

Supplementary Fig. 59. ^{1}H NMR spectrum of 5.

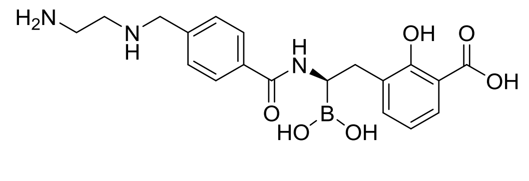

Supplementary Tables

Supplementary Table 1. In vitro cell based screening of cyclic boronate 2. Checkerboard experiment with *K. pneumoniae* Ecl8:pSU18:NDM-1.

**2 (μg mL^{-1})**	**0**	**0.5**	**1**	**2**	**4**	**8**	**16**	**32**	**64**
**Meropenem MIC (μg mL^{-1})**	**128**	**64**	**32**	**32**	**16**	**8**	**4**	**2**	**≤1**

Supplementary Table 2. Crystallisation conditions.

	Sample composition	Crystallisation conditions	Vapour diffusion conditions
BcII:**2**	BcII in crystallization buffer, TCEP (1 mM), **2** (12.5 mM)	0.2 M ammonium sulphate, 0.1 M Bis-Tris pH 5.5, 25 % PEG 3350	Sitting drop, protein-to-well ratio, 2:1, 293K
VIM-2:**2**	VIM-2 in crystallization buffer, TCEP (1 mM), **2** (12.5 mM)	0.12 M magnesium formate, 25% PEG 3350	Sitting drop, protein-to-well ratio, 1:1, 293K
OXA-10:**1**	OXA-10 in crystallization buffer, **1** (10 mM)	0.2 M NaCl, 0.1 M Na acetate pH 5.0, 20 % PEG 6000	Sitting drop, protein-to-well ratio, 1:1, 293K
PBP 5:**2**	PBP 5 in crystallization buffer, **2** (12.5 mM)	0.1 M HEPES sodium pH 7.5, 10 % v/v 2-Propanol, 20% PEG 4000	Sitting drop, protein-to-well ratio, 1:1, 293K

Supplementary Table 3. Data collection and refinement statistics.

**Structure**	**BcII−2**	**VIM-2−2**	**OXA-10−1**	**PBP 5−2**
PDB ID	5FQB	5FQC	5FQ9	5J8X
Radiation Source	Rotating anode	Diamond I04	Diamond I04	Diamond I02
Resolution Range (Å)	21.64 - 1.90 (1.97 - 1.90)‡	28.89 - 1.45 (1.50 - 1.45)‡	15.42 - 1.50 (1.55 - 1.50)‡	28.14 - 2.52 (2.61 - 2.52)
Space group	$C\ 2_1$	$C\ 2_1$	$P\ 2_1\ 2_1\ 2_1$	$P\ 2\ 2_1\ 2_1$
Unit cell dimensions				
a,b,c (Å)	53.14 61.11 69.39	101.93 79.15 67.13	48.89 103.10 125.52	50.22 61.60 138.43
α,β,γ (°)	90 93.12 90	90 130.16 90	90 90 90	90 90 90
No. of molecules/ASU	1	2	2	1
No. of unique reflections	17335(1684)‡	71720 (7077) ‡	102231 (10087) ‡	14957 (1427)‡
Completeness (%)	98.38 (95.43)‡	99.4 (97)‡	99.9 (99.5)‡	99.9 (99.7) ‡
Redundancy	3.7 (3.5)‡	4.4 (3.7)‡	5.9 (8.9)‡	6.4 (5.3) ‡
R_{merge}	0.058 (0.088)‡	0.065 (0.582)‡	0.047 (1.1)‡	0.098 (0.593)‡
Mean I/σ(I)	17.8 (5.2)‡	11 (1.8)‡	5.6 (2.0)‡	13.5 (1.8) ‡
Wilson B value (Å^2)	20.69	14.52	16.33	46.34
**Refinement**				
R_{factor}	0.1403 (0.1562) ‡	0.1550 (0.2454) ‡	0.1569 (0.2987) ‡	0.2000 (0.2873) ‡
R_{free}	0.1807 (0.2044) ‡	0.1852 (0.2715) ‡	0.1806 (0.3129) ‡	0.2334 (0.3465) ‡
R.m.s.d.				
Bond length (Å)	0.010	0.014	0.014	0.003
Bond angle (°)	1.25	1.41	1.34	0.56
Total no. of atoms	1914	4090	4494	2597
Protein	1685	3569	3927	2547
Ligand/ion	39	74	76	26
Water	190	447	491	26
$\langle B_{factor} \rangle$ (Å^2)				
Protein	26.80	17.85	27.40	54.19
Ligand/ion	33.50	19.25	36.06	54.22
Water	34.70	30.90	37.30	51.58

R_{merge} is the unweighted R-value on I between merged reflections.

$R_{factor} = \Sigma_{hkl}$ | $|F_{obs}$ (hkl)| - $|F_{calc}$ (hkl)| | / Σ_{hkl} $|F_{obs}$ (hkl)| for the working set of reflections.

‡ outermost shell.

Supplementary Methods

Chemicals were from commonly used suppliers and used without further purification. Solvents (including dry solvents) for chemical transformations, work-up and chromatography were from Sigma-Aldrich (Dorset, UK) at HPLC grade, and were used without further distillation. Silica gel 60 F254 analytical thin layer chromatography (TLC) plates were from Merck (Darmstadt, Germany) and visualized under UV light and/or with potassium permanganate stain. Chromatographic purifications were performed using Merck Geduran 60 silica (40-63 μm) or prepacked SNAP columns on a Biotage Isolera Purification system (Uppsala, Sweden). Deuterated solvents were from Sigma-Aldrich, Chambridge Isotopes, and Apollo Scientific Ltd. All ^{1}H and ^{13}C NMR spectra were recorded using a Bruker Avance 500 MHz spectrometer. All chemical shifts are in ppm relative to the solvent peak, and coupling constants (J) are reported in Hz to the nearest 0.5. High Resolution (HR) mass spectrometry data (m/z) were obtained using a Bruker MaXis Impact instrument with an ESI source and Time of Flight (TOF) analyzer. Fourier transform Infrared (FT-IR) spectra were recorded on a Bruker Alpha Platinum instrument. Optical rotations were recorded on a Schmidt and Haensch H532 Polarimeter. Melting points were obtained from a Reichert Hot Stage melting point apparatus. HPLC analysis was run on an Agilent 1290 Infinity system equipped with a Supelco Ascentis Express 2.7 μM C18 column (50 x 2.1 mm) using a gradient of 95% solvent A → 95% solvent B (solvent A: H_2O containing 0.1% formic acid; solvent B: 100% MeCN containing 0.1% formic acid), flow rate = 0.5 mL min^{-1} and UV detection at 254 nm. Preparative HPLC was run on an Agilent mass directed preparative LCMS equipped with a XBridge 5.0 μM C18 column (100 x 19 mm) using a gradient of 95% solvent A → 95% solvent B (solvent A: H_2O containing 0.1% formic acid; solvent B: 100% MeCN containing 0.1% formic acid), flow rate = 20 mL min^{-1} and UV detection at 254 nm.

Supplementary Synthesis

Synthetic Routes

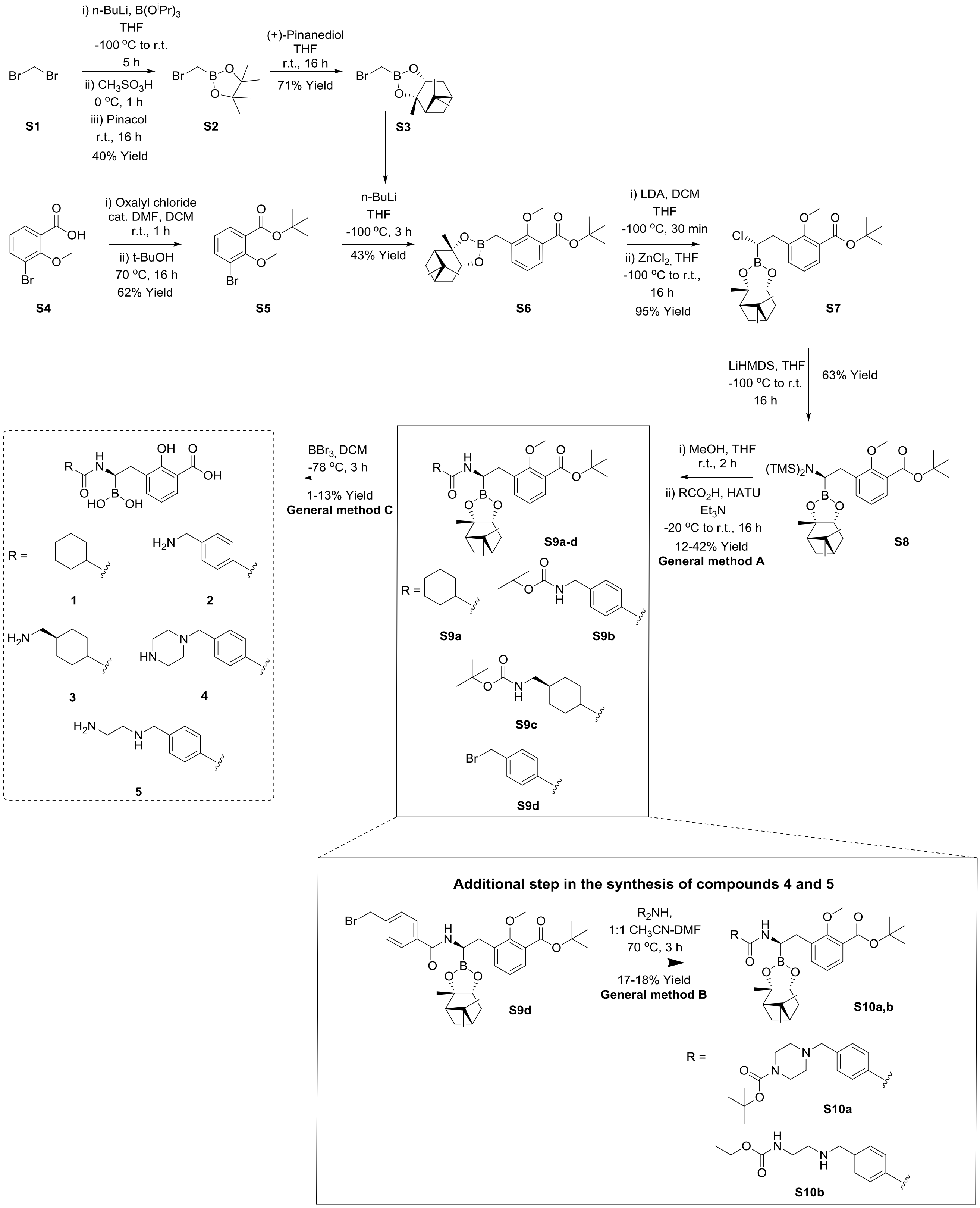

General method A: Trimethylsilyl (TMS) group deprotection and amide coupling

The desired TMS protected amine (1.2 eq) was dissolved in THF (20 mL); to this solution was added anhydrous MeOH (2 mL). The reaction mixture was stirred at room temperature for 2 h. The reaction mixture was concentrated *in vacuo* to afford a pale brown oil which was used in the next step without further purification.

The desired carboxylic acid (1.0 eq) and HATU (1.1 eq) were dissolved in CH_2Cl_2 (DCM) (30 mL). The reaction mixture was cooled to 0 °C, then Et_3N (1.5 eq) was added dropwise. The reaction mixture was stirred at 0 °C for 30 mins and then at room temperature for 1 h. The solution was then cooled to −20 °C and the desired amine (1.2-2.0 eq) added dropwise. The reaction mixture was allowed to warm to room temperature overnight. After this time the reaction was quenched with water (50 mL). The aqueous layer was extracted with EtOAc (3 × 50 mL). The combined organics were dried ($MgSO_4$) and concentrated *in vacuo* to give the desired amide, which was purified using flash column chromatography eluting with Hexane/EtOAc to afford the coupled products.

General method B: S_N2 reaction introducing terminal amine functionality in compounds 4 and 5

3-[(2*R*)-2-[4-(bromomethyl)-benzoylamino]-2-[(3a*S*,4*S*,6*S*,7a*R*)-3a,5,5-trimethylhexahydro-4,6-methano-1,3,2-benzodioxaborol-2-yl]-ethyl]-2-methoxy benzoic acid tert-butyl ester (S9d) (1.0 eq) was dissolved in CH_3CN:DMF (1:1, 10 mL); to this mixture was added the desired amine (1.0 eq). Na_2CO_3 (1.1 eq) was added and the solution heated to 70 °C for 3 h. The reaction was cooled to room temperature and extracted with EtOAc (3 × 20 mL). The organics were washed with water (3 × 20 mL) and brine (20 mL), dried ($MgSO_4$) and concentrated *in vacuo* to afford the crude product. The crude product was purified by preparative HPLC to afford the desired product as an oil.

General method C: Global deprotection to give cyclic boronate inhibitors.

Boron tribromide (1M in DCM, 7.0 eq) was added drop-wise to a solution of the boronic acid derivative (1.0 eq) in DCM (10 mL) at −78 °C. The reaction mixture was stirred at −78 °C for 1 h before warming to room temperature and quenched with water (6 mL). The DCM layer was evaporated *in situ.* Water (75 mL) was then added and the mixture was extracted with diethyl ether (3 × 50 mL). The aqueous layer was concentrated to ~5 mL and the pH adjusted to ~pH1 with 2M HCl $_{(aq)}$. The product was purified by biotage

chromatography on a C_{18} reverse phase cartridge eluting with 2:98 Isopropanol-water to afford the desired product as a colourless solid.

Synthesis of 2-(bromomethyl)-4,4,5,5-tetramethyl-1,3,2-dioxaborolane[9] (S2)

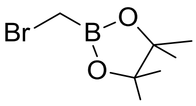

To a mixture of triisopropyl borate (20.0g, 110 mmol), dibromomethane (S1) (8.60 mL, 120 mmol) and THF (150 mL) was added *n*-butyllithium (1.6 M *n*-hexane solution, 63.6 mL, 100 mmol) at −100 °C (external temperature) over 1.5 h. The reaction mixture was stirred at the same temperature for 1.5 h, and then stirred at room temperature for 2 h. After the mixture was then cooled at 0 °C (external temperature), methanesulfonic acid (6.50 mL, 100 mmol) was added; the mixture was stirred at room temperature for 1 h. The mixture was then cooled to 0 °C (external temperature), when pinacol (12.0 g, 100 mmol) was added; the mixture was then stirred at room temperature for 1 h. The solvent was removed *in vacuo*; the resulting residue was distilled under reduced pressure (80-82 °C, 40 mmHg), to obtain desired product (S2) as a colourless oil. (16.0g, 72.4 mmol, 72%) R_f: 0.40 (19:1 Petrol−EtOAc); δ_H (300 MHz, $CDCl_3$): 2.57 (2H, s, CH_2), 1.27 (12H, s, CH_3); δ_C (75 MHz, $CDCl_3$): 84.6 (CH_2), 24.7 (CH_3); ν_{max}/ cm^{-1} (oil): 2922, 1415, 1338, 1286, 1144, 1029; *m/z* (ES): (Found: $[M-Br]^+$, 141.0833. $C_7H_{14}BBrO_2$ requires *[M-Br]*, 141.0837).

Synthesis of (3a*S*,4*S*,6*S*,7a*R*)-2-(bromomethyl)-3a,5,5-trimethylhexahydro-4,6-methano-1,3,2-benzodioxaborole[10] (S3)

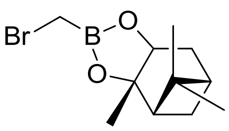

A mixture of 2-(bromomethyl)-4,4,5,5-tetramethyl-1,3,2 dioxaborolane (S2) (0.40 g, 1.18 mmol) and (+)-Pinanediol (0.62 g, 3.62 mmol) in THF (10 mL) was stirred at room temperature for 48 h. The reaction mixture was then concentrated *in vacuo*. The residue was partitioned between EtOAc (10 mL) and water (10 mL). The organic extracts were separated and the aqueous layer extracted with further EtOAc (2 × 10 mL). The combined organic extracts were dried ($MgSO_4$), then concentrated *in vacuo* to yield the crude product as a pale brown oil. The crude product was by flash column chromatography eluting with 3:1 Petrol–EtOAc. The appropriate fractions were collected, combined and concentrated to afford the desired product (S3) as a colourless oil which was used without further purification (0.29 g, 1.02 mmol, 47%) R_f: 0.50 (19:1 Petrol−EtOAc); δ_H (300 MHz, $CDCl_3$): 4.37 (1H, dd, *J* 9.0 and 2.0, 7a-H), 2.63 (2H, s, CH_2Br), 2.36 (1H, ddt, *J* 14.5, 9.0

and 2.0, 7*-H), 2.22-2.29 (1H, m, 8-H), 2.08 (1H, app. t, *J* 5.5, 4-H), 1.87-1.96 (2H, m, 7*-H and 6-H), 1.42 (3H, s, CH_3-C3a), 1.30 (3H, s, CH_3-5b), 1.20 (1H, d, *J* 11.0, 8-H), 0.85 (3H, s, CH_3-5a) ; δ_C (75 MHz, $CDCl_3$): 86.7 (C3a), 78.6 (C7), 51.2 (C4), 39.3 (C6) 38.2 (C5), 35.2 (C7), 28.7 (C3a-CH_3), 27.2 (C5b-CH_3), 26.5 (C8), 23.9 (C5b-CH_3), 10.1 (CH_2Br); ν_{max}/ cm^{-1} (oil): 2922, 1725, 1415, 1338; *m/z* (ES): (Found: $[M-Br]^+$, 193.1198. $C_{11}H_{18}BBrO_2$ requires *[M-Br]*, 193.1120). $[\alpha]_D$ = + 35.2° (c 0.50, $CHCl_3$).

Synthesis of *tert*-butyl 3-bromo-2-methoxybenzoate (S5)

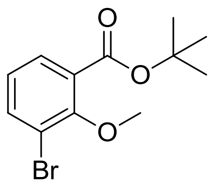

3-Bromo-2-methoxybenzoate (S4) (0.50 g, 2.17 mmol) was dissolved in DCM (10 mL). To this solution was added oxalyl chloride (0.37 mL, 4.34 mmol) and cat. DMF (2 drops); the reaction mixture was stirred for 1 h, then concentrated *in vacuo*. The residue was dissolved in *tert*-butanol and refluxed for 16 h. The solvent was removed *in vacuo*. The crude product was purified by flash column chromatography eluting with 3:1 Petrol–EtOAc. The appropriate fractions were collected, combined and concentrated to afford *tert*-butyl 3-bromo-2-methoxybenzoate **(S5)** as a colourless oil which was used without further purification (0.29 g, 1.02 mmol, 47%) R_f: 0.85 (3:1 Petrol–EtOAc); (Found: C, 50.4; H, 5.30; $C_{12}H_{15}BrO_3$ requires C, 50.3; H, 5.27%); δ_H (300 MHz, $CDCl_3$): 7.68 (2H, app. d, *J* 7.7, 6-H and 4-H), 7.02 (1H, app. t, *J* 7.7, 5-H), 3.93 (3H, s, OCH_3), 1.59 (9H, s, CH_3); δ_C (75 MHz, $CDCl_3$): 164.8 (C=O), 156.2 (C2), 136.5 (C4), 130.4 (C6), 128.9 (C1), 124.9 (C5), 118.9 (C3), 82.1 ($\underline{C}(CH_3)$), 62.0 (OCH_3), 28.2 ($(CH_3)_3$); ν_{max}/ cm^{-1} (oil): 2978, 1721, 1590, 1463, 1302; *m/z* (ES): (Found: MNa^+, 309.0098. $C_{12}H_{15}BrO_3$ requires *MNa*, 309.0097).

Synthesis of 2-methoxy-3-{[(3a*S*,4*S*,6*S*,7a*R*)-3a,5,5-trimethylhexahydro-4,6-methano-1,3,2-benzodioxaborol-2-yl]methyl} benzoic acid tert-butyl ester (S6)

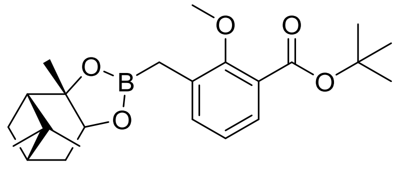

To a stirred solution of tert-butyl 3-bromo-2-methoxybenzoate (S5) (2.63 g, 9.19 mmol) in THF (50 mL) was added *n*-butyllithium (1.6M *n*-hexane solution, 5.77 mL, 9.19 mmol) drop wise at -100 °C. The solution was stirred at −100 °C for 1 h. (3a*S*,4*S*,6*S*,7a*R*)-2-(bromomethyl)-3a,5,5-trimethylhexahydro-4,6-methano-1,3,2-benzodioxaborole (S3) (3.0 g, 11.03 mmol) was added and the reaction mixture was stirred at −100 °C for 2 h. The mixture was then allowed to warm to room temperature with stirring overnight. The mixture was then concentrated *in vacuo*. The residue was diluted with water (50 mL) and extracted with

DCM (3 × 50 mL). The combined organics were dried ($MgSO_4$) and concentrated *in vacuo* to yield the crude product as a pale yellow oil. The crude product was by flash column chromatography eluting with 19:1 Petrol–EtOAc. The appropriate fractions were collected, combined and concentrated to afford desired product (S6) as a colourless oil which was used without further purification (2.79 g, 6.98 mmol, 76%). R_f: 0.85 (19:1 Petrol−EtOAc); δ_H (300 MHz, $CDCl_3$): 7.52 (1H, dd, *J* 7.6 and 1.8, 6-HAr), 7.33 (1H, dd, *J* 7.6 and 1.8, 4-HAr), 7.02 (1H, app. t, *J* 7.6, 5-HAr), 4.27 (1H, dd, *J* 8.7 and 2.0, 7a-H), 3.81 (3H, s, OCH_3), 2.32 (2H, s, CH_2B), 2.32-2.24 (1H, m, 7*-H), 2.22-2.14 (1H, m, 8-H), 2.03 (1H, app. t, *J* 5.1, 4-H), 1.91-1.87 (1H, m, 6-H), 1.82 (1H, dd, *J* 14.2 and 2.1, 7*-H), 1.59 (9H, s, $C(\underline{CH_3})_3$), 1.38 (3H, s, CH_3-C3a), 1.27 (3H, s, CH_3-5b), 1.19 (1H, d, *J* 10.8, 8-H), 0.83 (3H, s, CH_3-5a); δ_C (75 MHz, $CDCl_3$): 166.0 (C=O), 157.4 (C2-Ar), 134.2 (C6-Ar), 134.0 (C3-Ar), 128.4 (C5-Ar), 126.4 (C1-Ar), 123.3 (C4-Ar), 85.8 (C3a), 81.0 ($\underline{C}(CH_3)_3$), 77.9 (C7), 61.5 (OCH_3), 51.3 (C4), 41.3 (C6), 39.5 (C5), 38.2 (C7), 28.6 (C3a-CH_3), 28.2 ($C(\underline{C}H_3)_3$), 27.1 (C5b-CH_3), 24.0 (C8), 22.6 (C5a-CH_3), 11.4 (br, CH_2B); ν_{max}/ cm^{-1} (oil): 2977, 2925, 2869, 1700, 1278, 1135; HPLC: T_r= 2.79 (93% rel. area); *m/z* (ES): (Found: MH^+, 401.2505. $C_{24}H_{34}BClO_5$ requires *MH*, 401.2498). $[\alpha]_D$ = + 12.4° (c 0.50, $CHCl_3$).

Synthesis of 2-methoxy-3-{(2*S*)-2-chloro-2-[(3a*S*,4*S*,6*S*,7a*R*)-3a,5,5-trimethylhexahydro-4,6-methano-1,3,2-benzodioxaborol-2-yl]ethyl} benzoic acid tert-butyl ester (S7)

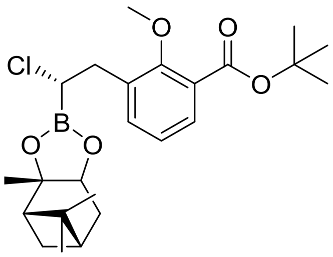

Lithium diisopropylamide (LDA) was freshly prepared by addition of *n*-butyllithium (1.6M *n*-hexane solution, 0.98 mL, 1.50 mmol) to a solution of diisopropylamine (0.21 mL, 1.50 mmol) in THF (3.5 mL) at -100 °C under a nitrogen atmosphere. The mixture was stirred at −100 °C for 10 min before warming to ca. −20 °C. In a separate flask a mixture of 2-methoxy-3-{[(3a*S*,4*S*,6*S*,7a*R*)-3a,5,5-trimethylhexahydro-4,6-methano-1,3,2-benzodioxaborol-2-yl]methyl} benzoic acid tert-butly ester (S6) (0.50 g, 1.25 mmol), DCM (0.32 mL, 5.00 mmol) in THF (22.5 mL) was stirred at −100 °C under nitrogen. To this was added the LDA solution over 30 mins. Stirring was continued at −100 °C for 30 mins, before addition of 1M $ZnCl_2$ solution in ether (2.00 mL, 2.00 mmol). The resulting mixture was allowed to warm to room temperature overnight. The mixture was then concentrated *in vacuo* and sat. NH_4Cl solution (50 mL) was added and the resulting mixture stirred for 10

mins. The mixture was extracted with hexane (3 × 25 mL). The organics were combined, dried ($MgSO_4$) and concentrated *in vacuo* to afford the desired product (S7) as a pale yellow oil (0.51 g, 1.14 mmol, 91%) R_f: 0.50 (19:1 Petrol−EtOAc); δ_H (300 MHz, $CDCl_3$): 7.63 (1H, dd, *J* 7.7 and 1.8 6-HAr), 7.39 (1H, dd, *J* 7.7 and 1.8, 4-HAr), 7.06 (1H, app. t, *J* 7.7, 5-HAr), 4.36 (1H, dd, *J* 8.5 and 2.1, 7a-H), 3.86 (3H, s, OCH_3), 3.74 (1H, t, *J* 8.2, ClCH), 3.20 (2H, dd, *J* 14.1 and 8.2 CH2Ar), 2.45-2.27 (1H, m, 7*-H), 2.25-2.18 (1H, m, 8-H), 2.06 (1H, app. t, *J* 5.5, 6-H), 1.95-1.83 (2H, m, 7*-H and 4-H), 1.59 (9H, s, $(CH_3)_3$), 1.38 (3H, s, C3a-CH_3), 1.28 (3H, s, C5b-CH_3), 1.10 (1H, d, *J* 11.2, 8-H), 0.83 (3H, s, C5a-CH_3); δ_C (75 MHz, $CDCl_3$): 165.7 (C=O), 158.3 (C2-Ar), 134.4 (C6-Ar), 132.7 (C3-Ar), 130.3 (C5-Ar), 126.4 (C1-Ar), 123.2 (C4-Ar), 86.8 (C3a), 81.3 ($C(CH_3)_3$), 78.5 (C7), 62.3 (OCH_3), 51.2 (C4), 41.2 (br, ClCH), 39.4 (C6), 38.2 (C5), 35.1 (C7), 35.0 (CH_2Ar), 28.3 (C3a-CH_3), 28.2 ($C(CH_3)_3$), 27.0 (C5b-CH_3), 26.8 (C8), 24.0 (C5a-CH_3); ν_{max}/ cm^{-1} (oil): 2971, 2921, 2871, 1700, 1466, 1369, 1239, 1135; *m/z* (ES): (Found: MH^+, 449.2233. $C_{24}H_{34}BClO_5$ requires *MH*, 449.2268). $[\alpha]_D$ = + 52.1° (c 0.50, $CHCl_3$).

Synthesis of 2-methoxy-3-{(2*R*)-2-[bis(trimethylsilyl)amino]-2-[(3a*S*,4*S*,6*S*,7a*R*)-3a,5,5-trimethylhexahydro-4,6-methano-1,3,2-benzodioxaborol-2-yl]ethyl} benzoic acid tert-butyl ester (S8)

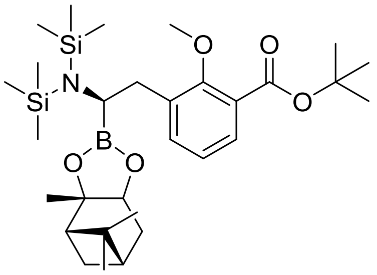

To a stirred solution of 2-methoxy-3-{(2*S*)-2-chloro-2-[(3a*S*,4*S*,6*S*,7a*R*)-3a,5,5-trimethylhexahydro-4,6-methano-1,3,2-benzodioxaborol-2-yl]ethyl} benzoic acid tert-butly ester (S7) (0.45g, 1.00 mmol) in THF (17.5 mL) at −100 °C was added a solution of LiHMDS (1M in THF, 1.1 mL) in THF (7 mL) over 30 mins. The resulting mixture was left to warm to room temperature with stirring overnight. The mixture was concentrated *in vacuo,* then re-suspended in hexane (50 mL) with stirring for 1 h. The suspension was filtered through celite and the celite was washed with further hexane (2 × 25 mL). The filtrate was concentrated *in vacuo* to afford desired product (S8) as a dense pale yellow oil which was used without further purification. (0.33 g, 0.58 mmol, 58%) R_f: 0.70 (19:1 Petrol−EtOAc); δ_H (300 MHz, $CDCl_3$): 7.48 (1H, dd, *J* 7.6 and 1.8, 6-HAr), 7.27 (1H, dd, *J* 7.6 and 1.8, 4-HAr), 6.94 (1H, app. t, *J* 7.6, 5-HAr), 4.18 (1H dd, *J* 8.7 and 1.9, 7a-H), 3.77 (3H, s, OCH_3), 3.15 (1H, dd, *J* 13.2 and 6.6, CH_2Ara), 2.89 (1H, dd, *J* 8.4 and 6.6, NCH), 2.49 (1H, dd, *J* 13.2 and 8.4, CH_2Arb), 2.28-2.13 (1H, m, 7*-H), 2.10-2.03 (1H, m, 8-H), 1.93 (1H, app. t, *J* 5.6, 4-H), 1.83-1.75 (1H, m, 6-H), 1.70-1.62 (1H, m, 7*-H), 1.54 (9H, s, $C(CH_3)_3$), 1.30

(3H, s, C3a-CH_3), 1.20 (3H, s, C5b-CH_3), 0.94 (1H, d, *J* 10.8, 8-H), 0.76 (3H, s, C5a-CH_3), -0.01 (18H, s, TMS); δ_C (75 MHz, $CDCl_3$): 165.3 (C=O), 157.5 (C2-Ar), 134.9 (C6-Ar), 134.0 (C3-Ar), 128.2 (C5-Ar), 125.6 (C1-Ar), 121.8 (C4-Ar), 84.7 (C3a), 80.1 ($\underline{C}(CH_3)_3$), 77.3 (C7), 61.0 ($O\underline{C}H_3$), 50.5 (C4), 41.2 ($N\underline{C}H$), 35.1 (C7), 35.0 (CH_2Ar), 27.7 (C3a-$\underline{CH_3}$), 27.3 ($C(\underline{CH_3})_3$), 26.2 (C5b-$\underline{CH_3}$), 25.4 (C8), 23.1 (C5b-CH_3); ν_{max}/ cm^{-1} (oil): 2977, 2925, 2869, 1700, 1287, 1135; *m/z* (ES): (Found: $[M\text{-}2TMS]^+$, 430.2783. $C_{30}H_{52}BNO_5Si_2$ requires *[M-2TMS]*, 430.2760). $[\alpha]_D$ = + 20.1° (c 0.50, $CHCl_3$).

Synthesis of 3-[(2*R*)-2-[cyclohexylformamido]-2-[(3a*S*,4*S*,6*S*,7a*R*)-3a,5,5-trimethylhexahydro-4,6-methano-1,3,2-benzodioxaborol-2-yl]-ethyl]-2-methoxy-benzoic acid tert-butyl ester (S9a)

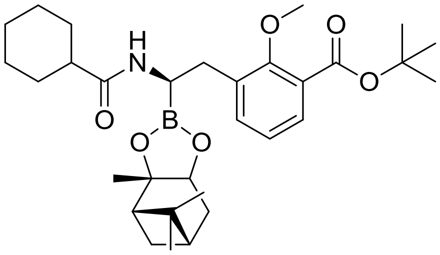

Preparation was *via* general method A using cyclohexane carboxylic acid (0.24 g, 1.80 mmol) and 2-methoxy-3-{(2*R*)-2-[bis(trimethylsilyl)amino]-2-[(3a*S*,4*S*,6*S*,7a*R*)-3a,5,5-trimethylhexahydro-4,6-methano-1,3,2-benzodioxaborol-2-yl]ethyl} benzoic acid tert-butly ester (S8) (1.25 g, 2.20 mmol) to afford the desired product as a yellow glassy solid (0.14 g, 14%); m.p.: 135-138 °C; R_f: 0.15 (3:1 Hexane–EtOAc); δ_H (500 MHz, $CDCl_3$): 8.04 (1H, s, NH), 7.63 (1H, dd, *J* 7.7, 1.6, 6-HAr), 7.37 (1H, dd, *J* 7.7, 1.6, 4-HAr), 7.12 (1H, app. t, *J* 7.7, 5-HAr), 4.30 (1H, d, *J* 6.5, 7a-H), 3.85 (3H, s, OCH_3), 2.92-2.87 (3H, m, CH_2C and NCH), 2.39 (1H, t, *J* 2.5, 1`-H), 2.32-2.30 (1H, m, 7*-H), 2.21-2.18 (2H, m, 2`-H, 6`-H), 2.07 (1H, t, *J* 3.3, 16-H), 1.94-1.90 (4H, m, 2`-H, 3`H, 5`-H, 6`H), 1.81 (2H, d, *J* 12.5, 6-H, 8-H), 1.72-1.69 (1H, m, 7*-H), 1.64 (9H, s, $C(CH_3)_3$), 1.56 (1H, d, *J* 10.1, 4`-H), 1.46 (3H, s, C3-CH_3), 1.40 (1H, d, *J* 6.8, 8-H), 1.32 (3H, s, C5a-CH_3), 1.30-1.25 (2H, m, 5'-H and 3'-H), 1.25-1.20 (1H, m, 4`H), 0.91 (3H, s, C5b-$CH_3$);□ $\delta_C$ (100 MHz, $CDCl_3$): 180.5 (NHC=O), 165.0 (C=O), 158.0 (C2-Ar), 136.0 (C1-Ar), 134.7 (C6-Ar), 129.7 (C4-Ar), 127.5 (C3-Ar), 124.1 (C5-Ar), 83.1 (C3a), 81.5 ($C(CH_3)$), 77.3 (C4), 76.3 (C7a), 62.2 ($OCH_3$), 52.6 (C4), 45.0 (brs, NCH), 40.5 (C1`), 40.3 (C6), 38.2 (C5), 37.0 (CH_2), 32.8 (C7*), 29.2 (C3-CH_3), 28.8 (C6` and C2`), 28.2 ($C(CH_3)_3$), 27.4 (C5a-CH_3), 26.7 (C4), 25.5 (C5`, C4' and C3`), 24.2 (C5b-CH_3); ν_{max}/ cm^{-1} (solid): 3197, 3072, 2975, 2929. 2896, 2856, 1717, 1590; *m/z* (ES): (Found: MH^+, 540.3513. $C_{31}H_{46}BNO_6$ requires *MH*, 540.3513). $[\alpha]_D$ = + 10.2° (c 0.50, $CHCl_3$).

Synthesis of 3-[(2*R*)-2-borono-2-[cyclohexylformido]-ethyl]-2-hydroxy-benzoic acid hydrochloride (1)

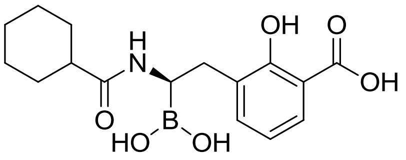

Preparation was *via* general method C using 3-[(2*R*)-2-[cyclohexylformamido]-2-[(3a*S*,4*S*,6*S*,7a*R*)-3a,5,5-trimethylhexahydro-4,6-methano-1,3,2-benzodioxaborol-2-yl]-ethyl]-2-methoxy-benzoic acid tert-butyl ester (S9a) (50 mg, 0.08 mmol) to afford the desired product as a colourless solid which was used without further purification (1.9 mg, 0.01 mmol, 7%). m.p.: > 250 °C; R_f: baseline (9:1 DCM−MeOH); δ_H (500 MHz, MeOD-d4): 7.64 (1H, d, *J* 7.6, 6-HAr), 7.12 (1H, d, *J* 7.6, 4-HAr), 6.78 (1H, app. t, *J* 7.6, 5-HAr), 3.07 (1H, app. s, NCH), 2.73 (2H, dd, *J* 15.5 and 7.1, CH_2), 2.16-2.08 (1H, m, 1-H), 1.45-1.30 (4H, m, 6-H and 2-H), 1.23-1.15 (2H, m, 4-H), 1.11-0.92 (4H, m, 5-H and 3-H); δ_C (100 MHz, MeOD-d4): 185.0 (NHC=O), 169.1 (COOH), 157.2 (C2-Ar), 136.4 (C4-Ar), 131.1 (C6-Ar), 129.1 (C3-Ar), 122.2 (C5-Ar), 118.6 (C1-Ar), 45.0 (br, NCH), 40.1 (C1), 32.3 (CH_2), 29.5 (C6 and C2), 26.2 (C4), 25.6 (C5 and C3); ν_{max}/ cm^{-1} (solid): 2929, 2855, 1713, 1596, 1185; HPLC: T_r= 1.78 (90% rel. area); *m/z* (ES): (Found: $[M\text{-}H_2O\text{+}H]^+$, 318.1516. $C_{16}H_{22}BNO_6$ requires *[M-H$_2$O+H]*, 318.1510. $[\alpha]_D = -32.0°$ (c 0.10, MeOH).

Synthesis of 3-[(2*R*)-2-[4-(tert-Butoxycarbonylamino-methyl)-benzoylamino]-2-[(3a*S*,4*S*,6*S*,7a*R*)-3a,5,5-trimethylhexahydro-4,6-methano-1,3,2-benzodioxaborol-2-yl]-ethyl]-2-methoxy-benzoic acid tert-butyl ester (S9b)

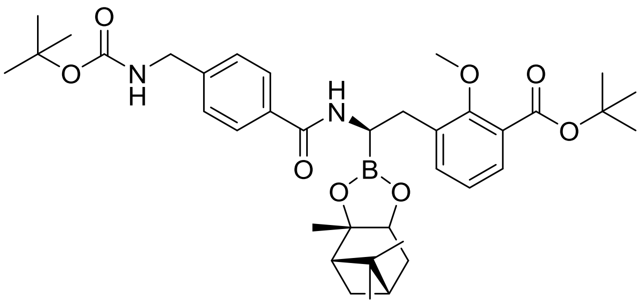

The desired product was prepared by general method A using 4-Boc (aminomethyl)benzoic acid (0.50 g, 1.99 mmol) and 2-methoxy-3-{(2*R*)-2-[bis(trimethylsilyl)amino]-2-[(3a*S*,4*S*,6*S*,7a*R*)-3a,5,5-trimethylhexahydro-4,6-methano-1,3,2-benzodioxaborol-2-yl]ethyl} benzoic acid tert-butly ester (S8) (1.70 g, 3.98 mmol) to afford desired product_as a yellow glassy solid which was used without further purification (0.55 g, 0.84 mmol, 42%). m.p.: 73.8-75.5 °C; R_f: 0.20 (3:1 Hexane−EtOAc); δ_H (500 MHz, $CDCl_3$): 7.73 (2H, d, *J* 8.2 2'-HAr and 6'-HAr), 7.61 (1H, dd, *J* 7.7 and 1.7, 6-HAr), 7.48 (1H, brs, CONH), 7.38 (1H, dd, *J* 7.7 and 1.7, 4-HAr), 7.34 (2H, d, *J* 8.2, 3'-HAr and 6'-HAr), 7.09 (1H, t, *J* 7.7, 5-HAr), 4.94 (1H, brs, BOC-NH), 4.36 (2H, app. s, CH_2N), 4.31 (1H, d, J 7.6, 7a-H), 3.83 (3H, s, OCH_3), 3.09 (1H, t, *J* 5.4, NCH), 2.99 (2H, d, *J* 5.4, CH_2Ar), 2.44-2.30 (1H, m, 7*-H), 2.29-2.12 (1H, m, 8-H), 2.06 (1H, app. t, *J* 10.9, 6-H), 1.98-1.86 (2H, m, 7*-H and 4-H), 1.61 (9H, s, $C(\underline{CH_3})_3$), 1.58 (1H, d, *J* 10.9, 8-H), 1.48 (9H, s, BOC-$\underline{CH_3}$), 1.46 (3H, s, C3-CH_3), 1.30 (3H, s, C5b-CH_3), 0.90 (3H, s, C5a-CH_3); δC(75 MHz, $CDCl_3$): 170.1

(CONH), 165.8 (C=O), 157.6 (C=O BOC), 135.8 (C4'-Ar), 134.8 (C6-Ar and C1'-Ar), 129.8 (C4-Ar), 128.0 (C5'-Ar, C3'-Ar and C3-Ar), 127.5 (C6'-Ar and C2'-Ar), 124.2 (C5-Ar and C1-Ar), 83.7 (C3a), 81.5 ($C(CH_3)_3$), 76.7 (C7a), 62.3 (OCH_3), 52.4 (C4), 45.0 (CH2CB), 40.2 (C6), 38.2 (C5), 36.7 (CH2N), 32.8 (C7), 28.3 (C3-CH3), 28.2 ($(CH_3)_3$), 27.4 (C5a-CH_3), 26.7 (C8), 24.3 (C5b-CH_3); ν_{max}/ cm^{-1} (solid): 3272, 2971, 2870, 1700, 1502, 1388, 1228; *m/z* (ES): (Found: MH^+, 663.4033. $C_{37}H_{51}BN_2O_8$ requires MH, 663.3811). $[\alpha]_D = -22.3°$ (c 0.50, $CHCl_3$)

Synthesis of 3-[(2*R*)-2-(4-(Aminomethyl)benzoylamino)-2-borono-ethyl]-2-hydroxy-benzoic acid hydrochloride (2)

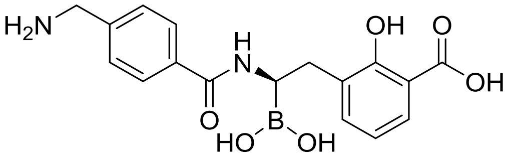

The desired product was prepared by *via* general method C using 3-[(2*R*)-2-[4-(tert-butoxycarbonylamino-methyl)-benzoylamino]-2-[(3a*S*,4*S*,6*S*,7a*R*)-3a,5,5-trimethylhexahydro-4,6-methano-1,3,2-benzodioxaborol-2-yl]-ethyl]-2-methoxy-benzoic acid tert-butyl ester (S9b) (50 mg, 0.08 mmol) to afford the desired product as a colourless solid which was used without further purification (3.8 mg, 0.01 mmol, 13%). m.p.: > 250 °C; R_f: baseline (9:1 DCM−MeOH); δ_H (500 MHz, MeOD-d4): 8.39 (1H, brs, CONH), 8.0-7.85 (2H, app. m, 6'-H and 2'-H), 7.82 (1H, d, *J* 7.8, 6-H), 7.62-7.50 (2H, app. m, 5'-H and 3'-H), 7.38-7.28 (1H, app. m, 4-H), 6.97-6.78 (1H, app. m, 5-H), 4.20 (2H, s, CH_2NH_2), 3.52 (1H, app. s, NCH), 3.09 (2H, app. s, CH_2); ν_{max}/ cm^{-1} (solid): 3388, 2913, 1597, 1481, 1337; *m/z* (ES): (Found: $[M-H_2O+H]^+$, 341.1312. $C_{17}H_{19}BN_2O_6$ requires *[M-H₂O+H]*, 341.1306. $[\alpha]_D = -75.4°$ (c 0.10, MeOH).

Synthesis of 3-[(2*R*)-2-[4-(tert-Butoxycarbonylamino-methyl)-cyclohexyl formamido]-2-[(3a*S*,4*S*,6*S*,7a*R*)-3a,5,5-trimethylhexahydro-4,6-methano-1,3,2-benzodioxaborol-2-yl]-ethyl]-2-methoxy-benzoic acid tert-butyl ester (S9c)

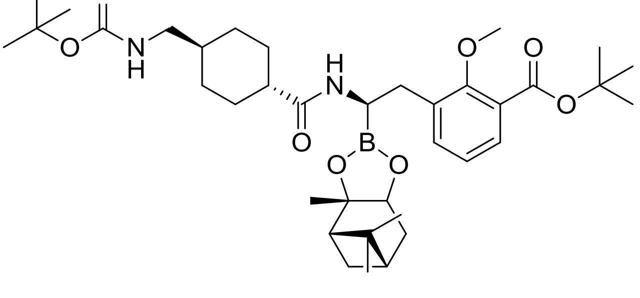

The desired product was prepared *via* general method A using 4-ter0butyloxycarbonyl-aminomethylcyclohexyl carboxylic acid (0.36 g, 1.40 mmol) and 2-methoxy-3-{(2*R*)-2-[bis(trimethylsilyl)amino]-2-[(3a*S*,4*S*,6*S*,7a*R*)-3a,5,5-trimethylhexahydro-4,6-methano-1,3,2-benzodioxaborol-2-yl]ethyl} benzoic acid tert-butly ester (S8) (0.80 g, 1.40 mmol) to afford the desired product as a colourless oil which was used without further

purification (0.15 g, 0.22 mmol, 16%). R_f: 0.20 (3:1 Hexane−EtOAc); δ_H (500 MHz, $CDCl_3$): 7.58 (1H, dd, *J* 7.9 and 1.4, 6-HAr), 7.32 (1H, dd, *J* 7.9 and 1.4, 4-HAr), 7.08 (1H, app. t, *J* 7.9, 5-HAr), 6.85 (1H, brs, CONH), 4.56 (1H, brs, BOC NH), 4.23 (1H, d, *J* 6.0, 7a-H), 3.81 (3H, s, OCH_3), 2.97 (2H, t, *J* 7.1, CH_2NH), 2.84 (2H, d, *J* 8.9, CH_2C), 2.39-2.32 (1H, m, 7*-H), 2.20-2.11 (2H, m, 8-H and 1'-H), 2.03-1.99 (1H, m, 6-H), 1.97-1.80 (6H, m, 7*-H, 4-H, 6'-H and 2'-H), 1.65-1.63 (1H, m, 4'-H), 1.61 (9H, s, $C(CH_3)_3$) 1.50 (1H, d, *J* 7.7, 8-H), 1.46 (9H, s, CH_3 BOC), 1.48-1.37 (4H, m, 5'-H and 3'-H), 1.40 (3H, s, C3-CH_3), 1.28 (3H, s, C5a-CH_3), 0.89 (3H, s, C5b-CH_3); δ_C (100MHz, $CDCl_3$): 179.3 (HNC=O), 165.8 (^{t}BuOC=O), 157.7 (C2-Ar), 156.0 (BOC C=O), 135.6 (C1-Ar), 134.5 (C6-Ar), 129.5 (C4-Ar), 127.1 (C3-Ar), 123.8 (C5-Ar), 83.5 (C3), 81.3 ($\underline{C}(CH_3)_3$ × 2), 76.4 (C7a), 62.0 (OCH_3), 52.6 (C4), 46.4 (CH_2N), 45.0 (NCH), 41.4 (C1'), 40.3 (C6), 38.2 (C5), 37.8 (C4'), 36.8 (CH_2C), 32.5 (C7), 29.6 (C6' and C2'), 29.4 (C3-CH_3), 28.4 (BOC $C(CH_3)_3$), 28.3 (C5' and C3'), 28.2 ($C(CH_3)_3$), 27.4 (C5a-CH_3), 26.6 (C8), 24.1 (C5b-CH_3); ν_{max}/ cm^{-1} (oil): 2973, 2925, 1698, 1365, 1228; HPLC: T_r= 3.11 (100% rel. area); *m/z* (ES): (Found: MH^+, 669.4285. $C_{37}H_{57}BN_2O_8$ requires *MH*, 669.4287). $[\alpha]_D$ = − 9.04° (c 0.50, $CHCl_3$).

Synthesis of 3-[(2*R*)-2-[4-(aminomethyl)-cyclohexylformido]-2-borono-ethyl]-2-hydroxy-benzoic acid hydrochloride (3)

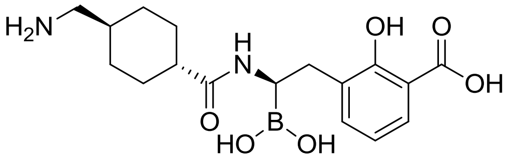

Preparation was *via* general method C using 3-[(2*R*)-2-[4-(tert-Butoxycarbonylamino-methyl)-cyclohexyl formamido]-2-[(3a*S*,4*S*,6*S*,7a*R*)-3a,5,5-trimethylhexahydro-4,6-methano-1,3,2-benzodioxaborol-2-yl]-ethyl]-2-methoxy-benzoic acid tert-butyl ester (S9c) (50 mg, 0.07 mmol) to afford the desired product as a colourless solid which was used without further purification (2.2 mg, 0.01 mmol, 8%). m.p.: 49.2-50.1 °C; R_f: baseline (9:1 DCM−MeOH); δ_H (500 MHz, MeOD-d4): 8.36 (1H, brs, HNC=O), 7.71 (1H, app. s, 6-HAr), 7.20 (1H, app. s, 4-HAr), 6.86 (1H, app. s, 5-HAr), 4.47 (2H, brs, $B(OH)_2$), 3.16 (1H, brs, HCN), 2.83 (2H, s, CH_2), 2.67 (2H, dd, *J* 7.0 and 1.8, CH_2NH_2), 2.19 (1H, app. s, 1'-H), 1.74-1.63 (4H, m, 6'-H and 2'-H), 1.50-1.43 (1H, m, 4'-H), 1.35-1.20 (2H, m, 5a'-H and 3a'-H), 0.95-0.87 (2H, m, 5b'-H and 3b'-H); ν_{max}/ cm^{-1} (solid): 2928, 2858, 1718, 1595, 1427, 1227; *m/z* (ES): (Found: $[M-H_2O+H]^+$, 347.1782. $C_{17}H_{25}BN_2O_6$ requires *[M-H₂O+H]*, 347.1776. $[\alpha]_D$ = − 46.0 ° (c 0.10, MeOH).

Synthesis of 3-[(2*R*)-2-[4-(bromomethyl)-benzoylamino]-2-[(3a*S*,4*S*,6*S*,7a*R*)-3a,5,5-trimethylhexahydro-4,6-methano-1,3,2-benzodioxaborol-2-yl]-ethyl]-2-methoxy benzoic acid tert-butyl ester (S9d)

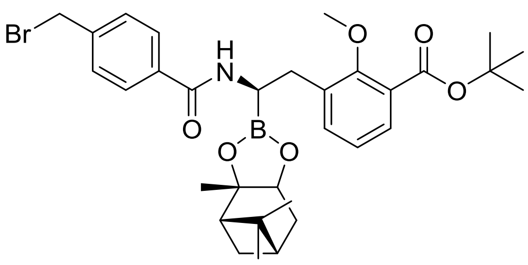

The desired product was prepared *via* general method A using 4-bromomethylbenzoic acid (0.99 g, 4.64 mmol) and 2-methoxy-3-{(2*R*)-2-[bis(trimethylsilyl)amino]-2-[(3a*S*,4*S*,6*S*,7a*R*)-3a,5,5-trimethylhexahydro-4,6-methano-1,3,2-benzodioxaborol-2-yl]ethyl} benzoic acid tert-butly ester (S8) (3.19 g, 5.57 mmol) to afford the desired product as a yellow glassy solid which was used without further purification (0.50 g, 0.80 mmol, 14%). m.p.: 93.7-95.2 °C; R_f: 0.30 (3:1 Hexane−EtOAc); δ_H (500 MHz, $CDCl_3$): 7.72 (2H, d, *J* 7.8, 2'-HAr and 6'-HAr), 7.61 (1H, d, *J* 6.9, 6-HAr), 7.44 (3H, app. d, *J* 7.8, CONH, 3'-HAr and 5'-HAr), 7.36 (1H, d, *J* 6.9, 4-HAr), 7.10 (1H, t, *J* 6.9, 5-HAr), 4.48 (2H, s, CH_2Br), 4.32 (1H, d, *J* 9.0, 7a-H), 3.84 (3H, s, OCH_3), 3.15 (1H, t, *J* 6.9, NCH), 3.00 (2H, d, *J* 6.9, CH_2Ar), 2.48-2.29 (1H, m, 7*-H), 2.25-2.16 (1H, m, 8-H), 2.07-1.99 (1H, m, 6-H), 1.97-1.83 (2H, m, 7*-H and 4-H), 1.62 (9H, s, $C(\underline{CH_3})_3$), 1.52 (1H, d, *J* 7.4, 8-H), 1.74 (3H, s, C3-CH_3), 1.28 (3H, C5b-CH_3), 0.89 (3H, s, C5a-CH_3); δ_C (100 MHz, $CDCl_3$): 169.5 (HNC=O), 165.8 (C=O), 157.6 (C2-Ar), 142.5 (C4'-Ar), 135.5 (C1'-Ar), 134.7 (C6-Ar), 129.9 (C4-Ar), 129.4 (C3-Ar), 129.3 (C6'-Ar and C2'-Ar), 128.1 (C5'-Ar and C3'-Ar), 124.2 (C5-Ar), 84.1 (C3a), 81.5 (C7a), 77.0 ($\underline{C}(CH_3)_3$),62.3 (OCH_3), 52.5 (C4), 45.0 (brs, NCH), 38.2 (C5), 36.5 (CH_2C), 32.6 (C7), 32.0 (CH_2Br), 29.1 (C3-CH_3), 27.4 (C5a-CH_3), 26.6 (C8), 24.2 (C5b-CH_3); ν_{max}/ cm^{-1} (solid): 2720, 1700, 1367, 1039, 1003; *m/z* (ES): (Found: MH^+, 626.2780. $C_{32}H_{41}BBrNO_6$ requires *MH*, 626.2283. $[\alpha]_D$ = + 14.3° (c 0.50, $CHCl_3$).

Synthesis of 2-methoxy-3-[(2*R*)-2-((4-Boc-piperazinylmethyl)-benzoylamino)-2-[(3a*S*,4*S*,6S,7a*R*)-3a,5,5-trimethylhexahydro-4,6-methano-1,3,2-benzodioxaborol-2-yl]-ethyl] benzoic acid tert-butyl ester (S10a)

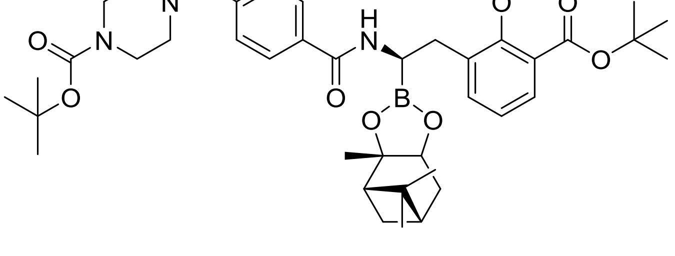

The desired product was prepared *via* general method B using 3-3-[(2*R*)-2-[4-(bromomethyl)-benzoylamino]-2-[(3a*S*,4*S*,6*S*,7a*R*)-3a,5,5-trimethylhexahydro-4,6-methano-1,3,2-benzodioxaborol-2-yl]-ethyl]-2-methoxy benzoic acid tert-butyl ester (0.41 g, 0.65 mmol) (S9d) and 1-Boc-piperazine (0.12 g, 0.65 mmol) to afford the desired

product as a red oil which was used without further purification (80 mg, 0.11 mmol, 17%). R_f: 0.15 (3:1 Hexane−EtOAc); δ_H (500 MHz, $CDCl_3$): 7.72 (2H, d, *J* 8.1, 6’-HAr and 2’-HAr), 7.61 (1H, dd, *J* 7.3 and 1.3, 6-HAr), 7.45 (1H, brs, CON$\underline{H}$), 7.39 (2H, d, *J* 8.1, 5’-HAr and 3’-HAr), 7.38 (1H, dd, *J* 7.3 and 1.3, 4-HAr), 7.10 (1H, app.t, *J* 7.3, 5-HAr), 4.31 (1H, d, *J* 9.0, 7a-H), 3.83 (3H, s, OCH_3), 3.54 (2H, s, CH_2), 3.46-3.39 (4H, m, 5-HPip and 3-HPip), 3.09 (1H, t, *J* 6.3, NHC), 3.00 (2H, d, *J* 6.3, CH_2C), 2.43-2.31 (5H, m, 6-HPip, 2-HPip and 7*-H), 2.24-2.14 (1H, m, 8-H), 2.08-2.00 (1H, m, 6-H), 1.95-1.86 (2H, m, 7*-H and 4-H), 1.61 (9H, s, $C(CH_3)_3$), 1.48 (3H, s, C3-CH_3), 1.46 (9H, s, BOC), 1.30 (C5a-CH_3), 1.26 (1H, d, *J* 7.4, 8-H), 0.89 (3H, s, C5b-CH_3); δ_C (100 MHz, $CDCl_3$): 170.3 (NHC=O), 165.8 (C=O), 154.8 (BOC C=O), 143.8 (C4’-Ar), 135.9 (C1’-Ar), 134.8 (C6-Ar), 129.8 (C4-Ar), 129.9 (C6’-Ar, C2’-Ar and C3-Ar), 127.8 (C5’-Ar and C3’-Ar), 127.7 (C1-Ar), 124.1 (C5-Ar), 83.7 (C3a), 81.5 ($\underline{C}(CH_3)_3$), 79.6 (BOC C), 77.0 (C7a), 62.5 (CH_2), 62.3 (OCH_3) 52.9 (C6-Pip, C5-Pip, C3-Pip and C2-Pip), 52.4 (C4), 45.0 (brs, NCH), 40.2 (C6), 38.2 (CH_2C), 36.8 (C5), 32.8 (C7), 29.2 (C3-CH_3), 28.3 ($C(\underline{C}H_3)_3 \times 2$), 27.3 (C5a-$CH_3$) 26.7 (C8), 24.2 (C5b-$CH_3$); ν_{max}/ cm^{-1} (oil): 2850, 1700, 1390, 1252, 1030; *m/z* (ES): (Found: MH^+, 732.4394. $C_{41}H_{58}BN_3O_8$ requires *MH*, 732.4397). $[\alpha]_D = -65.6°$ (c 0.50, $CHCl_3$).

Synthesis of 3-[(2*R*)-2-borono-2-(4-(1-piperazinylmethyl)benzoylamino)-ethyl]-2-hydroxy-benzoic acid hydrochloride (4)

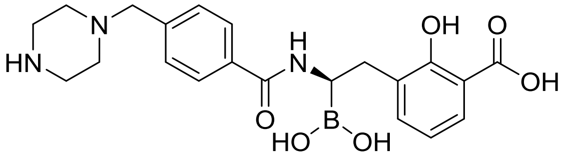

The desired product (4) was prepared via general method C using 2-methoxy-3-[(2*R*)-2-((4-Boc-piperazinylmethyl)-benzoylamino)-2-[(3a*S*,4*S*,6*S*,7a*R*)-3a,5,5-trimethylhexahydro-4,6-methano-1,3,2-benzodioxaborol-2-yl]-ethyl] benzoic acid tert-butyl ester (S10a) (50 mg, 0.07 mmol) to afford the desired product as an off-white glassy solid which was used without further purification (0.7 mg, 0.002 mmol, 2%). m.p.: 36.3-37.6 °C; R_f: baseline (9:1 DCM−MeOH); δ_H (500 MHz, MeOD-d4): 8.28 (1H, brs, CONH), 7.75-7.70 (2H, app. m, 6’-H and 2’-H), 7.61 (1H, d, *J* 7.6, 6-H), 7.44-7.36 (2H, app. m, 5’-H and 3’-H), 7.23-7.18 (1H, app. m, 4-H), 6.84-6.78 (1H, app. m, 5-H), 4.47 (2H, brs, $B(OH)_2$), 3.55 (2H, s, CH_2N), 3.36 (1H, brs, CHN), 3.13-3.04 (4H, m, 5’’-H and 3’’-H), 2.96 (2H, app. s, CH_2), 2.58-2.50 (4H, m, 6’’-H and 2’’-H); ν_{max}/ cm^{-1} (solid): 3358, 1670, 1610, 1478, 1018; *m/z*

(ES): (Found: $[M-H_2O+H]^+$, 410.1891. $C_{21}H_{26}BN_3O_6$ requires *$[M-H_2O+H]$*, 410.1895. $[\alpha]_D$ = − 46.0° (c 0.05, MeOH).

Synthesis of 3-[(2*R*)-2-[((Boc-ethylamine)methyl)-benzoylamino]-2-[(3a*S*,4*S*,6*S*,7a*R*)-3a,5,5-trimethylhexahydro-4,6-methano-1,3,2-benzodioxaborol-2-yl]-ethyl]-2-methoxy benzoic acid tert-butyl ester (S10b)

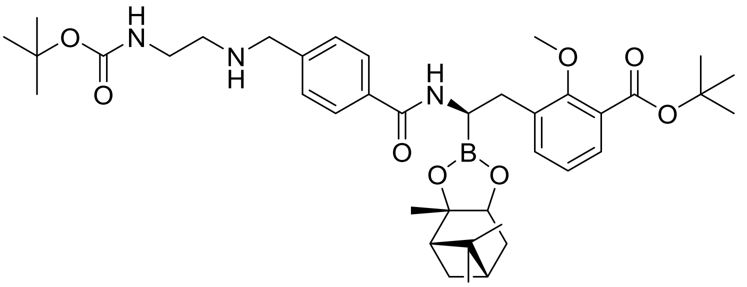

The desired product was prepared *via* general method B using 3-[(2*R*)-2-[4-(bromomethyl)-benzoylamino]-2-[(3a*S*,4*S*,6*S*,7a*R*)-3a,5,5-trimethylhexahydro-4,6-methano-1,3,2-benzodioxaborol-2-yl]-ethyl]-2-methoxy benzoic acid tert-butyl ester (S9d) (0.60 g, 0.96 mmol) and N-Boc-ethylenediamine (0.15 g, 0.96 mmol) to afford the desired product as a pale brown oil which was used without further purification (0.12 g, 0.17 mmol, 18%). R_f: 0.20 (3:1 Hexane−EtOAc); δ_H (500MHz, $CDCl_3$): 7.67 (2H, d, *J* 7.6, 6'-HAr and 2'-HAr), 7.51 (1H, dd, *J* 6.5 and 1.3, 6-HAr), 7.36 (2H, d, *J* 7.6, 5'-HAr and 3'-HAr), 7.29 (1H, dd, *J* 6.5 and 1.3, 4-HAr), 7.01 (1H, app. t, *J* 6.5, 5-HAr), 5.86 (1H, brs, BOCNH), 4.20 (1H, d, *J* 7.7, 7a-H), 3.99 (2H, s, Ar$\underline{CH_2}$NH), 3.76 (3H, s, OCH_3), 3.26 (2H, d, *J* 5.5, CH_2C), 3.10 (1H, brs, NH), 2.95-2.85 (4H, m, HN$\underline{CH_2}$ and $\underline{CH_2}$NH), 2.31-2.24 (1H, m, 7*-H), 2.13-2.06 (1H, m, 8-H), 1.96-1.91 (1H, m, 6-H), 1.85-1.77 (2H, m, 7*-H and 4-H), 1.54 (9H, s, $C(CH_3)_3$), 1.41 (1H, d, *J* 7.4, 8-H), 1.34 (3H, s, C3-CH_3), 1.30 (9H, s, BOC $C(CH_3)_3$), 1.20 (3H, s, C5a-CH_3), 0.78 (3H, s, C5b-CH_3); δ_C (100MHz, $CDCl_3$): 169.8 (NHC=O), 166.3 (BOC C=O), 165.9 (C=O), 157.8 (C2-Ar), 135.3 (C1'-Ar), 134.6 (C6-Ar), 130.0 (C6'-Ar and C2'-Ar), 129.9 (C3-Ar), 129.7 (C4-Ar), 128.5 (C5'-Ar and C3'-Ar), 126.8 (C1-Ar), 124.0 (C5-Ar), 84.0 (C3a), 81.5 ($C(CH_3)_3$ × 2), 76.7 (C7a), 62.2(OCH_3), 52.2 (C4), 50.6 (CH_2NH), 47.4 (CH_2NH), 45.0 (brs HCN), 40.1 (C6), 38.2 (C5), 36.4 (CH_2C), 32.4 ($ArCH_2NH$), 29.1 (C3-CH_3), 28.3 ($C(CH_3)_3$ × 2), 27.3 (C5a-CH_3), 24.2 (C5b-CH_3); ν_{max}/ cm^{-1} (oil): 2920, 2914, 1700, 1652, 1080, 730 *m/z* (ES): (Found: MH^+, 706.4222. $C_{39}H_{56}BN_3O_8$ requires *MH*, 706.4240). $[\alpha]_D$ = − 147.0° (c 0.50, $CHCl_3$).

Synthesis of 3-[(2*R*)- 2-[(4-(2-amino-ethylamino)-methyl)benzoylamino]-2-borono-ethyl]-2-hydroxy-benzoic acid hydrochloride (5)

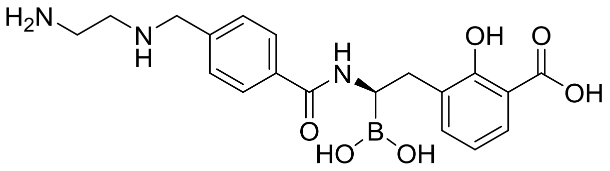

The desired product was prepared *via* general method C using 3-[(2*R*)-2-((Boc-ethylamine)-benzoylamino)-2-[(3a*S*,4*S*,6*S*,7a*R*)-3a,5,5-trimethylhexahydro-4,6-methano-1,3,2-benzodioxaborol-2-yl]-ethyl]-2-methoxy benzoic acid tert-butyl ester (S10b) (0.10 g, 0.14 mmol) to afford the desired product as a yellow glassy solid which was used without further purification (0.5 mg, 0.001 mmol, 1%). m.p.: 44.7-45.9 °C; R_f: baseline (9:1 DCM−MeOH); δ_H (500 MHz, MeOD-d4): 8.28 (1H, brs, CONH), 7.75-7.71 (2H, app. m, 6'-H and 2'-H), 7.70 (1H, d, *J* 7.6, 6-H), 7.44-7.41 (2H, m, 5'-H and 3'-H), 7.24-7.18 (1H, app. m, 4-H), 6.84-6.78 (1H, app. m, 5-H), 4.47 (2H, brs, $B(OH)_2$), 3.75 (2H, s, CH_2N), 3.39 (1H, app. s, NCH), 2.96 (2H, app. s, CH_2Ar), 2.86 (2H, app. t, *J* 5.7, Ethyl CH_2), 2.68 (2H, app. t, J 5.7, Ethyl-CH_2); ν_{max}/ cm^{-1} (solid): 2892, 1717, 1662, 1530, 1389, 921; *m/z* (ES): (Found: $[M\text{-}H_2O+H]^+$, 384.1725. $C_{19}H_{24}BN_3O_6$ requires *[M-H_2O+H]*, 384.1729. $[\alpha]_D = -15.4$ ° (c 0.10, MeOH).

Supplementary References

1. Rydzik A. M., *et al.* Monitoring Conformational Changes in the NDM-1 Metallo-β-lactamase by ^{19}F NMR Spectroscopy. *Angew. Chem. Int. Ed.* **53**, 3129-3133 (2014).

2. Wallace A. C., Laskowski R. A., Thornton J. M. LIGPLOT: a program to generate schematic diagrams of protein-ligand interactions. *Protein Eng.* **8**, 127-134 (1995).

3. McKinney D. C., *et al.* 4,5-Disubstituted 6-Aryloxy-1,3-dihydrobenzo[c][1,2]oxaboroles Are Broad-Spectrum Serine β-Lactamase Inhibitors. *ACS Infect. Diseases* **1**, 310-316 (2015).

4. Ness S., *et al.* Structure-based design guides the improved efficacy of deacylation transition state analogue inhibitors of TEM-1 β-Lactamase. *Biochemistry* **39**, 5312-5321 (2000).

5. Hecker S. J., *et al.* Discovery of a Cyclic Boronic Acid β-Lactamase Inhibitor (RPX7009) with Utility vs Class A Serine Carbapenemases. *J. Med. Chem.* **58**, 3682-3692 (2015).

6. Nicola G., Peddi S., Stefanova M., Nicholas R. A., Gutheil W. G., Davies C. Crystal Structure of Escherichia coli Penicillin-Binding Protein 5 Bound to a Tripeptide Boronic Acid Inhibitor: A Role for Ser-110 in Deacylation. *Biochemistry* **44**, 8207-8217 (2005).

7. Nicholas RA, Krings S, Tomberg J, Nicola G, Davies C. Crystal structure of wild-type penicillin-binding protein 5 from Escherichia coli: implications for deacylation of the acyl-enzyme complex. *J. Biol. Chem.* **278**, 52826-52833 (2003).

8. Nicola G., Tomberg J., Pratt R. F., Nicholas R. A., Davies C. Crystal Structures of Covalent Complexes of β-Lactam Antibiotics with Escherichia coli Penicillin-Binding Protein 5: Toward an Understanding of Antibiotic Specificity. *Biochemistry* **49**, 8094-8104 (2010).

9. Murai N., Miyano M., Yonaga M., Tanaka K. One-Pot Primary Aminomethylation of Aryl and Heteroaryl Halides with Sodium Phthalimidomethyltrifluoroborate. *Org. Lett.* **14**, 2818-2821 (2012).

10. Inglis S. R., Strieker M., Rydzik A. M., Dessen A., Schofield C. J. A boronic-acid-based probe for fluorescence polarization assays with penicillin binding proteins and β-lactamases. *Anal Biochem* **420**, 41-47 (2012).
